# Mitochondrial respiration and ROS emission during β-oxidation in the heart: An experimental-computational study

**DOI:** 10.1371/journal.pcbi.1005588

**Published:** 2017-06-09

**Authors:** Sonia Cortassa, Steven J. Sollott, Miguel A. Aon

**Affiliations:** Laboratory of Cardiovascular Science, National Institute on Aging, National Institutes of Health, Baltimore, MD, United States of America; University of California San Diego, UNITED STATES

## Abstract

Lipids are main fuels for cellular energy and mitochondria their major oxidation site. Yet unknown is to what extent the fuel role of lipids is influenced by their uncoupling effects, and how this affects mitochondrial energetics, redox balance and the emission of reactive oxygen species (ROS). Employing a combined experimental-computational approach, we comparatively analyze β-oxidation of palmitoyl CoA (PCoA) in isolated heart mitochondria from Sham and streptozotocin (STZ)-induced type 1 diabetic (T1DM) guinea pigs (GPs). Parallel high throughput measurements of the rates of oxygen consumption (VO_2_) and hydrogen peroxide (H_2_O_2_) emission as a function of PCoA concentration, in the presence of L-carnitine and malate, were performed. We found that PCoA concentration < 200 nmol/mg mito protein resulted in low H_2_O_2_ emission flux, increasing thereafter in Sham and T1DM GPs under both states 4 and 3 respiration with diabetic mitochondria releasing higher amounts of ROS. Respiratory uncoupling and ROS excess occurred at PCoA > 600 nmol/mg mito prot, in both control and diabetic animals. Also, for the first time, we show that an integrated two compartment mitochondrial model of β-oxidation of long-chain fatty acids and main energy-redox processes is able to simulate the relationship between VO_2_ and H_2_O_2_ emission as a function of lipid concentration. Model and experimental results indicate that PCoA oxidation and its concentration-dependent uncoupling effect, together with a partial lipid-dependent decrease in the rate of superoxide generation, modulate H_2_O_2_ emission as a function of VO_2_. Results indicate that keeping low levels of intracellular lipid is crucial for mitochondria and cells to maintain ROS within physiological levels compatible with signaling and reliable energy supply.

## Author summary

Lipids are main sources of energy for liver and cardiac and skeletal muscle. Mitochondria are the main site of lipid oxidation which, in the heart, supplies most of the energy required for its blood pumping function. Paradoxically, however, lipids over supply impair mitochondrial function leading to metabolic syndrome, insulin resistance and diabetes. In this context, scientific debate centers on the impact of lipids and mitochondrial function on diverse aspects of human health, nutrition and disease. To elucidate the underlying mechanisms of this issue, while accounting for both the fundamental role of lipids as energy source as well as their potential detrimental effects, we utilized a combined experimental and computational approach. Our mitochondrial computational model includes β-oxidation, the main route of lipid degradation, among other pathways that include oxygen radical generation and consumption. Studies were performed in heart mitochondria from type 1 diabetic and control guinea pigs. Model and experimental results show that, below a concentration threshold, lipids fueling proceeds without disrupting mitochondrial function; above threshold, lipids uncouple mitochondrial respiration triggering excess emission of oxidants while impairing antioxidant systems and the mitochondrial energy supply-demand response. These contributions are of direct use for interpreting and predicting functional impairments in metabolic disorders associated with increased circulating levels of lipids and metabolic alterations in their utilization, storage and intracellular signaling.

## Introduction

Fatty Acids (FAs) are main sources of cellular energy affecting mitochondrial energetics and redox balance. The lipid energy content becomes available from β-oxidation as reducing equivalents and acetyl CoA (AcCoA) of which the latter, after further processing in the tricarboxylic acid cycle, also supplies most of the energy as NADH and FADH_2_, which, in turn, fuel the buildup of the proton motive force for oxidative phosphorylation (OxPhos). Under physiological conditions, the non-esterified forms of FAs represent an important fuel supply in many tissues. However, persistent excess of FAs and accumulation of triacylglycerols in non-adipose tissues are associated with metabolic disorders like diabetes, hyperlipidemia and lipodystrophies [1,2].

Preserving the intracellular redox environment is crucial for vital functions such as division, differentiation, contractile work and survival, amongst many others [3,4,5,6,7,8,9,10,11]. Mitochondria are main drivers of intracellular redox [12,13,14,15,16], playing a central role in the development of diabetes and obesity complications [17,18,19,20,21]. Hearts from diabetic subjects are particularly prone to excess ROS because sympathetic hyper-activation and -glycemia are present in a large cohort of these patients [22,23]. These two conditions may alter cardiac and skeletal muscle redox conditions [5,6] endangering mitochondrial function [7,8]. Perturbations of cardiac mitochondrial energetics and increased mitochondrial ROS emission can account for tissue redox imbalance [8,11,12,13] and abnormal cardiac contractility leading to systolic and diastolic dysfunction in diabetic patients [17,18,19,20,21]. These abnormalities are common features in T1DM and type 2 diabetes mellitus (T2DM) patients [1,9,10] and they underlie diabetic cardiomyopathy, a major life-threatening complication that limits life quality and expectancy [3,19].

Although available evidence indicates the participation of oxidative stress in the etiology of T1DM, obesity-induced insulin resistance and T2DM [10,17,24,25,26], the role of dysfunctional β-oxidation *per se* as an underlying cause of metabolic disorder remains a topic of active research and debate [10]. Prevailing wisdom indicates that the myocardial shift from glucose to FA utilization occurring in diabetes may aggravate mitochondrial dysfunction, fueling contractile deficit [25,27]. Dysfunctional lipid metabolism in diabetes has been implicated in the development of cardiac impairment [28] and lipotoxicity resulting from accumulation of triacylglycerols and free FAs in the cytoplasm, which lead to the generation of apoptosis inducers such as diacylglycerol and ceramide [29]. In contrast, other studies have reported that FAs may actually benefit cardiac function in the course of metabolic syndrome [17,30,31]. In T1DM [32] and T2DM animal models [18,21] exhibiting impaired heart function when subjected to metabolic stress caused by hyperglycemia and elevated energy demand, it was shown that, unlike insulin, palmitate was able to rescue contractile function from the detrimental action of hyperglycemia. The beneficial effect of palmitate was concomitant with a higher content of reduced glutathione (GSH) and augmented mitochondrial ROS-scavenging capacity [18,21].

**Fig 1:**
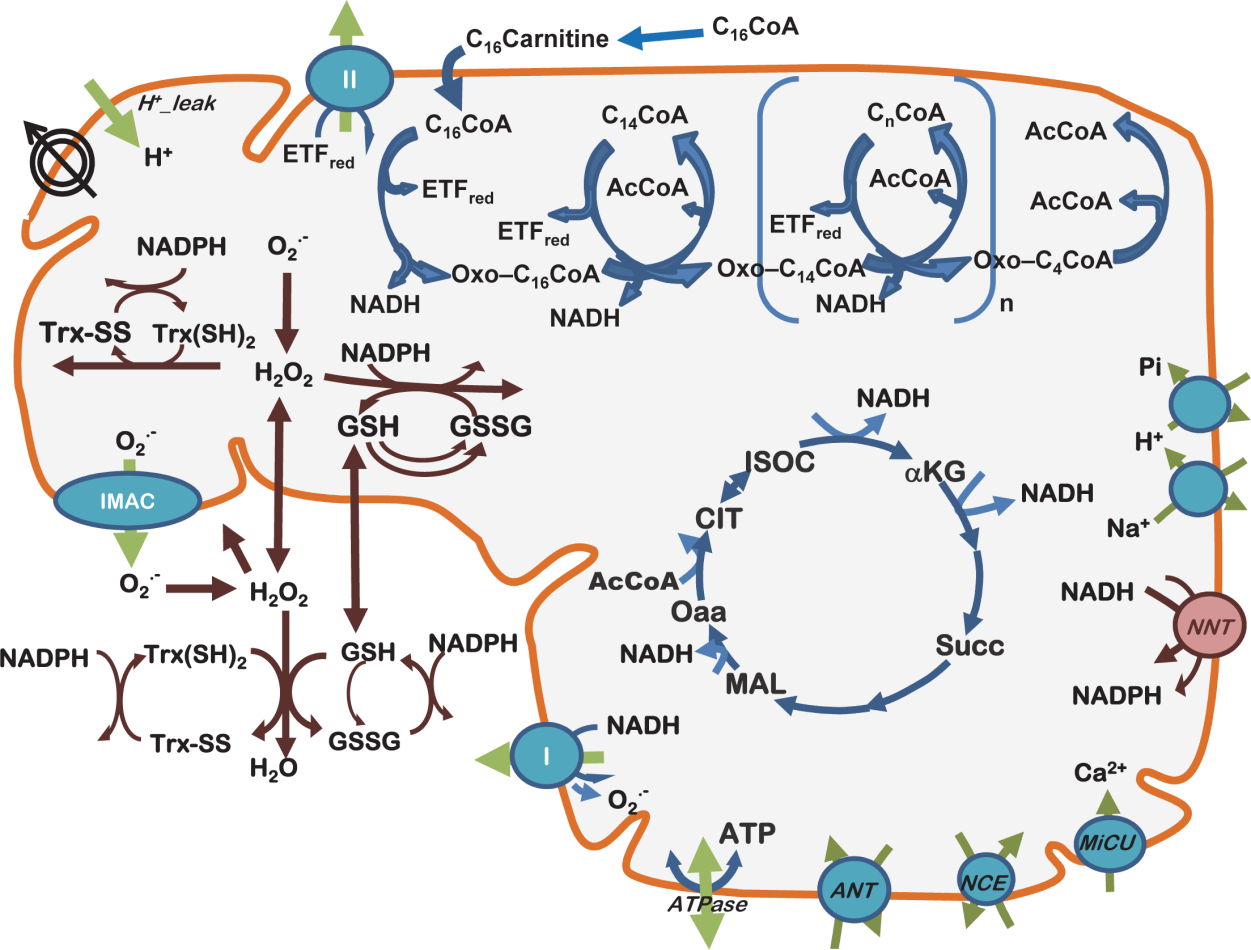
Scheme of the two-compartment mitochondrial model including β-oxidation and lipid transport. The present model encompasses mitochondrial energetic and redox processes, their interactions, and transport between compartments, in addition to energy metabolism, ion transport (H^+^, Ca^2+^, Na^+^, Pi) and β-oxidation (top). O_2_^.−^ is dismutated to H_2_O_2_ by matrix-localized superoxide dismutase (MnSOD) or transported to the extra-mitochondrial compartment, where it will be scavenged by Cu,ZnSOD. H_2_O_2_ can either diffuse from the matrix or be scavenged by the large capacity glutathione (GSH) and thioredoxin (Trx) systems, or by catalase (CAT) in the extra-mitochondrial compartment. The palmitate oxidation is linked to the TCA cycle via AcCoA and NADH. Also, NADH and FADH_2_ (bound to the electron transfer flavoprotein, ETF) are involved in the interaction between β-oxidation and the respiratory chain. The multiple regulatory interactions involved are not represented for simplicity. *Key to symbols*: Concentric circles with an arrow across (upper left of the scheme) represent the ΔΨ_m_. The scheme is modified from [15,63].

Together with peroxisomes, mitochondria represent the main subcellular compartments where lipid degradation occurs. Yet, the impact of dietary lipids on mitochondrial redox status and ROS emission, and their downstream effects on energetics are not fully elucidated. Thus, we investigated the basic mechanisms underlying the impact of lipid-precursor availability for β-oxidation on the energetic and redox responses from heart mitochondria of a previously described animal model of T1DM in STZ-treated GP that harbor glucose levels similar to those found in human T1DM [32,33]. More specifically, we analyzed how the relationship between mitochondrial respiration and ROS emission is altered as a function of PCoA in T1DM GPs and Sham controls. The experimental results are interpreted with the help of a two-compartment mitochondrial energetic-redox computational model [15] that includes β-oxidation [34] functionally linked to main redox couples and scavenging systems distributed in mitochondrial matrix and extra-matrix compartments, and transport between compartments of ROS species and GSH (Fig 1).

## Results

### Experimental dose-response behavior of mitochondrial respiration and ROS emission as a function of PCoA concentration under β-oxidation conditions

We quantified VO_2_ and H_2_O_2_ emission in isolated heart mitochondria from Sham and diabetic GPs under β-oxidation conditions with PCoA, in the presence of 0.5mM malate and 0.5mM L-carnitine, and in the absence (state 4) or presence (state 3) of 1mM ADP. As a caveat, Mal is needed to feed the TCA cycle to enable the efficient regeneration of Coenzyme A from acetyl CoA and cycling of β-oxidation [34].

**Fig 2:**
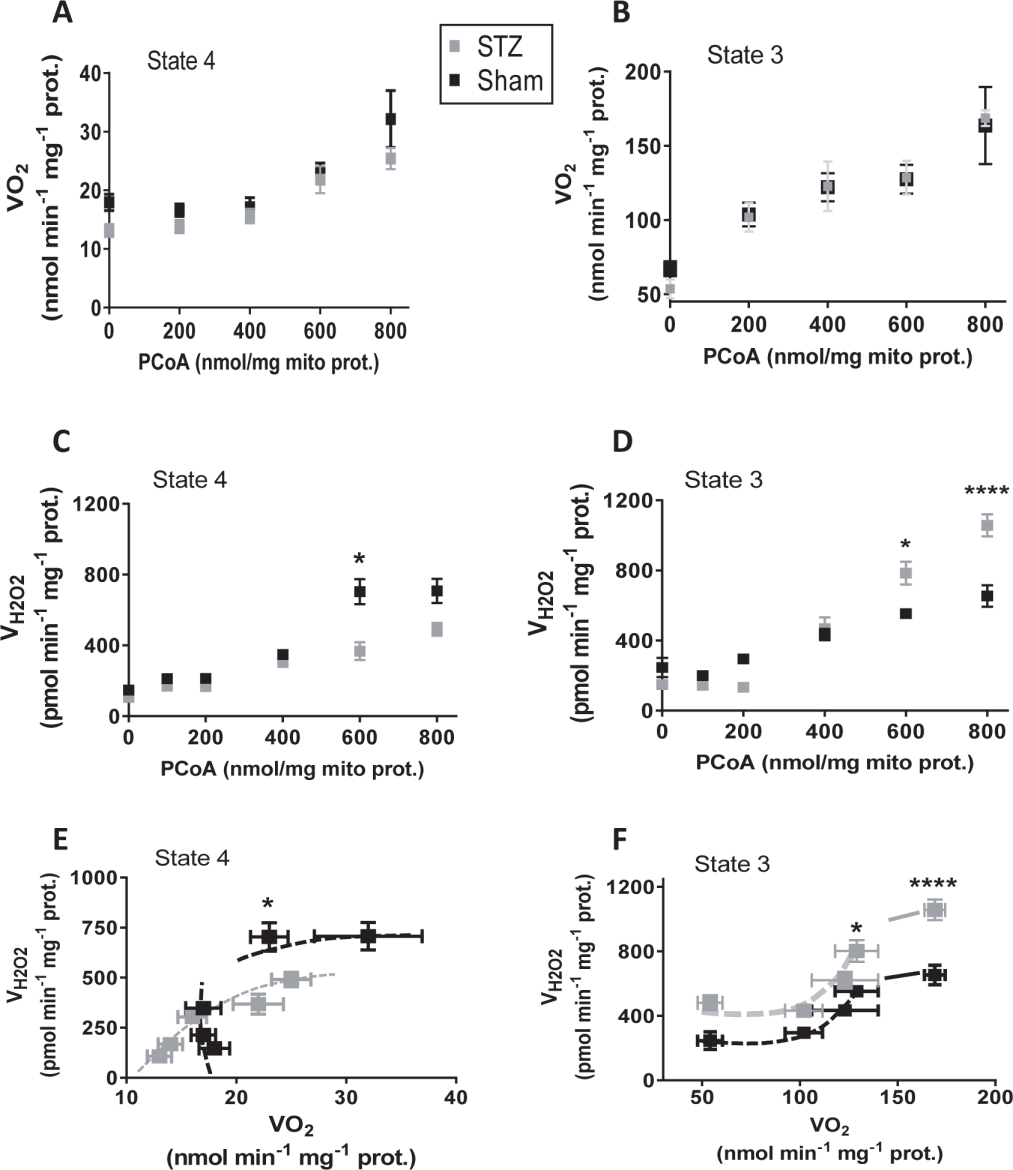
Respiratory and ROS emission fluxes from Sham and diabetic mitochondria as a function of PCoA concentration. Freshly isolated heart mitochondria from Sham and diabetic GPs were assayed for β-oxidation driven respiration and H_2_O_2_ emission as described in detail under Materials and Methods. Depicted are the specific rates of O_2_ consumption, VO_2_, (A, B) and H_2_O_2_ emission fluxes (C, D) determined under states 4 (no ADP; A, C) and 3 (+1mM ADP; B, D) respiration in Sham and STZ-treated (diabetic) mitochondria, respectively. The specific rates of O_2_ consumption and H_2_O_2_ emission, measured in parallel in the same mitochondrial preparations at different PCoA concentrations (panels A-D), were plotted against each other for Sham and diabetic under states 4 (E) and 3 (F) respiration. N = 12 technical repeats from 3 biological replicates (experiments/hearts) in each Sham or diabetic group. Raw traces of O_2_ consumption and H_2_O_2_ emission from representative experiments with Sham and diabetic mitochondria are shown in Fig C in S1 Text.

**Fig 3:**
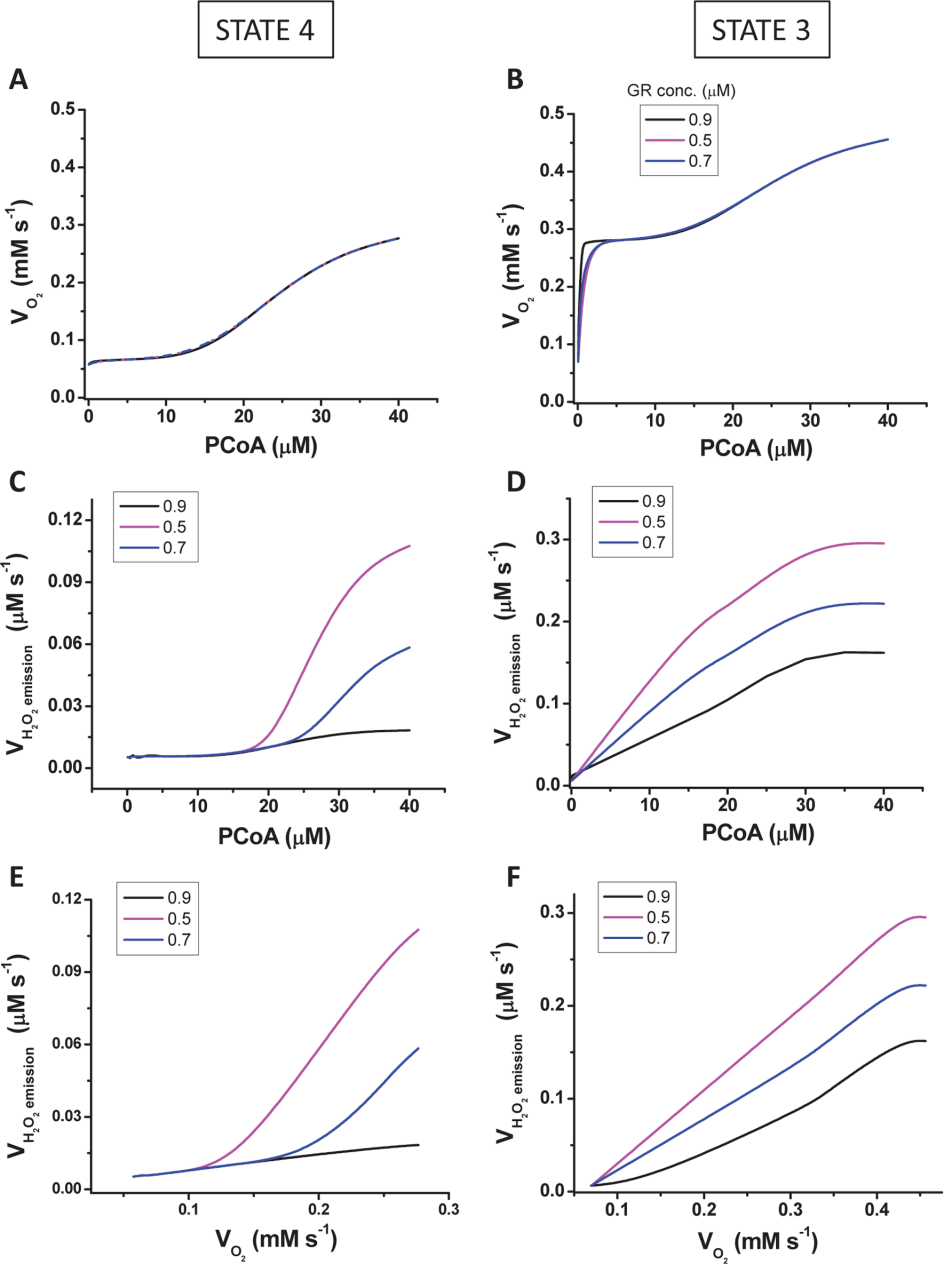
Model simulations of mitochondrial respiration and ROS emission fluxes from Sham and diabetic mitochondria vs. PCoA concentration. Steady state values of the rates of respiration (VO_2_) (A, B), H_2_O_2_ emission (V_H2O2_) (C, D) and their relationship, i.e. V_H2O2_ vs. VO_2_ (E, F) were obtained at the indicated levels of PCoA (in μM) under states 4 (A, C, E) and 3 (B, D, F) respiration. The plots display total VO_2_ (i.e., derived from NADH, succinate and FADH_2_ driven electron transport). The three curves represent simulations obtained at three different Vmax values of glutathione reductase (both intra- and extra-mitochondrial) as indicated in the legend of the plots. The model parameterization and initial conditions employed in the simulations are described in S1 Text together with the Matlab code for the full computational model.

Fig 2 depicts the results obtained in VO_2_ (Fig 2A and 2B) and H_2_O_2_ emission (Fig 2C and 2D) under states 4 and 3 respiration and as a function of PCoA concentration (0 to 800nmol PCoA/mg mito prot equivalent to 0 to 40μM PCoA: see Fig 3) in control (Sham) and diabetic (STZ) groups. State 3 respiration increased, attaining an apparent plateau level of ~125nmol O_2_/min/mg mito prot at 600nmol PCoA/mg mito prot in mitochondria from both Sham and diabetic GPs (Fig 2B). At amounts > 600nmol PCoA/mg mito prot VO_2_ further augmented an additional ~25% suggesting uncoupling of respiration (Fig 2B). State 4 respiration was stable at ~15nmol O_2_/min/mg mito prot up to 400nmol PCoA/mg mito prot, subsequently increasing with PCoA concentration (Fig 2A).

Mitochondrial ROS release remained approximately constant at ~ 150-200pmol H_2_O_2_/min/mg mito protein up to 200nmol PCoA/mg mito protein, in Sham and diabetic groups, and for both states 4 and 3 respiration (Fig 2C and 2D). Thereafter, a PCoA concentration-dependent increase in H_2_O_2_ emission occurs at PCoA > 200nmol/mg protein that in states 4 and 3 respiration plateaus in Sham at ~ 600pmol H_2_O_2_/min/mg mito protein (Fig 2C and 2D), In contrast, H_2_O_2_ emission from diabetic mitochondria under state 3 respiration increased almost 2-fold higher compared to Sham controls (Fig 2D), whereas in state 4 lower values than Sham were attained (Fig 2C).

The relationship between the rates of respiration and H_2_O_2_ emission in heart mitochondria from Sham and diabetic GPs is also shown in Fig 2. In state 3 respiration, the ROS efflux stays approximately constant in the VO_2_ range from 50 to 100 nmol O_2_/min/mg mito prot but ~2-fold higher in diabetic as compared to Sham (Fig 2F). At higher state 3 respiratory fluxes, ROS emission increases steadily as a function of VO_2_ up to ~ 130nmol O_2_/min/mg mito prot, and plateauing at VO_2_ > 150nmol O_2_/min/mg mito prot, although higher in diabetic than in Sham GPs (Fig 2F). At both ends of low and high respiration exhibited by mitochondria exposed to different PCoA concentrations (Fig 2A–2D), the H_2_O_2_ emission expressed as a percentage of the total O_2_ consumption flux [13] were for Sham/diabetic GP, respectively, 0.45%/0.89% and 0.38%/0.63% in state 3 (Fig 2F) and 0.82%/0.82% and 2.2%/1.96% in state 4 respiration (Fig 2E). Unlike in state 4 mitochondria from diabetic GPs, in which H_2_O_2_ emission augmented steadily as a function of VO_2_, in Sham ROS release remained independent from VO_2_ to increase only after a certain threshold of respiratory flux was overcome (Fig 2E).

Together, the experimental results obtained so far show that at PCoA < 200nmol/ mg prot, VO_2_ and H_2_O_2_ emission remained constant, while both increased as a function of the lipid precursor within the range 200–600nmol PCoA/mg mito prot, with overt (non-compensated) respiratory uncoupling and excess ROS emission happening at > 600nmol/ mg mito prot.

### Computational simulations and mechanistic insights into mitochondrial respiration and ROS emission under β-oxidation from PCoA

To help interpret the mechanisms underlying the observed increase in VO_2_ and ROS efflux from mitochondria, our computational model was utilized to simulate the experimental data under conditions mimicking those employed with isolated mitochondria. The simulations shown in Fig 3 reproduce the shape of the increase in VO_2_ as a function of PCoA concentration (0 to 40μM PCoA equivalent to 0 to 800nmol PCoA/mg mito prot: see Fig 2) observed in the experiments corresponding to states 4 (compare Fig 2A with Fig 3A) and 3 respiration (compare Fig 2B with Fig 3B). In state 4 respiration the model results reproduce the rise of V_H2O2_ at PCoA concentration above 20 μM while further showing that the extent of the increase in V_H2O2_ can be modulated by the scavenging capacity of mitochondria, i.e., achieving a higher V_H2O2_ at lower scavenging levels (simulated with different concentrations of glutathione reductase, GR; compare Fig 3C with Fig 2C). In state 3 respiration, model simulations are able to reproduce the rise and saturation of V_H2O2_ and, additionally, that the response can be modulated by the antioxidant capacity of mitochondria (compare Fig 3D with Fig 2D). Consequently, as suggested by the model simulations, the difference between Sham and diabetic mitochondrial H_2_O_2_ emission may be due to the lower scavenging capacity of the STZ-treated GPs (Fig 3).

Mechanistically speaking, our model simulations attribute a direct role to uncoupling of the mitochondrial inner membrane triggered by PCoA > 20μM (or > 400nmol PCoA/mg mito prot) as a main determinant of the modulation of the overall shape of the relationship of VO_2_ and V_H2O2_ *vs*. PCoA (Fig 2A–2D), along with the apparent sigmoidal behavior exhibited by these two fluxes under state 3 respiration when plotted together (Fig 2F). According to the model, the plateau of V_H2O2_ at high VO_2_, corresponding to PCoA > 30μM (or > 600nmol PCoA/mg mito prot; compare panels F from Figs 2 and 3) can be explained from a PCoA concentration-dependent uncoupling of mitochondria at high lipid concentration, reducing H_2_O_2_ emission through decreased ROS generation by the respiratory chain. On the other hand, overwhelming and/or causing impairment of the ROS scavenging systems can also modulate (up or down) the relationship between both respiratory and ROS fluxes (Figs 2 and 3).

### Experimental and computational evidence on the role of uncoupling and ROS scavenging systems in the redox-energetic impairment of mitochondria

**Fig 4:**
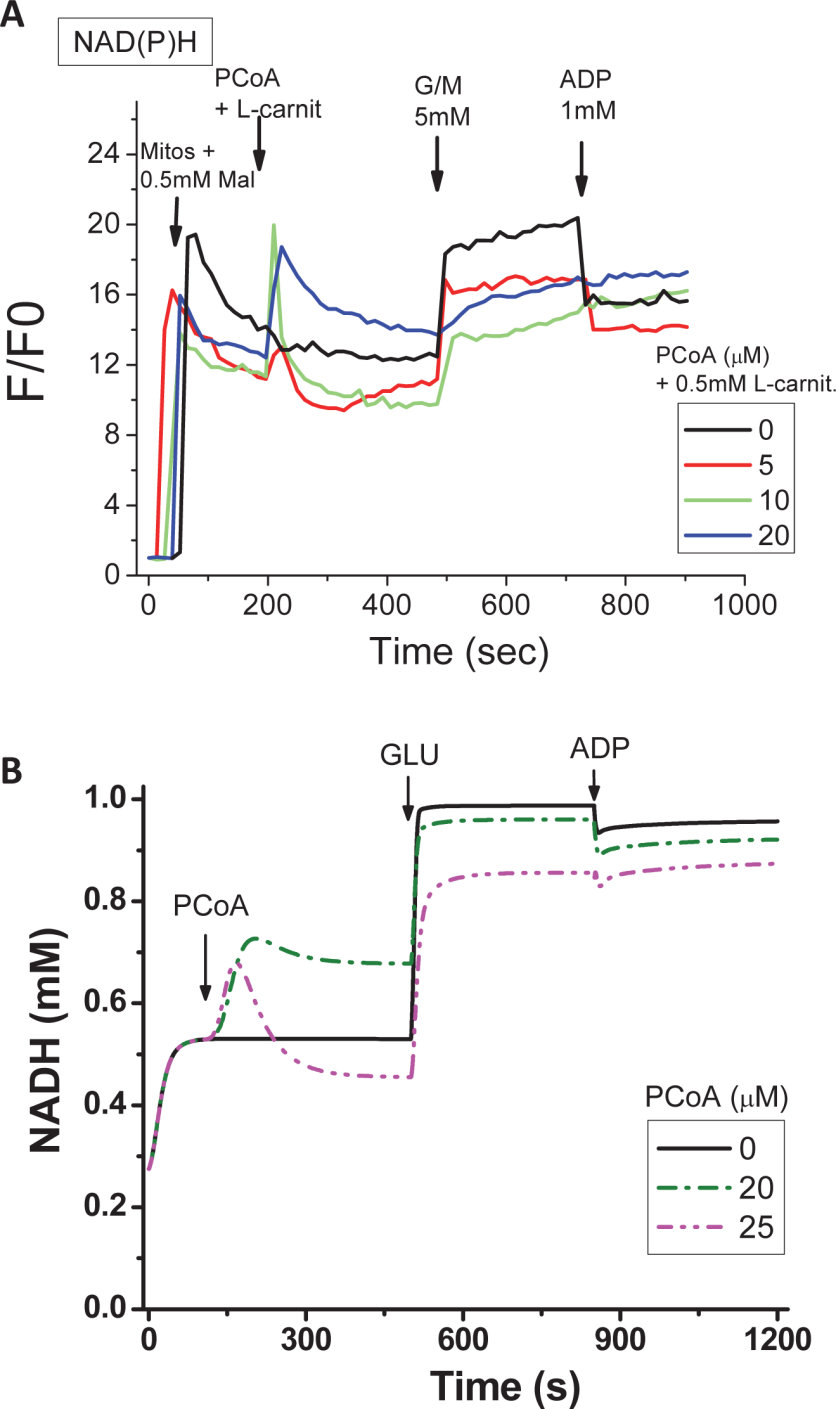
Experimental and computational redox behavior of mitochondrial NAD(P)H in the presence of PCoA-driven respiration, and its impact on mitochondrial function. Freshly isolated heart mitochondria from Sham or T1DM GPs were assayed in a fluorimeter in the presence of PCoA at the indicated concentrations, and as described in detail under Materials and Methods. (A) The first arrow in panel A indicates the addition time of mitochondria and 0.5mM malate (to enable the regeneration of the mitochondrial coenzyme A pool), after β-oxidation is triggered with the addition of both PCoA and L-carnitine (second arrow). Subsequent sequential additions of 5mM each of G/M and 1mM ADP were performed to test the state 4→3 transition. Depicted are the results from simultaneous monitoring of NAD(P)H in a typical experiment performed with Sham mitochondria but similar results were observed in mitochondria from diabetic GPs. (B) Model simulations of the transient, time-dependent, behavior of NADH upon successive addition of PCoA followed by glutamate and ADP to test the state 4→3 transition as indicated. The simulations condition mimic the experimental protocol in order to reproduce the experimental time courses depicted in panel A. All other model parameters are the same to those indicated in S1 Text.

To investigate the impact of lipid uncoupling on mitochondrial energetics, we performed experiments with isolated mitochondria consuming PCoA while monitoring NAD(P)H [35], and the results are depicted in Fig 4. As a measure of mitochondrial energetics, we monitored NAD(P)H levels during β-oxidation with PCoA 0–20μM (equivalent up to 400nmol PCoA/mg mito prot). Within the PCoA concentration range evaluated, mitochondria conserve the state 4→3 transition triggered by 5mM G/M followed by 1mM ADP, as a lipid-independent way to assess the energetic response [21,36], but start to show some impairment at 20μM PCoA, as can be judged from the response to ADP (Fig 4A). Computational simulations mimicking the experimental protocol show, firstly, that the experimentally determined initial NAD(P)H response to PCoA addition corresponds to the expected redox rise from β-oxidation while its subsequent decrease could be explained by PCoA consumption via β-oxidation (Fig 4B), as indicated by the extent of the NAD(P)H peak *vs*. PCoA (Fig 4A); secondly, that at PCoA > 20μM an uncoupling effect by the lipid becomes noticeable, leading to a diminished response to ADP during the state 4→3 transition (Fig 4A and 4B). The differences between experimental and model simulations data after PCoA, but before G/M, addition, can be explained by the fact that, unlike in the experiment, PCoA is “clamped” in the model at the indicated concentration. This explains that the more pronounced NADH oxidation observed in the model at 25μM compared to 20μM PCoA corresponds, to a certain extent, to the uncoupling effect of the lipid whereas in the experiments PCoA is consumed faster via β-oxidation thus the uncoupling effect is less prominent. Besides it is worth mentioning that the model could simulate the respiratory coupling ratio (RCR) observed experimentally to an acceptable approximation (~4 *vs*. 6, theoretical *vs*. experimental, respectively). As a caveat, while experimentally the RCR decreased from 6 to ~5 at 40μM PCoA (= 800nmol/mg mito prot) (Fig 2A and 2B), in the model it dropped from ~4 to ~2 at 10μM and >30μM PCoA, respectively. Thus, in the model the effect of uncoupling is higher than in the experiments.

**Fig 5:**
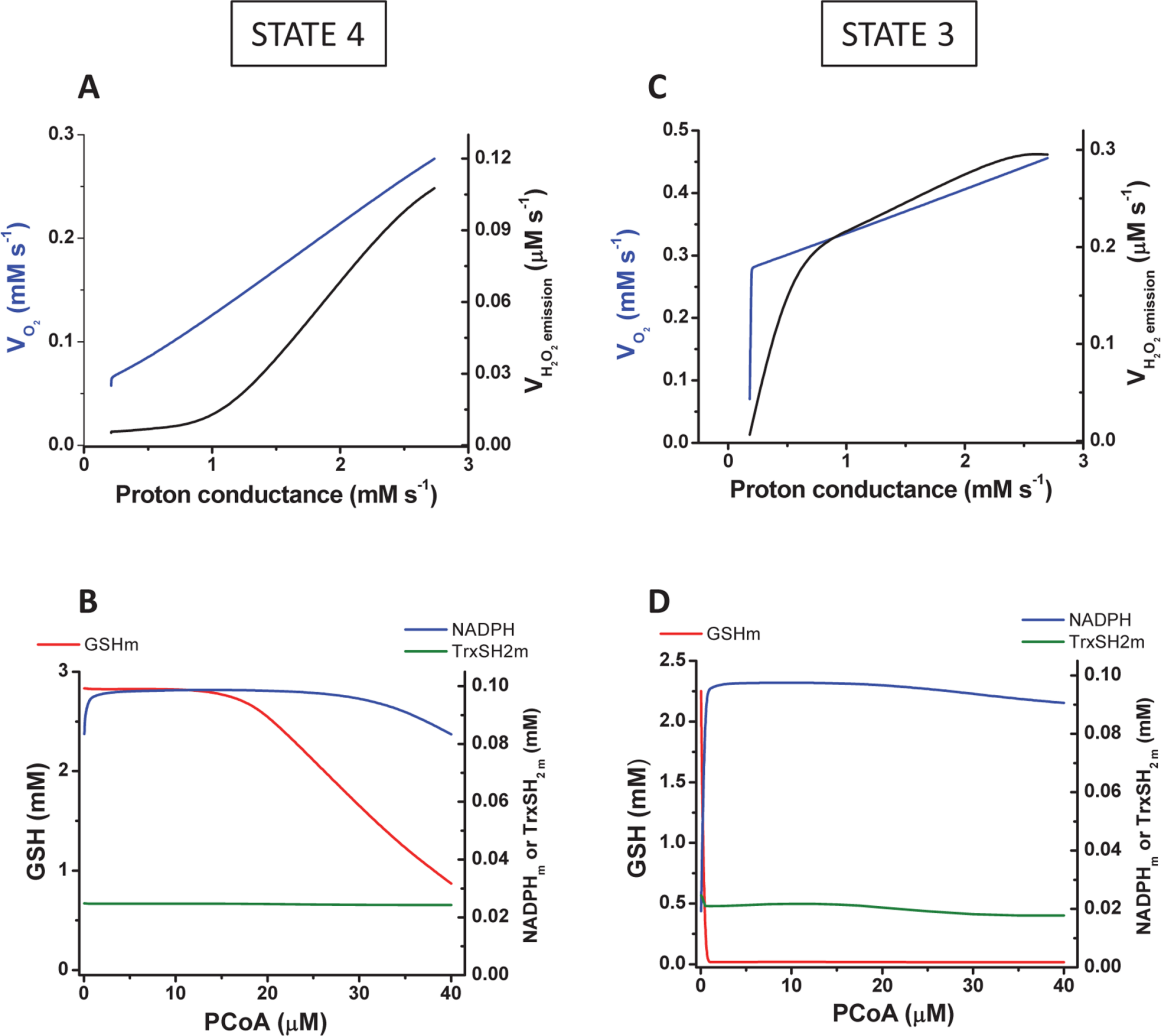
Modeling studies of mitochondrial respiration, H_2_O_2_ emission and ROS scavenging intermediates as a function of PCoA concentration and proton conductance. The steady state behavior of VO_2_ in the model was analyzed for the same range of PCoA as in Fig 3 mimicking those used in the experiments under states 4 (A) and 3 (C) respiration (blue traces) shown in Fig 2A and 2B. Also depicted in black traces are the rates of H_2_O_2_ emission corresponding to the same simulations. Panels B and D display the steady state concentration of main components of matrix antioxidant systems (GSH, Trx(SH)_2_, NADPH) under both states 4 (B) and 3 (D) respiration. The parameterization and initial conditions employed in the simulations are described in S1 Text together with the Matlab code for the full computational model.

Mechanistically, the PCoA uncoupling effect was taken into account by the model through an increase of the mitochondrial leak via the PCoA-dependent increase in proton conductance (Fig 5A and 5B). Under parametric conditions in which the model was able to reproduce the overall shape of H_2_O_2_ emission as a function of mitochondrial respiration (compare panel F from Figs 2 and 3), Fig 5 depicts changes in protein conductance and redox components of the glutathione and thioredoxin antioxidant systems as a function of PCoA, within the experimentally assayed concentration range, in both states 4 (Fig 5A and 5B) and 3 respiration (Fig 5C and 5D). At PCoA > 20μM, a significant increase in the mitochondrial H_2_O_2_ emission flux (V_H2O2_) happens in state 4 respiration (Fig 5A and 5B), together with a parallel decrease in mitochondrial glutathione (GSH_m_), accompanied by a slight decrease in the reduced pool of thioredoxin (Trx[SH]_2_), followed by NAD(P)H oxidation at higher PCoA concentration (Fig 5B). In state 3 respiration, V_H2O2_ describes a biphasic curve of increase as a function of proton conductance (Fig 5C), in which the initial phase occurs associated with an abrupt decrease in GSH_m_ whereas the second, smoother phase appears to be determined by oxidation of Trx[SH]_2_) and NAD(P)H at relatively higher PCoA concentrations (Fig 5D).

**Fig 6:**
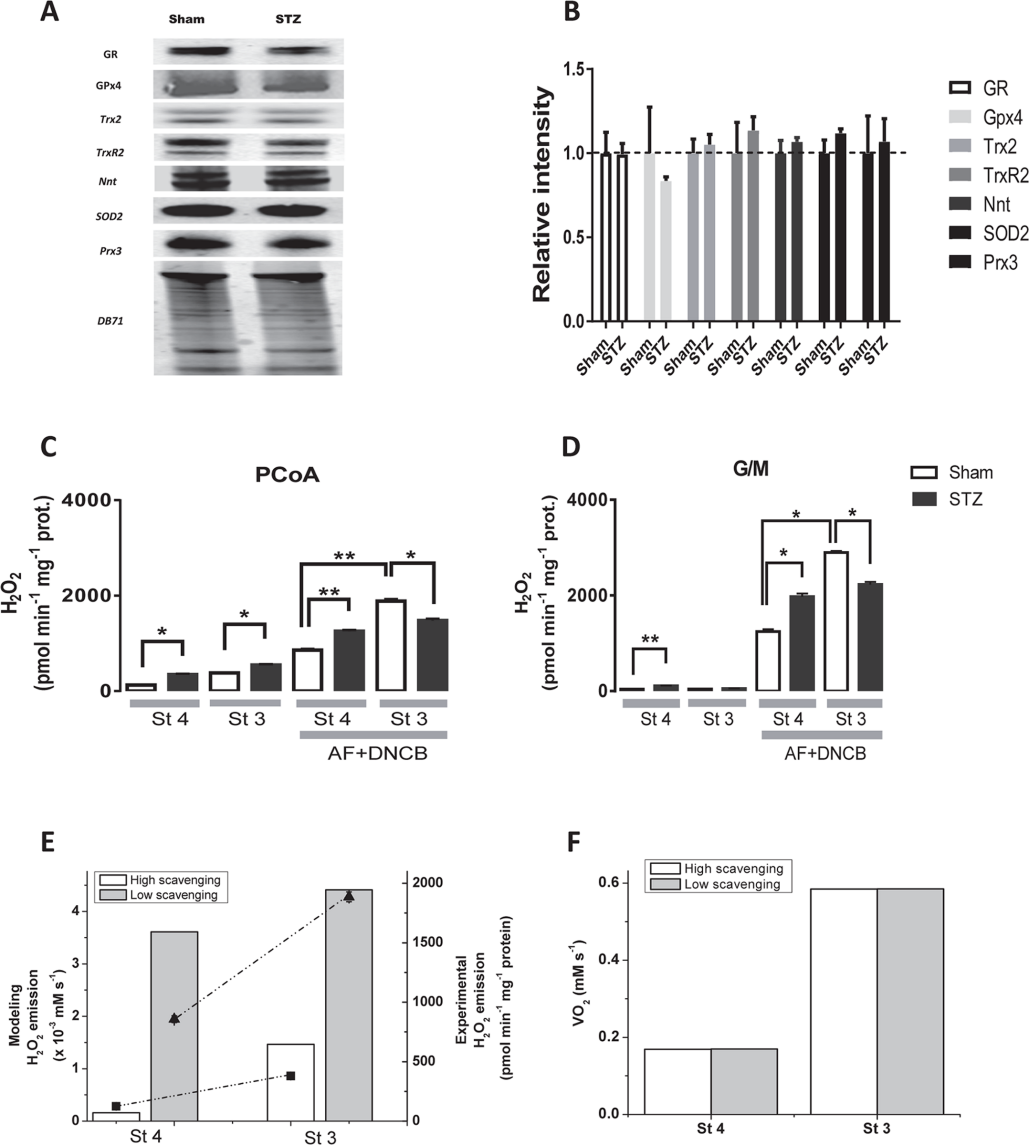
Mitochondrial antioxidant defense protein levels and ROS emission in the absence or the presence of inhibitors of the GSH and Trx antioxidant systems in Sham and STZ hearts. (A, B) Heart tissue from Sham or diabetic GPs was processed and the antioxidant proteins indicated were analyzed by Western Blot as described in Materials and Methods. Left panel shows representative Western Blot analysis of protein abundance. The bar plot on the right panel displays the statistical comparison between the results obtained with heart tissue from the two groups [n = 4, four experiments]. Protein was normalized to total protein abundance in a given lane based on Direct Blue 71 (DB71) staining of the membrane as described [16,21]. Key to symbols: GR, glutathione reductase; Gpx4, glutathione peroxidase 4; Trx2, thioredoxin 2; TrxR2, thioredoxin reductase 2; Nnt, nicotinamide nucleotide transhydrogenase; SOD2, superoxide dismutase 2; Prx3, peroxiredoxin 3. (C, D) Freshly isolated heart mitochondria (100μg mitochondrial protein) from Sham or STZ-treated GP were preincubated without or with 50nM auranofin (AF) plus 10μM 1-chloro-2,4 dinitrobenzene (DNCB) [13,16]. Monitoring of H_2_O_2_ was performed with an Amplex red assay during state 4 (with [A] 10μM PCoA/0.5mM malate/0.5mM L-carnitine or [B] 5mM each of G/M) and state 3 (+1 mM ADP) of mitochondrial respiration, using a wavelength scanning fluorometer (QuantaMaster; Photon Technology International, Inc.) [13,16] in the same assay medium utilized for high throughput measurements. The specific fluxes of H_2_O_2_ emission are shown for PCoA (C) and G/M (D) in the absence or the presence of AF + DNCB. (E, F) Depicted are the model simulated rates of ROS emission and O_2_ consumption upon inhibition of antioxidant defense activities, glutathione reductase (GR) and thioredoxin reductase (TrxR) to mimic the actions of the experimentally utilized inhibitors DNCB and auranofin, respectively (C, D). The plots display the steady state values of H_2_O_2_ emission (E) and VO_2_ (F) obtained at high (control: 100% GR and TrxR activities, empty bars) and low (15% and 22% of GR and TrxR activities, respectively, grey bars) scavenging capacity, under states 4 and 3 respiration as indicated. For comparative purpose, in panel E are shown the experimental values of Sham depicted in panel C, corresponding to the absence (filled squares) or presence (filled triangles) of inhibitors, under states 4 and 3 respiration as indicated. The simulations correspond to 40μM PCoA with 5x10^-4^mM and 0.1mM ADP in states 4 and 3, respectively, while the remaining parameters are described in S1 Text.

Together, the qualitative behavior of the experimental and model results converge in indicating the combined involvement of uncoupling and the ROS scavenging systems in the mitochondrial energetic-redox response to PCoA during β-oxidation. The explanation offered by the model, i.e., that the concomitant increase in respiratory and H_2_O_2_ emission fluxes elicited by PCoA concentration (> 20μM) under state 3 respiration is associated with interdependent actions of lipid-elicited uncoupling and overwhelming/impairment of the matrix GSH and thioredoxin (Trx) antioxidant systems (Fig 5), was further tested experimentally. We asked whether mitochondria from diabetic GPs possessed a different protein expression profile of the antioxidant systems as compared to Sham controls, or the lipid was eliciting enzymatic activity impairment leading to loss of antioxidant capacity. The protein expression level of main mitochondrial ROS scavenging systems by Western blot revealed no significant differences between seven components of the antioxidant systems in mitochondria from Sham and diabetic GPs (Fig 6A and 6B). A similar outcome was found between wild types and two different animals models of type 2 diabetes, *db/db* mice [21] and Zucker diabetic fatty rats [18]. Since these results pointed out differences in activity as responsible for the results observed, we analyzed two of the main branches of the antioxidant defenses, GSH and Trx systems. Their antioxidant capacity was estimated by quantitating H_2_O_2_ emission in the absence or in the presence of 1-chloro-2,4 dinitrobenzene (DNCB) and auranofin (AF), two specific inhibitors of GSH/Trx, respectively [13,16], when mitochondria from Sham or diabetic GPs were consuming PCoA/malate or glutamate and malate (G/M). Judging from the specific mitochondrial H_2_O_2_ emission when GSH/Trx are inactive (presence of AF+DNCB) or active (absence of inhibitors), with substrates PCoA/malate, the amount of ROS generated under state 4 respiration that was scavenged was 87% and 79% in Sham and diabetic, respectively, and 83% and 73% in state 3 respiration (Fig 6C). With G/M in state 4 respiration, the amount of ROS generated that was scavenged was 97% and 95% in Sham and STZ, respectively, whereas it represented 98% and 98% in state 3 respiration (Fig 6D). The results obtained indicate that in the presence of PCoA the mitochondrial GSH/Trx scavenging capacity was lower than in G/M, and more so in diabetic than in Sham mitochondria, suggesting that the lipid oxidation or intermediates from the β-oxidation pathway reduced the activity of the antioxidant systems evaluated, resulting in their being overwhelmed. Comparatively, and with the exception of state 3 respiration in the presence of AF+DNCB, mitochondria from diabetic GPs released significantly more ROS than controls, both with PCoA (Fig 6C) or G/M (Fig 6D). The lower ROS emission from diabetic as compared to Sham mitochondria, when both GSH/Trx systems were inhibited with AF+DNCB, unveils previously described deficits in the GP animal model [32] in both the electron flow through the respiratory chain (Complex II and IV) and the phosphorylation system, which may ultimately constrain ROS generation. Model simulations of the increase in mitochondrial H_2_O_2_ emission in response to inhibition of the glutathione reductase from the GSH/Trx system, were able to reproduce semi-quantitatively the experimental results in the presence of PCoA as substrate (compare panels C-E in Fig 6). Irrespective of the changes in ROS efflux, VO_2_ did not change at low or high antioxidant capacity (Fig 6F).

Taken together, experimental and modeling results enable us to conclude that, compared to Shams, the larger ROS emission exhibited by heart mitochondria from diabetic GPs is due to a lipid-dependent decrease in antioxidant activity (namely GSH and Trx), and an associated mitochondrial uncoupling. These results also indicate that PCoA modulates the relationship between respiration and ROS emission from mitochondria within the concentration range of 200-600nmol/mg mito prot, with respiratory uncoupling and energetic-redox impairment occurring at PCoA > 400nmol/mg mito prot.

## Discussion

The main contribution of the present work is to propose and validate some of the mechanisms involved in the beneficial and detrimental consequences of lipid oxidation on mitochondrial function. A combined experimental and computational approach was applied to assess the effects on redox and energy metabolism of varying levels of PCoA, the CoA-activated form of palmitate. As a tool for analyzing the complex functional impact of lipids, we used a comprehensive computational model of mitochondrial energy-redox and ionic processes that includes compartmentation of ROS scavenging systems and the supply of AcCoA from β-oxidation (Fig 1). This computational model was built on the basis of an extensively validated model of mitochondrial energetics and redox balance [15] integrated to a β-oxidation model, based on the one developed by van Eunen and colleagues [34]. Among the existing hypotheses regarding the effects of lipids on mitochondrial physiology which were tested by the present experimental-computational approach, three of them stand out as specific contributions from the present work: (i) delineation of the combined roles of lipid on OxPhos uncoupling and antioxidant systems, namely glutathione and thioredoxin, and their modulation of the relationship between mitochondrial respiration and H_2_O_2_ emission fluxes; (ii) the lipid-mediated impairment of the ROS generation and scavenging activity, and its quantitative impact on mitochondrial H_2_O_2_ emission under both states 3 and 4 respiration, and (iii) detection of a critical lipid concentration threshold, below which energetic and redox mitochondrial functions proceed in a controlled manner, but above which these functions can be derailed. These contributions are of direct use for interpreting as well as predicting functional impairments in metabolic disorders associated with increased circulating levels of lipids and metabolic alterations in their utilization and storage as well as in ROS-dependent intracellular signaling. In the liver, Van Eunen and coworkers [34] have proposed that, under FAs overload, competition between intermediate metabolites from β-oxidation can lead to metabolic disease.

Specifically, we characterized H_2_O_2_ emission during β-oxidation-driven respiration in mitochondria isolated from diabetic and control Sham GP hearts. Experimental and modeling studies confirmed that noticeably increased respiratory uncoupling, ROS emission and energetic impairment start to occur at PCoA > 400nmol/mg mito prot (equivalent to PCoA > 20μM), due to the action of this lipid precursor of β-oxidation on uncoupling. Model simulations (Figs 3–5) and experiments (Figs 2, 4 and 6) further showed that high lipid concentration is responsible both for uncoupled respiratory flux and enhanced H_2_O_2_ emission concomitantly with impairment of the state 4→3 transition, and for overwhelmed matrix GSH/Trx scavenging systems likely due to inhibition of enzyme activity rather than differential protein level of mitochondrial antioxidant systems (Fig 6). Broadly speaking, these results are agreement with a wealth of existing knowledge while expanding on the impact of lipid excess on the impairment of GSH/Trx ROS scavenging systems and mitochondrial redox-energetics, especially under diabetic conditions [27,37]. Additionally, the present data also show that, within a certain concentration range, lipids can fulfill their energetic role without either impairing mitochondrial energetics or eliciting excessive release of ROS.

### FAs, β-oxidation and mitochondrial function

Through β-oxidation, FAs are main metabolic fuels for heart and skeletal muscle function [38]. In the heart, two thirds of the cellular ATP is generated from FAs which provide reducing equivalents (NADH and FADH2) via mitochondrial β-oxidation. The higher energy delivered by the saturated FA palmitate in the form of reducing power (i.e., three times higher than from glucose in ATP/mol substrate), provides electrons to antioxidant systems and mitochondrial respiration [17,39]. It has been shown that energization of mitochondria by substrate oxidation increases the antioxidant potential of the thioredoxin system in the mitochondrial matrix where Trx(SH)_2_ rose in parallel with NAD(P)H and GSH as well as mitochondrial membrane potential (ΔΨ_m_) after glutamate/malate addition and remained high both in states 3 and 4 respiration [16,21].

The rate of β-oxidation is led by demand, implying that increased work rate and ATP demand drives faster OxPhos and tricarboxylic acid cycle activity [38]. Although existing evidence favors the idea that during T1DM the myocardial shift from glucose to FA utilization may aggravate mitochondrial and contractile dysfunctions [27,40], recent studies show that FAs may actually benefit cardiac function, at least acutely, in the course of metabolic syndrome. Cardiac myocytes from T1DM GPs exposed to high glucose and adrenergic stimulation with isoproterenol were not able to fully contract and relax, an effect that was found associated with mitochondrial oxidized redox status leading to impaired ATP synthesis along with altered Ca^2+^ handling and myocyte mechanical function [32]. In this T1DM GP animal model [32], as well as in *db*/*db* mice exposed to high glucose and *β*-adrenergic stimulation [21], and in the Zucker Diabetic Fatty rat, where hyperglycemia had a significant negative impact on contractility of heart trabeculae [18], palmitate was able to rescue contractile performance via higher antioxidant capacity of the GSH/Trx systems.

The pathogenesis of diabetes involves alterations in lipid oxidation by mitochondria. Inherited or acquired mitochondrial dysfunction may cause slow FA degradation driving the accumulation of intramyocellular lipids [41,42]. Also, mismatch between excess lipid supply with respect to demand may generate excess ROS [10]. Although acceleration of the β-oxidation flux could improve insulin sensitivity, disease may also ensue from inappropriately elevated β-oxidation flux in the absence of demand. Central to any of these possible situations is to determine the mechanisms through which mitochondria control ROS release as a function of lipid availability, and how this affects their energetic function. In this regard, the present work shows that, in the heart, mitochondria can increase their ROS release as a function of the rate of β-oxidation dependent respiration, but also that impairment of mitochondrial energetics-redox function would only start to happen after a certain threshold of PCoA concentration (> 400 nmol/min/mg prot) is crossed, triggering, for example, progressive uncoupled respiration and ROS emission in both states 4 and 3 respiration in Sham and diabetic mitochondria (Figs 2 and 3) and impairment of the state 4→3 transition (Fig 4). Enhanced H_2_O_2_ emission was caused by concomitant OxPhos uncoupling with decreased activity of matrix GSH/Trx ROS scavenging systems according to experimental (Fig 6) and modeling evidence (Fig 5). In agreement with previous reports [17,18,21,32,43,44,45], these results support the notion that in the diabetic heart the antioxidant capacity is lower, thus explaining, at least in part, the increased levels of oxidative stress observed.

Besides their metabolic role in energy provision, long-chain FAs affect cellular membranes and enzyme catalysis [46]. Non-esterified and esterified FAs interfere with mitochondrial OxPhos *in vitro* [47] acting as weak uncouplers [48] by increasing state 4 respiration [49,50]. Under reverse electron transport, FAs dramatically decrease mitochondrial ROS generation by an as yet unknown mechanism [51]. In contrast, the relatively low ROS emission by mitochondria under forward electron transport is significantly increased in the presence of FA [48,51]. In the present work, we show that the latter is likely true at relatively high concentrations of lipid precursor whereas at relatively lower concentrations (≤ 400nmol PCoA/mg mito prot) H_2_O_2_ emission stays constant and low, although higher in diabetic as compared to control mitochondria (Fig 2).

### Lipids and mitochondrial redox-energetic balance under normal and diabetic conditions

Mitochondria modulate both the release as well as scavenging of H_2_O_2_ from the cytoplasm thus playing a key role in cellular redox conditions and redox-dependent signaling, vital for normal cell function [17,52,53]. Using targeted viral gene transfer vectors expressing redox-sensitive GFP fused to sensor domains to measure H_2_O_2_ or oxidized glutathione in H9c2 cells, and selective knockdown (by 50%-90%) or overexpression of antioxidant enzymes, Dey and colleagues [14] showed that ROS scavenging by mitochondria significantly contributes to cytoplasmic ROS handling. Knockdown of the cytosolic antioxidant enzymes had no statistically significant effect on mitochondrial matrix H_2_O_2_, in agreement with the idea that the mitochondrial scavenger reserve capacity was high enough to buffer H_2_O_2_ diffusing into the matrix even when the cytoplasmic system was impaired [14].

Keeping a proper cellular/mitochondrial redox environment is vital for optimal excitation-contraction (EC) coupling as well as energy supply in the heart [53,54,55]. Intracellular redox balance affects Ca^2+^ handling by functionally stabilizing a wide range of proteins implicated in EC coupling [30] including the sarcoplasmic reticulum (SR) Ca^2+^ release channels, the SR Ca^2+^ pumps, and the sarcolemmal Na^+^/Ca^2+^ exchanger [56].

Consistent with the concept of a prominent role of lipids on governing the intracellular redox status, it was shown that palmitate determines a transition from oxidized-to-reduced redox state coupled to a marked GSH rise that abated ROS levels drastically in cardiomyocytes from T1DM and T2DM hearts. As a consequence of its favorable effect on cellular redox balance, palmitate significantly improved contractile performance in cardiomyocytes from STZ-treated GPs [32], *db/db* mice [21] and heart trabeculae from Zucker rats [18].

### Lipid droplets (LDs) and the dynamic balance of lipid storage and utilization

The findings described herein suggest that keeping the intracellular levels of FAs low is critical to avoid detrimental oxidative stress. Under lipid surplus, development of tissue lipotoxicity and dysfunction are linked to alterations in LD biogenesis and regulation of hydrolysis of triacylglycerols [57]. In pathologic states lipotoxicity may occur over time [29], despite triacylglycerol accumulation, when either the cellular capacity for triacylglycerol (TAG) storage is exceeded or when triglyceride pools are hydrolyzed, resulting in increased cellular free FA levels. Thus, the duration and extent of lipid overload may determine if a cell is protected or damaged.

Lipid storage and utilization appears to be a tightly regulated cellular process (reviewed in [39]). Perilipins are involved in modulation of LD storage-utilization dynamics [57]. Reduced expression of perilipins may promote both lipolysis and fat oxidation, resulting in reduced intracellular TAG and adipose mass whereas excessive lypolysis and defective lipid storage may promote insulin resistance and impaired cardiac function through chronic mitochondrial FA overload. As a matter of fact, excessive triacylglycerol catabolism by perilipin5-deficient hearts is paralleled by increased FA oxidation and enhanced ROS levels leading to age-dependent decline in heart function. Consequently, uncontrolled lipolysis and defective lipid storage may impair cardiac function through chronic mitochondrial FA overload [58,59].

### Concluding remarks

Proper mitochondrial function is needed to sustain energy supply reliably while releasing ROS levels compatible with signaling. However, lipids in excess can derail both of these critical functions. In keeping with the results reported herein, cytoplasmic mechanisms for “sequestering” FAs (and those from lipotoxic intermediates) to keep their concentration low become relevant. Metabolic channeling of lipid transport and β-oxidation, involving direct delivery into mitochondria, may represent a reliable and efficient way to ensure energy supply and redox control. Such a mechanism could avoid exceeding the limit of lipid storage capacity and help in hindering lipotoxicity, which is relevant under heavy influx of FAs as happens in skeletal muscle or heart in matching energy supply with demand when subjected to high workload.

## Materials and methods

All procedures on guinea pigs to render them diabetic were approved by the Animal Care and Use Committee of Hilltop Lab Animals, Inc., and adhere to NIH public health service guidelines. For mitochondrial isolation, GPs were heparinized (500 IU) and euthanized with sodium pentobarbital (180 mg/kg intraperitoneal), following the requirements of the Institutional Animal Care/Use Committee at JHU, adherent to NIH guidelines.

### Diabetic guinea pig

GPs were rendered diabetic by Hilltop Lab Animals, Inc. (Scottsdale, PA), following the procedure that we described previously [32]. Briefly, male GPs (200-250g) were made diabetic by a single intra-peritoneal injection of buffered streptozotocin (*STZ group*, 80 mg/kg in citrate buffer pH 4.5). Littermate animals received an equivalent volume of vehicle (citrate buffer pH 4.5) (*Sham*). According to our protocol, elsewhere described [32], Sham and STZ animals were utilized after 4 weeks of STZ administration, the minimum time needed to observe the T1DM phenotype.

Diabetic GPs had 36% higher levels of glucose in blood (in mg/dl±S.E.M: Sham 153±3.2 vs. STZ 208±6.5; p<0.001, i.e., from ~8 to ~12mM glucose, n = 26 and n = 24, respectively). No significant differences in body weight between the two groups of animals were detected [32].

### Mitochondrial isolation

Procedures for the isolation and handling of mitochondria from guinea pig hearts were performed as previously described [12,35].

### High throughput assay of mitochondrial function

High-throughput–automated 96-well microplate reader analyses of respiration (XF96 extracellular flux analyzer; Seahorse Bioscience) [13], and ROS (H_2_O_2_) emission (Flex Station 3, Molecular Devices), were performed in parallel in freshly isolated mitochondria from GP heart.

The rate of O_2_ consumption, VO_2_, was evaluated in mitochondria (using the equivalent of 10μg of mitochondrial protein) under β-oxidation fueled conditions in the presence of PCoA in a dose-response manner (5–40μM, corresponding to 100–800 nmol PCoA/mg. mito prot), 0.5mM malate and 0.5mM L-carnitine, in a medium (buffer B, 200μl final assay volume) containing (in mM): 137 KCl, 2 KH_2_PO4, 0.5 EGTA, 2.5 MgCl_2_, and 20 HEPES at pH 7.2 and 37°C in presence of 0.2% fatty acid–free BSA [13].

The VO_2_ corresponding to states 4 and 3 respiration was determined before and after addition of 1mM ADP, respectively. Respiratory Control Ratios (state3/state4) of 5 or higher were obtained. The experimental rates are expressed in pmol min^-1^ mg^-1^ mitochondrial protein to enable comparison with data in the literature. However, modeling results are expressed in mM s^-1^ for V_O2_ and μM s^-1^ for V_H2O2_. A conversion factor of 1 μmol min^-1^ mg^-1^ protein = 16.67 mM s^-1^ relates both flux units based on a mitochondrial volume of 1 μl per mg of mitochondrial protein.

The day before the experiment, 120μl polyethylenimine (1:15000 dilution in buffer B of a 50% solution of polyethylenimine) were added to the wells of the XF96 plate and incubated overnight at 37°C. For comparison purpose, internal controls were run in the absence of β-oxidation with NADH-linked substrates (G/M, 5/5 mM each). Before the experiment, the solution of polyethylenimine was removed. After transfer of appropriate amounts of mitochondrial suspension into each well (10μg of mitochondrial protein), the microplate was centrifuged at 3,000 x *g* for 7 min at 4°C using a swinging bucket rotor (S5700; Beckman Coulter). To avoid temperature inhomogeneity effects, the plate was incubated at 37°C for 20 min before starting the assay in the Seahorse Bioscience equipment.

Using mitochondria from the same preparation (10μg mitochondrial protein), parallel fluorescence measurements of H_2_O_2_ emission with the Amplex Red kit (Invitrogen) (λ_exc_ = 530 nm and λ_em_ = 590 nm) were performed with a Flex station, under the same aforementioned substrate and buffer conditions, with the exception that BSA was not added and the plate wells were not coated with polyethylenimine. The H_2_O_2_ emissions corresponding to states 4 and 3 respiration were quantified before and after addition of 1mM ADP, respectively. The specific rate of H_2_O_2_ emission was determined in each well using an internal standard H_2_O_2_ solution, and calculated from the slopes of the normalized Amplex Red signal with respect to initial fluorescence (F0), before the addition of substrate. This procedure automatically discounts any drift of the baseline due to effects not specifically produced by the corresponding treatment [12,13].

Both VO_2_ and ROS emission measurements were performed in the linear range of detection with respect to the amount of mitochondrial protein utilized. Examples of raw data from O_2_ consumption rate and H_2_O_2_ emission measurements are displayed in Fig C in S1 Text.

Mitochondrial protein was determined using the bicinchoninic acid method protein assay kit (Thermo Fisher Scientific).

### Control experiments

Since the experiments reported herein are performed in aqueous media to avoid introducing additional hydrophobic chemicals (i.e., diluent) other than the lipid itself and the natural substrates of mitochondrial respiration, we chose PCoA because its critical micellar concentration (CMC) is in the range of 40–60μM at 23^°^C, which also depends upon ionic composition and pH [60,61]. In our mitochondrial assay buffered medium, at 37°C and in the absence of mitochondria, PCoA starts to form micelles at 40μM concentration, as checked by fluorometry using 90^o^ light scattering. To rule out unspecific surfactant effects elicited by PCoA within the concentration range utilized (up to 40μM), we measured mitochondrial respiration in the absence of malate (needed for feeding the tricarboxylic acid [TCA] cycle to enable β-oxidation to proceed). Under these conditions, VO_2_ with PCoA was very low, up to 3 and 7nmol O_2_.min^-1^.mg^-1^ prot in states 4 and 3, respectively. Comparatively, under the same conditions but in the presence of malate, respiration with PCoA was up to 22 and 125nmol O_2_. min^-1^. mg^-1^ prot in states 4 and 3, respectively. Importantly, as direct evidence that the mitochondria are coupled, at all PCoA concentrations there are clear differences between states 3 and 4 respiration (Fig 2A and 2B and Fig C in S1 Text), i.e., in uncoupled mitochondria states 4 and 3 respiration would be similar. Together, these controls rule out uncoupling due to unspecific permeabilization of mitochondrial membranes by PCoA.

With the aim of both controlling for putative binding effects of PCoA to albumin and its impact on mitochondrial H_2_O_2_ emission, and the reproducibility of the PCoA dose-response, we performed experiments in two different experimental setups (high throughput Flex station plate reader and low throughput fluorometric monitoring), and with the same freshly prepared mitochondria, in the absence or presence of 0.2% free FA BSA. These controls show that the results are reproducible, independently from the experimental set up, and that the results are not significantly different in the absence or presence of BSA in states 4 and 3 respiration (Fig D in S1 Text). We also present controls of mitochondrial respiration with 5mM G/M, in the absence or presence of 0.2% free FA bovine serum albumin, conducted in Seahorse XF96 equipment, showing that VO_2_ is not significantly affected by BSA.

### Other bioenergetic variables

NAD(P)H, and H_2_O_2_ emission with Amplex Red, were determined as previously described [12,13,35], and monitored simultaneously with a wavelength scanning fluorimeter (QuantaMaster; Photon Technology International, Inc.) using the same above mentioned medium for measuring respiration and a multidye program for simultaneous online monitoring of different fluorescent probes.

### Abundance of mitochondrial antioxidant defense proteins in Sham and STZ hearts

Heart tissue from Sham or STZ-treated GPs were homogenized using a polytron homogenizer into 5 volumes/weight of extraction buffer (50 mM Bis-Tris (pH 6.4), 2% SDS with protease inhibitor cocktail (EDTA Free, Roche). Protein concentration was determined using bicinchoninic acid [BCA] method protein assay kit (Thermo Fisher Scientific). Equal protein loads of extract were run on 4–12% Acrylamide Bis-Tris Gels (Life Technologies). Gels were transferred (using Biorad wet transfer apparatus) to nitrocellulose membranes with Tris/Glycine buffer and membranes blocked with Odyssey blocking reagent (Li-Cor Biosci.) in TBS buffer. Membranes were then probed with primary antibodies raised to the following antioxidant proteins: Thioredoxin 2 (Trx2, Rabbit Polyclonal, Abfrontiers), Thioredoxin Reductase 2 (TrxR2, Rabbit polyclonal, Abfrontiers), Glutatione Reductase (GR, Rabbit polyclonal, Ab Frontiers); Superoxide Dismutase 2 (SOD2, Rabbit Polyclonal, Santa Cruz Biotechnol.), Nicotinamide nucleotide transhydrogenase (NNT, Rabbit polyclonal, Aviva Systems Biology), Glutathione Peroxidase 4 (Gpx4, Rabbit polyclonal, Abcam), Peroxiredoxin 3 (Prx3, Rabbit polyclonal, Abfrontiers). Fluorescent secondary antibodies labeled with either IRDye 800CW or IRDye 680RD was used to visualize protein bands utilizing an Odyssey Infrared Scanner (Li-Cor Biosci.) and bands quantitated using Odyssey software.

### Materials

L-carnitine and palmitate were from Sigma, and palmitoyl Coenzyme A ammonium salt from Avanti Polar Lipids, Inc.

### Statistical analysis

Data were analyzed with the software GraphPad Prism [Ver. 6; San Diego, CA] or MicroCal Origin. Significance of the difference between treatments was evaluated with one-way ANOVA using Tukey's multiple comparison test, or with a *t* test [small samples, paired *t* test with two tail *p* values] and the results presented as mean±SEM [95% confidence interval].

## Model description

The scheme of the two-compartment mitochondrial energy-redox model including the β-oxidation pathway is depicted in Fig 1. The model is based on our previous two-compartment model of mitochondrial energetics [15], encompassing the use of AcCoA in the TCA cycle and the provision of glutamate that replenish TCA cycle intermediates (Fig 1). Also included in the model are pH regulation, ion dynamics [36], and main scavenging systems—glutathione [GSH], thioredoxin [Trx], superoxide dismutase [SOD], catalase—distributed in mitochondrial matrix and extra-matrix compartments, four main redox couples (NADH/NAD^+^, NADPH/NADP^+^, GSH/GSSG, Trx(SH)_2_/TrxSS), and transport between compartments of ROS species (superoxide, O_2_^.-^, hydrogen peroxide, H_2_O_2_), and GSH [15]. In the present work the model takes into account the AcCoA supply from β-oxidation as described in the following section.

### Modeling the β-oxidation pathway

The present model (Fig 1) accounts for the β-oxidation pathway from PCoA, which was modeled based on van Eunen et al. [34]. The model formulation considers the transport of PCoA from the cytoplasmic to the mitochondrial matrix via carnitine palmitoyltransferase I (CPT1), carnitine acylcarnitine translocase (CACT) and carnitine palmitoyltransferase II (CPT2) (Eqs. S1-S3 in S1 Text). As a caveat, our formulation differs from that of van Eunen and colleagues, since the only substrates of CPT2 considered are PCoA and palmitoyl carnitine. Thus, the competition of CPT2 and CPT1 through the various acyl-carnitine and acyl-CoA species was not taken into consideration in our β-oxidation model (section 1 in S1 Text).

The β-oxidation model describes the catabolism of PCoA through the recursive action of four enzymes: very long-, long-, middle- and short-chain fatty acyl-CoA dehydrogenases, catalyzing consecutive steps in cycles, where in each of seven cycles 2-carbon units (i.e., AcCoA) are released. β-oxidation reactions occur coupled to the reduction of either flavin adenine dinucleotide (FAD) in the steps catalyzed by the fatty acyl-CoA dehydrogenases, or NAD^+^ by β-hydroxy-acyl CoA dehydrogenases (equations S4-S22 and S31-38, respectively, in S1 Text). A more detailed description of the β-oxidation model equations and parameters can be found in S1 Text.

The coupling between β-oxidation, the TCA cycle and mitochondrial electron transport chain is accomplished through NADH, AcCoA and FADH_2_; the latter reduces the electron transferring protein FAD that, in turn, donates electrons to ubiquinone in the respiratory chain [62].

The role of PCoA in the present model can be both as a substrate—providing AcCoA and reduction equivalents to feed the TCA cycle and the respiratory chain through the electron carriers NADH and FADH_2_—and as an uncoupler at high concentration (above 200nmol/mg mito prot). The PCoA-mediated uncoupling is modeled as an increase in the proton conductance, g_H_ (Eq. S137 in S1 Text) as a function of the cytoplasmic PCoA concentration to the fourth power. The need for a fourth power dependence can be attributed to the system approaching the critical micellar concentration (CMC) and more molecules of PCoA being incorporated into the mitochondrial membrane, altering its permeability. A more detailed description of the β-oxidation model equations and parameters can be found in S1 Text.

Model simulations were run with a code written in MATLAB (The Mathworks, Natick, MA) using the ODEs15 integrator. In S1 Text, the system of ordinary differential equations (ODEs) (section 2 Appendix) and the Matlab code for the full computational model as well as parameters (Tables A-M), and initial conditions (Table N) are listed. Results reported correspond to steady state behavior, when the relative time derivative of each variable is ˂ 1.10^−10^ sec^-1^.

## Supporting information

S1 Text Supporting information comprising a brief, but full, model description, all equations, parameters and initial values of state variables used in the model simulations. The Matlab code for the full computational model is also included at the end of S1 Text.
(PDF)
