# Supplementary material for: Mitochondrial respiration and ROS emission during β-oxidation in the heart: An experimental-computational study
Source: PLoS Comput Biol. 2017 Jun 9;13(6):e1005588. doi: 10.1371/journal.pcbi.1005588 (PMC5482492; doi:10.1371/journal.pcbi.1005588)
Supplement: S1 Text — The Matlab code for the full computational model is also included at the end of S1 Text. (PDF) [file pcbi.1005588.s001.pdf]

Mitochondrial respiration and ROS emission during β-oxidation in the heart

An experimental-computational study

Sonia Cortassa, Steven J. Sollott and Miguel A. Aon

From the Laboratory of Cardiovascular Science, National Institute on Aging, National Institutes of Health, Baltimore, MD 21224

S1 Text

1. Appendix. Rate equations and β-oxidation model behavior

In the present work, the model of β-oxidation is based on a modified version of the model formulated by van Eunen and colleagues [1]. Since we were specifically interested in describing the catabolism of palmitoyl-CoA (PCoA), we neglected the conversion of other Acyl-CoA derivatives into the Acyl-carnitine intermediates and their transport outside the mitochondrion taken into account by van Eunen and colleagues. As a result, only $C16CoA_m$ (PCoA) and $C16Carn_m$ (mitochondrial palmitoyl carnitine) were considered substrates of carnitine palmitoyl transferase2 (CPT2) activity. Nevertheless, our model still accounts for the competition between substrates for the dehydrogenation steps since the fatty acyl-CoA dehydrogenases will exhibit affinity for various Acyl-CoA and Acyl-enoyl CoA intermediates instead of having different pools of enzymes catalyzing the same step.

Figure A displays a simulation of the isolated β-oxidation model, in which a time course of this pathway's intermediates is shown after a pulse of the lipid precursor PCoA in the extra-mitochondrial compartment. The main panel shows the time-dependent changes in the level of each of the Acyl-CoA intermediates, similar to those shown by van Eunen and colleagues for the Acyl Carnitine derivatives, although some differences in the relative timing of the peaks in each of the species are apparent. Although a direct comparison between both models is not strictly fair because ours, with the exception of palmitate, does not produce Acyl Carnitine derivatives for any other fatty acid metabolite, still the relative order of appearance of the different intermediates is the expected one (Fig A, inset), but with shorter delays compared to the Acyl-Carnitine derivatives from the van Eunen model [1], likely due to the lack of the conversion step into the carnitine derivatives in the present model.

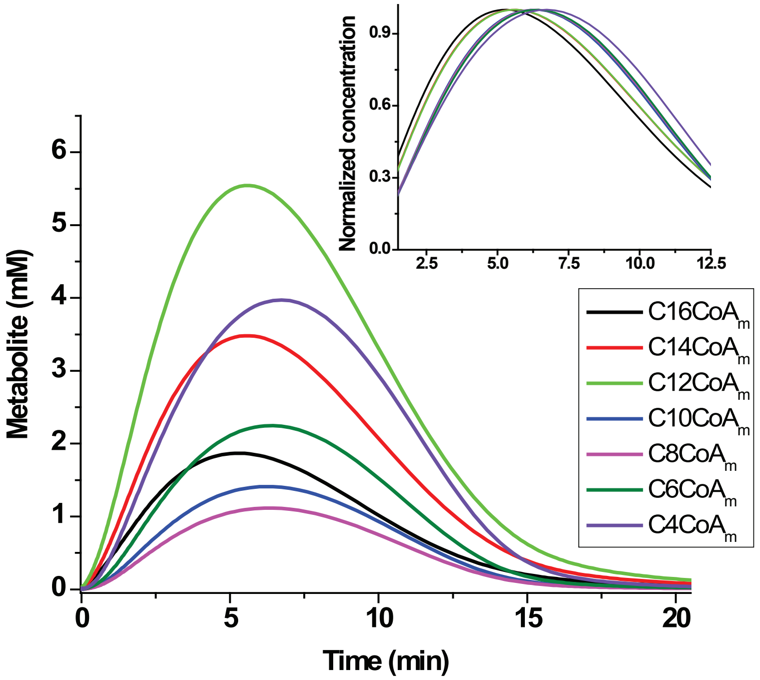

Figure A. Time course of mitochondrial Acyl-CoA intermediates of β-oxidation following an extra-mitochondrial pulse of PCoA. The pulse was simulated with an exponential decay function from a spike of 20μM $PCoA_{cy}$. The inset shows the relative levels of the same intermediates (same color key as in the main panel) with respect to their maximal absolute concentration (shown in the main panel) to enable comparison of their kinetics of appearance and disappearance.

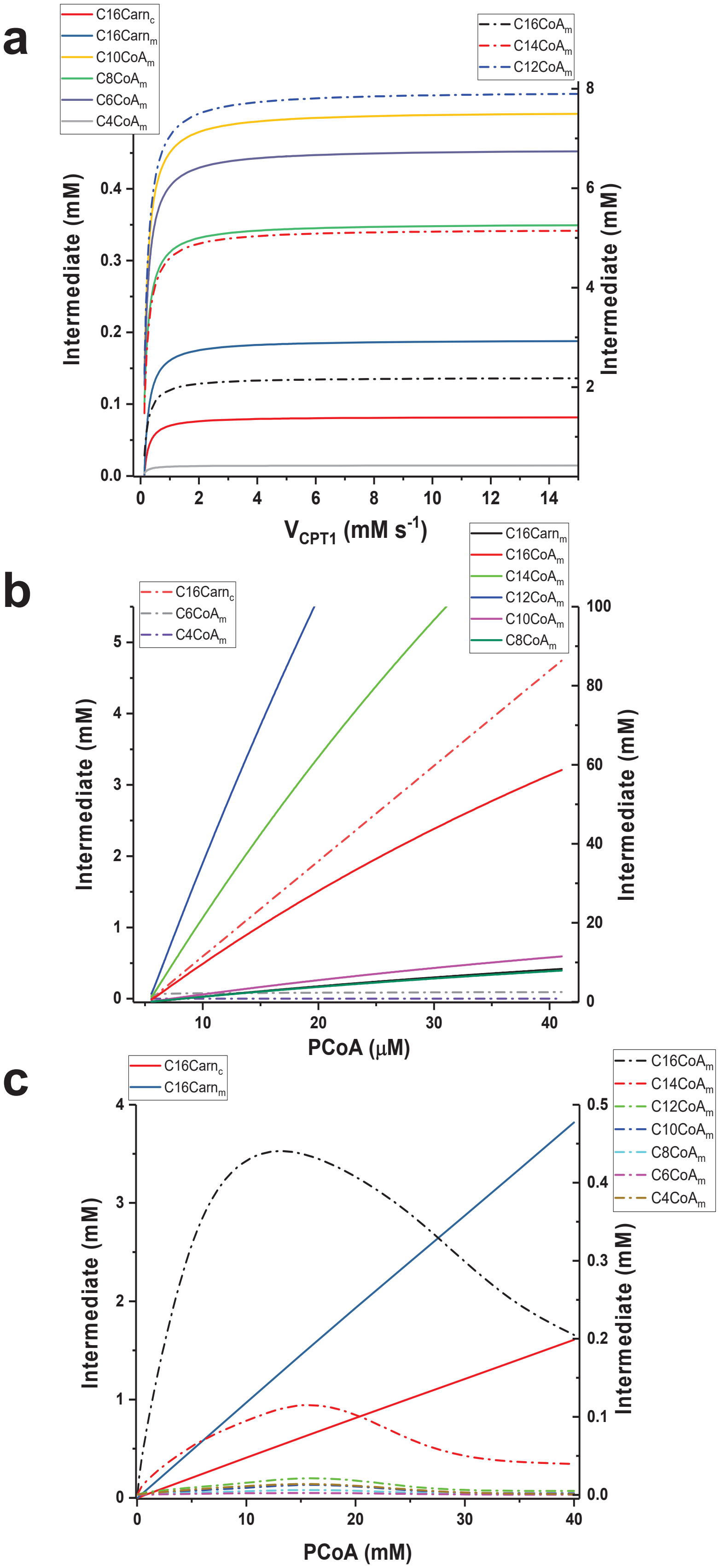

Figure B. Steady state behavior of Acyl-CoA intermediates in the β-oxidation model, before (a, b) and after (c) integration to the bi-compartmental mitochondrial energetic-redox model [2]. The steady state was analyzed as a function of the Vmax of CPT1 (V_{CPT1}) and the extra-mitochondrial level of PCoA. In panel a, the Vmax of CPT1 was varied between 0.13 and 15 mM s^{-1} at fixed 20μM PCoA. Panel b corresponds to the same β-oxidation model where PCoA was studied in the range from 5 to 40μM at fixed V_{CPT1} corresponding to 0.2 mM s^{-1}. Panel c depicts the behavior of Acyl-CoA intermediates in the integrated model encompassing β-oxidation, TCA cycle, oxidative phosphorylation, ion transport and ROS metabolism as described in Sections S1-S6 below. For the same parameters as in the fatty acid oxidation in panel b, the accumulation of Acyl-CoA is limited due to the restrictions imposed by the rates of $FADH_2$ and NADH oxidation in the respiratory chain as well as the complete oxidation of AcCoA in the TCA cycle.

1. 1. Transport of activated fatty acid into the mitochondrial matrix: Carnitine palmitoyl transferase I (Eq. S1), Carnitine Acyl carnitine translocase (Eq. S2) and Carnitine Palmitoyl transferase II (Eq. S3). The enzymes' rate expressions were modelled as bi-reactant reversible reactions, with CPT1 being inhibited by cytoplasmic Malonyl Coenzyme A [1]. As a result of the three successive steps catalyzed by the above-mentioned transporters/enzymes, PCoA is transported from the cytoplasmic into the mitochondrial matrix compartment where β-oxidation takes place.

$$V_{cpt1}^{C16} = sf_{cpt1}^{C16}.V_{cpt1}\frac{\left(\frac{C16CoA_c}{K_{M\,cpt1}^{C16CoAc}}.\frac{Carn_c}{K_{M\,cpt1}^{Carn\,c}}\right)-\left(\frac{C16Carn_c}{K_{M\,cpt1}^{C16CoAc}}.\frac{CoA_c}{K_{M\,cpt1}^{Carn\,c}K_{EQ}^{cpt1}}\right)}{\left(1+\frac{C16CoA_c}{K_{M\,cpt1}^{C16CoAc}}+\frac{C16Carn_c}{K_{M\,cpt1}^{C16Carn\,c}}+\left(\frac{MalCoA_c}{K_{i\,cpt1}^{MalCoAc}}\right)^{n\,cpt1}\right)\left(1+\frac{Carn_c}{K_{M\,cpt1}^{Carn\,c}}+\frac{CoA_c}{K_{M\,cpt1}^{CoAc}}\right)} \quad \text{(S1)}$$

$$V_{cact}^{C16} = V_{f\,cact}\frac{\left(C16Carn_c.Carn_m\right)-\left(C16Carn_m.\frac{Carn_c}{K_{EQ}^{cact1}}\right)}{\left(\begin{array}{c}C16Carn_c.Carn_m+K_{M\,cact}^{Carn\,m}C16Carn_c+K_{M\,cact}^{C16Carn\,c}.Carn_m\left(1+\frac{Carn_c}{K_{i\,cact}^{Carn\,c}}\right)+\cdots\\ \frac{V_{f\,cact}}{V_{r\,cact}K_{EQ}^{cact}}\left(K_{M\,cact}^{Carn\,c}C16Carn_m\left(1+\frac{C16Carn_c}{K_{i\,cact}^{C16Carn\,c}}\right)+Carn_c.\left(K_{M\,cact}^{C16Carn\,m}+C16Carn_m\right)\right)\end{array}\right)} \quad \text{(S2)}$$

$$V_{cpt2}^{C16} = sf_{cpt2}^{C16}.V_{cpt2}\frac{\left(\frac{C16Carn_m}{K_{M\,cpt2}^{C16Carn\,m}}.\frac{CoA_m}{K_{M\,cpt2}^{CoA\,m}}\right)-\left(\frac{C16CoA_m}{K_{M\,cpt2}^{C16Carn\,m}}.\frac{Carn_m}{K_{M\,cpt2}^{CoA\,m}K_{EQ}^{cpt2}}\right)}{\left(1+\frac{C16Carn_m}{K_{M\,cpt2}^{C16Carn\,m}}+\frac{C16CoA_m}{K_{M\,cpt2}^{C16CoA\,m}}\right)\left(1+\frac{Carn_m}{K_{M\,cpt2}^{Carn\,m}}+\frac{CoA_m}{K_{M\,cpt2}^{CoA\,m}}\right)} \quad \text{(S3)}$$

Table A. Parameter values for fatty acids transport into the mitochondrial matrix

Symbol	Value[a]	Units	Description	Eq.
sf_{cpt1}^{C16}	1.0		Scale factor of CPT1 for PalmitoylCarnitine (Pcarnit)	S1
V_{cpt1}	1.0×10^{-3}	mM ms^{-1}	Maximal rate of CPT1	S1
$K_{M\,cpt1}^{C16CoAc}$	13.8	µM	CPT1 Michaelis constant (K_M) for PCoA	S1
$K_{M\,cpt1}^{Carn\,c}$	125	µM	CPT1 K_M for Carnitine	S1
$K_{i\,cpt1}^{MalCoAc}$	9.1	µM	CPT1 inhibition constant for Malonyl CoA	S1
$K_{M\,cpt1}^{C16Carn\,c}$	136	µM	CPT1 K_M for Pcarnit	S1

Symbol	Value	Units	Description	Eq.
$K_{M\,cpt1}^{CoA\,c}$	40.7	μM	CPT1 K_M for Coenzyme A (CoA)	S1
$C16CoA_c$	0-40	μM	Concentration of cytoplasmic PCoA	S1
$MalCoA_c$	0.0	μM	Extra-matrix concentration of Malonyl CoA	S1
$n\ cpt1$	2.48		CPT1 Hill Coefficient for MalCoAc	S1
K_{EQ}^{cpt1}	0.45		CPT1 equilibrium constant	S1
$V_{f\,cact}$	0.7	mM ms^{-1}	Forward maximal rate of carnitine acyl carnitine translocase (CACT)	S2
$V_{r\,cact}$	0.7	mM ms^{-1}	CACT reverse rate	S2
$K_{M\,cact}^{Carn\,m}$	0.13	mM	CACT K_M for Carnitine (mito)	S2
$K_{M\,cact}^{Carn\,c}$	1.3	mM	CACT K_M for Carnitine (cyto)	S2
$K_{i\,cact}^{C16Carn\,c}$	15	μM	CACT inhibition constant for Pcarnit	S2
$K_{i\,cact}^{Carn\,c}$	0.2	mM	CACT inhibition constant for Carnitine	S2
$K_{M\,cact}^{C16Carn\,m}$	0.15	mM	CACT K_M for Pcarnit (mito)	S2
$K_{M\,cact}^{C16Carn\,c}$	0.56	mM	CACT K_M for Pcarnit (cyto)	S2
$Carn_c$	0.4	mM	Cytoplasmic Carnitine	S1, S2
$Carn_m$	0.95	mM	Mitochondrial Carnitine	S2, S3
K_{EQ}^{cact1}	1	-	CACT equilibrium constant	S2
sf_{cpt2}^{C16}	0.85		Scale factor of carnitine palmitoyl transferase2 (CPT2) for Pcarnit	S3
V_{cpt2}	1.955 10^{-2}	mM ms^{-1}	CPT2 forward maximal rate	S3
$K_{M\,cpt2}^{C16Carn\,m}$	51	μM	CPT2 K_M for Pcarnit (mito)	S3
$K_{M\,cpt2}^{C16CoA\,m}$	38	μM	CPT2 K_M for PCoA (mito)	S3
$K_{M\,cpt2}^{CoA\,m}$	30		CPT2 K_M for CoA (mito)	S3

Symbol	Value	Units	Description	Eq.
$K_{M\,cpt2}^{Carn\,m}$	0.35	mM	CPT2 K_M for Carnitine (mito)	S3
K_{EQ}^{cpt2}	2.22		CPT2 equilibrium constant	S3

[a] Parameter values were taken from [1]with the exception of V_{cpt1}, $V_{f\,cact}$, $V_{r\,cact}$ and V_{cpt2} that were adjusted on the basis of fluxes of palmitate oxidation reported in adult cardiomyocytes [3] which renders β-oxidation Vmax values five-fold larger than in liver.

1. 2. Mitochondrial matrix fatty acyl-CoA dehydrogenases

These dehydrogenases catalyze the oxidation of AcylCoA using FAD as electron acceptor. We lumped the dehydrogenase step with the reduction of the FAD group in the electron transfer protein. The FAD group will be re-oxidized by complex II in the respiratory chain as indicated in equation S122 (see below).

$$V_{vlcad}^{DENOMINATOR} = \left(\begin{array}{c} 1 + \frac{C16CoA_m}{K_{M\,vlcad}^{C16CoA\,m}} + \frac{C16enoCoA_m}{K_{M\,vlcad}^{C16enoCoA\,m}} + \frac{C14CoA_m}{K_{M\,vlcad}^{C14CoA\,m}} + \cdots \\ \frac{C14enoCoA_m}{K_{M\,vlcad}^{C14enoCoA\,m}} + \frac{C12CoA_m}{K_{M\,vlcad}^{C12CoA\,m}} + \frac{C12enoCoA_m}{K_{M\,vlcad}^{C12enoCoA\,m}} \end{array} \right) . \left(1 + \frac{ETF_m}{K_{M\,vlcad}^{ETFm}} + \frac{ETFH_{2\,m}}{K_{M\,vlcad}^{ETFH_2\,m} \, K_{EQ}^{vlcad}} \right) \quad \text{(S4)}$$

$$V_{vlcad}^{C16} = sf_{vlcad}^{C16} . V_{vlcad} \frac{\left(\frac{C16CoA_m}{K_{M\,vlcad}^{C16CoA\,m}} . \frac{ETF_m}{K_{M\,vlcad}^{ETFm}} \right) - \left(\frac{C16enoCoA_m}{K_{M\,vlcad}^{C16CoA\,m}} . \frac{ETFH_{2\,m}}{K_{M\,vlcad}^{ETF\,m} \, K_{EQ}^{vlcad}} \right)}{V_{vlcad}^{DENOMINATOR}} \quad \text{(S5)}$$

$$V_{vlcad}^{C14} = sf_{vlcad}^{C14} . V_{vlcad} \frac{\left(\frac{C14CoA_m}{K_{M\,vlcad}^{C14CoA\,m}} . \frac{ETF_m}{K_{M\,vlcad}^{ETFm}} \right) - \left(\frac{C14enoCoA_m}{K_{M\,vlcad}^{C14CoA\,m}} . \frac{ETFH_{2\,m}}{K_{M\,vlcad}^{ETFm} \, K_{EQ}^{vlcad}} \right)}{V_{vlcad}^{DENOMINATOR}} \quad \text{(S6)}$$

$$V_{vlcad}^{C12} = sf_{vlcad}^{C12} . V_{vlcad} \frac{\left(\frac{C12CoA_m}{K_{M\,vlcad}^{C12CoA\,m}} . \frac{ETF_m}{K_{M\,vlcad}^{ETFm}} \right) - \left(\frac{C12enoCoA_m}{K_{M\,vlcad}^{C12CoA\,m}} . \frac{ETFH_{2\,m}}{K_{M\,vlcad}^{ETFm} \, K_{EQ}^{vlcad}} \right)}{V_{vlcad}^{DENOMINATOR}} \quad \text{(S7)}$$

$$V_{lcad}^{DENOMINATOR} = \left(\begin{array}{c} 1 + \frac{C16CoA_m}{K_{M\,lcad}^{C16CoA\,m}} + \frac{C16enoCoA_m}{K_{M\,lcad}^{C16enoCoA\,m}} + \frac{C14CoA_m}{K_{M\,lcad}^{C14CoA\,m}} + \frac{C14enoCoA_m}{K_{M\,lcad}^{C14enoCoA\,m}} + \cdots \\ \frac{C12CoA_m}{K_{M\,lcad}^{C12CoA\,m}} + \frac{C12enoCoA_m}{K_{M\,lcad}^{C12enoCoA\,m}} + \frac{C10CoA_m}{K_{M\,lcad}^{C10CoA\,m}} + \cdots \\ \frac{C10enoCoA_m}{K_{M\,lcad}^{C10enoCoA\,m}} + \frac{C8CoA_m}{K_{M\,lcad}^{C8CoA\,m}} + \frac{C8enoCoA_m}{K_{M\,lcad}^{C8enoCoA\,m}} \end{array} \right) . \left(1 + \frac{ETF_m}{K_{M\,lcad}^{ETFm}} + \frac{ETFH_{2\,m}}{K_{M\,lcad}^{ETFH_2\,m} \, K_{EQ}^{lcad}} \right) \quad \text{(S8)}$$

$$V_{lcad}^{C16} = sf_{lcad}^{C16} . V_{lcad} \frac{\left(\frac{C16CoA_m}{K_{M\,lcad}^{C16CoA\,m}} . \frac{ETF_m}{K_{M\,lcad}^{ETFm}} \right) - \left(\frac{C16enoCoA_m}{K_{M\,lcad}^{C16CoA\,m}} . \frac{ETFH_{2\,m}}{K_{M\,lcad}^{ETFm}\,K_{EQ}^{lcad}} \right)}{V_{lcad}^{DENOMINATOR}} \quad \text{(S9)}$$

$$V_{lcad}^{C14} = sf_{lcad}^{C14} . V_{lcad} \frac{\left(\frac{C14CoA_m}{K_{M\,lcad}^{C14CoA\,m}} . \frac{ETF_m}{K_{M\,lcad}^{ETFm}} \right) - \left(\frac{C14enoCoA_m}{K_{M\,lcad}^{C14CoA\,m}} . \frac{ETFH_{2\,m}}{K_{M\,lcad}^{ETFm}\,K_{EQ}^{lcad}} \right)}{V_{lcad}^{DENOMINATOR}} \quad \text{(S10)}$$

$$V_{lcad}^{C12} = sf_{lcad}^{C12} . V_{lcad} \frac{\left(\frac{C12CoA_m}{K_{M\,lcad}^{C12CoA\,m}} . \frac{ETF_m}{K_{M\,lcad}^{ETFm}} \right) - \left(\frac{C12enoCoA_m}{K_{M\,lcad}^{C12CoA\,m}} . \frac{ETFH_{2\,m}}{K_{M\,lcad}^{ETFm}\,K_{EQ}^{lcad}} \right)}{V_{lcad}^{DENOMINATOR}} \quad \text{(S11)}$$

$$V_{lcad}^{C10} = sf_{lcad}^{C10} . V_{lcad} \frac{\left(\frac{C10CoA_m}{K_{M\,lcad}^{C10CoA\,m}} . \frac{FAD}{K_{M\,lcad}^{ETFm}} \right) - \left(\frac{C10enoCoA_m}{K_{M\,lcad}^{C10CoA\,m}} . \frac{FADH_2}{K_{M\,lcad}^{ETFm}\,K_{EQ}^{lcad}} \right)}{V_{lcad}^{DENOMINATOR}} \quad \text{(S12)}$$

$$V_{lcad}^{C8} = sf_{lcad}^{C8} . V_{lcad} \frac{\left(\frac{C8CoA_m}{K_{M\,lcad}^{C8CoA\,m}} . \frac{FAD_m}{K_{M\,lcad}^{ETFm}} \right) - \left(\frac{C8enoCoA_m}{K_{M\,lcad}^{C8CoA\,m}} . \frac{FADH_{2\,m}}{K_{M\,lcad}^{ETFm}\,K_{EQ}^{lcad}} \right)}{V_{lcad}^{DENOMINATOR}} \quad \text{(S13)}$$

$$V_{mcad}^{DENOMINATOR} = \left(\begin{array}{c} 1 + \frac{C12CoA_m}{K_{M\,mcad}^{C12CoA\,m}} + \frac{C12enoCoA_m}{K_{M\,mcad}^{C12enoCoA\,m}} + \frac{C10CoA_m}{K_{M\,mcad}^{C10CoA\,m}} + \frac{C10enoCoA_m}{K_{M\,mcad}^{C10enoCoA\,m}} + \frac{C8CoA_m}{K_{M\,mcad}^{C8CoA\,m}} + \cdots \\ \frac{C8enoCoA_m}{K_{M\,mcad}^{C8enoCoA\,m}} + \frac{C6CoA_m}{K_{M\,lcad}^{C6CoA\,m}} + \frac{C6enoCoA_m}{K_{M\,mcad}^{C6enoCoA\,m}} + \frac{C4CoA_m}{K_{M\,mcad}^{C4CoA\,m}} + \frac{C4enoCoA_m}{K_{M\,mcad}^{C4enoCoA\,m}} \end{array} \right) . \left(1 + \frac{FAD_m}{K_{M\,mcad}^{ETFm}} + \frac{FADH_{2\,m}}{K_{M\,mcad}^{ETFH_2\,m}\,K_{EQ}^{mcad}} \right) \quad \text{(S14)}$$

$$V_{mcad}^{C12} = sf_{mcad}^{C12} . V_{mcad} \frac{\left(\frac{C12CoA_m}{K_{M\,mcad}^{C12CoA\,m}} . \frac{FAD}{K_{M\,mcad}^{ETFm}} \right) - \left(\frac{C12enoCoA_m}{K_{M\,mcad}^{C12CoA\,m}} . \frac{FADH_2}{K_{M\,mcad}^{ETFm}\,K_{EQ}^{mcad}} \right)}{V_{mcad}^{DENOMINATOR}} \quad \text{(S15)}$$

$$V_{mcad}^{C10} = sf_{mcad}^{C10} . V_{mcad} \frac{\left(\frac{C10CoA_m}{K_{M\,mcad}^{C10CoA\,m}} . \frac{ETF_m}{K_{M\,mcad}^{ETFm}} \right) - \left(\frac{C10enoCoA_m}{K_{M\,mcad}^{C10CoA\,m}} . \frac{ETFH_{2\,m}}{K_{M\,mcad}^{ETFm}\,K_{EQ}^{mcad}} \right)}{V_{mcad}^{DENOMINATOR}} \quad \text{(S16)}$$

$$V_{mcad}^{C8} = sf_{mcad}^{C8} . V_{mcad} \frac{\left(\frac{C8CoA_m}{K_{M\,mcad}^{C8CoA\,m}} . \frac{ETF_m}{K_{M\,mcad}^{ETFm}} \right) - \left(\frac{C8enoCoA_m}{K_{M\,mcad}^{C8CoA\,m}} . \frac{ETFH_{2\,m}}{K_{M\,mcad}^{ETFm}\,K_{EQ}^{mcad}} \right)}{V_{mcad}^{DENOMINATOR}} \quad \text{(S17)}$$

$$V_{mcad}^{C6} = sf_{mcad}^{C6} \cdot V_{mcad} \frac{\left(\frac{C6CoA_m}{K_{M\,mcad}^{C6CoAm}} \cdot \frac{ETF_m}{K_{M\,mcad}^{ETFm}}\right) - \left(\frac{C6enoCoA_m}{K_{M\,mcad}^{C6CoAm}} \cdot \frac{ETFH_{2\,m}}{K_{M\,mcad}^{ETFm}\,K_{EQ}^{mcad}}\right)}{V_{mcad}^{DENOMINATOR}} \quad \text{(S18)}$$

$$V_{mcad}^{C4} = sf_{mcad}^{C4} \cdot V_{mcad} \frac{\left(\frac{C4CoA_m}{K_{M\,mcad}^{C4CoAm}} \cdot \frac{ETF_m}{K_{M\,mcad}^{ETFm}}\right) - \left(\frac{C4enoCoA_m}{K_{M\,mcad}^{C4CoAm}} \cdot \frac{ETFH_{2\,m}}{K_{M\,mcad}^{ETFm}\,K_{EQ}^{mcad}}\right)}{V_{mcad}^{DENOMINATOR}} \quad \text{(S19)}$$

$$V_{scad}^{DENOMINATOR} = \left(1 + \frac{C6CoA_m}{K_{M\,scad}^{C6CoAm}} + \frac{C6enoCoA_m}{K_{M\,scad}^{C6enoCoAm}} + \frac{C4CoA_m}{K_{M\,scad}^{C4CoAm}} + \frac{C4enoCoA_m}{K_{M\,scad}^{C4enoCoAm}}\right) \cdot \left(1 + \frac{ETF_m}{K_{M\,scad}^{ETFm}} + \frac{ETFH_{2\,m}}{K_{M\,scad}^{ETFH_2m}\,K_{EQ}^{scad}}\right) \quad \text{(S20)}$$

$$V_{scad}^{C6} = sf_{scad}^{C6} \cdot V_{scad} \frac{\left(\frac{C6CoA_m}{K_{M\,scad}^{C6CoAm}} \cdot \frac{ETF_m}{K_{M\,scad}^{ETFm}}\right) - \left(\frac{C6enoCoA_m}{K_{M\,scad}^{C6CoAm}} \cdot \frac{ETFH_{2\,m}}{K_{M\,scad}^{ETFm}\,K_{EQ}^{scad}}\right)}{V_{scad}^{DENOMINATOR}} \quad \text{(S21)}$$

$$V_{scad}^{C4} = sf_{scad}^{C4} \cdot V_{scad} \frac{\left(\frac{C4CoA_m}{K_{M\,scad}^{C4CoAm}} \cdot \frac{ETF_m}{K_{M\,scad}^{ETFm}}\right) - \left(\frac{C4enoCoA_m}{K_{M\,scad}^{C4CoAm}} \cdot \frac{ETFH_{2\,m}}{K_{M\,scad}^{ETFm}\,K_{EQ}^{scad}}\right)}{V_{scad}^{DENOMINATOR}} \quad \text{(S22)}$$

Table B. Parameter values for fatty acyl-CoA dehydrogenases (cad)

Symbol	Value[b]	Units	Description	Eq.
sf_{vlcad}^{C16}	1		Scaling factor of very long acyl CoA dehydrogenase (vlcad) for PCoA	S5
sf_{vlcad}^{C14}	0.8		vlcad scaling factor for C14CoA	S6
sf_{vlcad}^{C12}	0.42		vlcad scaling factor for C12CoA	S7
V_{vlcad}	2.0×10^{-3}	mM ms^{-1}	vlcad maximal rate	S5, S6, S7
$K_{M\,vlcad}^{ETFm}$	0.12	μM	vlcad Michaelis constant (K_M) for FAD	S4-S7
$K_{M\,vlcad}^{ETFH_2m}$	24.2	μM	vlcad K_M for $FADH_2$	S4-S7
$K_{M\,vlcad}^{C16CoAm}$	6.5	μM	vlcad K_M for PCoA	S4, S5
$K_{M\,vlcad}^{C16enoCoAm}$	1.08	μM	vlcad K_M for C16enoyl-CoA	S4
$K_{M\,vlcad}^{C14CoAm}$	4.0	μM	vlcad K_M for C14-CoA	S4, S6

Symbol	Value[b]	Units	Description	Eq.
$K_{M\,vlcad}^{C14enoCoAm}$	1.08	μM	vlcad K_M for C14enoyl-CoA	S4
$K_{M\,vlcad}^{C12CoAm}$	2.7	μM	vlcad K_M for C12-CoA	S4, S7
$K_{M\,vlcad}^{C12enoCoAm}$	1.08	μM	vlcad K_M for C12enoyl-CoA	S4
K_{EQ}^{vlcad}	6.0		vlcad equilibrium constant	S4-S7
sf_{lcad}^{C16}	0.9		Scaling factor of long-chain acyl CoA dehydrogenase (lcad) for PCoA	S9
sf_{lcad}^{C14}	1		lcad scaling factor for C14CoA	S10
sf_{lcad}^{C12}	0.95		lcad scaling factor for C12CoA	S11
sf_{lcad}^{C10}	0.85		lcad scaling factor for C10CoA	S12
sf_{lcad}^{C8}	0.4		lcad scaling factor for C8CoA	S13
V_{lcad}	$2.5.10^{-3}$	mM ms^{-1}	lcad maximal rate	S9-S13
$K_{M\,lcad}^{ETFm}$	1.2	nM	lcad K_M for FAD	S8-S13
$K_{M\,lcad}^{ETFH_2m}$	24.2	μM	lcad K_M for $FADH_2$	S8-S13
$K_{M\,lcad}^{C16CoAm}$	2.5	μM	lcad K_M for PCoA	S8,S9
$K_{M\,lcad}^{C16enoCoAm}$	1.08	μM	lcad K_M for C16Enoyl-CoA	S8
$K_{M\,lcad}^{C14CoAm}$	7.4	μM	lcad K_M for C14-CoA	S8,S10
$K_{M\,lcad}^{C14enoCoAm}$	1.08	μM	lcad K_M for C14enoyl-CoA	S8
$K_{M\,lcad}^{C12CoAm}$	9.0	μM	lcad K_M for C12-CoA	S8,S11
$K_{M\,lcad}^{C12enoCoAm}$	1.08	μM	lcad K_M for C12enoyl-CoA	S8
$K_{M\,vlcad}^{C10CoAm}$	24.3	μM	lcad K_M for C10-CoA	S8,S12
$K_{M\,vlcad}^{C10enoCoAm}$	1.08	μM	lcad K_M for C10enoyl-CoA	S8
$K_{M\,vlcad}^{C8CoAm}$	12.3	μM	lcad K_M for C8-CoA	S8,S13
$K_{M\,vlcad}^{C8enoCoAm}$	1.08	μM	lcad K_M for C8-enoyl-CoA	S8

Symbol	Value[b]	Units	Description	Eq.
K_{EQ}^{lcad}	6.0		lcad equilibrium constant	S8-S13
sf_{mcad}^{C12}	0.68		Scaling factor of medium-chain acyl CoA dehydrogenase (mcad) for C12CoA	S15
sf_{mcad}^{C10}	0.8		mcad scaling factor for C10CoA	S16
sf_{mcad}^{C8}	0.87		mcad scaling factor for C8CoA	S17
sf_{mcad}^{C6}	1.0		mcad scaling factor for C6CoA	S18
sf_{mcad}^{C4}	0.12		mcad scaling factor for C4CoA	S19
V_{mcad}	0.25	mM ms^{-1}	mcad maximal rate	S15-S19
$K_{M\,mcad}^{ETFm}$	1.2	nM	mcad K_M for FAD	S14-S19
$K_{M\,mcad}^{ETFH_2m}$	24.2	μM	mcad K_M for $FADH_2$	S14
$K_{M\,mcad}^{C12CoAm}$	5.7	μM	mcad K_M for C12CoA	S14, S15
$K_{M\,mcad}^{C12enoCoAm}$	1.08	μM	mcad K_M for C12Enoyl-CoA	S14
$K_{M\,mcad}^{C10CoAm}$	5.4	μM	mcad K_M for C10-CoA	S14,S16
$K_{M\,mcad}^{C10enoCoAm}$	1.08	μM	mcad K_M for C10enoyl-CoA	S14
$K_{M\,mcad}^{C8CoAm}$	4.0	μM	mcad K_M for C8-CoA	S14,S17
$K_{M\,mcad}^{C8enoCoAm}$	1.08	μM	mcad K_M for C8enoyl-CoA	S14
$K_{M\,mcad}^{C6CoAm}$	9.4	μM	mcad K_M for C6-CoA	S14,S18
$K_{M\,mcad}^{C6enoCoAm}$	1.08	μM	mcad K_M for C6enoyl-CoA	S14
$K_{M\,mcad}^{C4CoAm}$	135	μM	mcad K_M for C4-CoA	S14,S19
$K_{M\,mcad}^{C4enoCoAm}$	1.08	μM	mcad K_M for C4enoyl-CoA	S14
K_{EQ}^{mcad}	6.0		mcad equilibrium constant	S14-S19
sf_{scad}^{C6}	0.3		Scaling factor of short-chain acyl CoA dehydrogenase (scad) for C6CoA	S21
sf_{scad}^{C4}	1.0		scad scaling factor for C4CoA	S22

Symbol	Value[b]	Units	Description	Eq.
V_{scad}	0.25	mM ms^{-1}	scad maximal rate	S21,S22
$K_{M\,scad}^{ETFm}$	0.12	μM	scad K_M for FAD	S20-S22
$K_{M\,scad}^{ETFH_2m}$	24.2	μM	scad K_M for $FADH_2$	S20
$K_{M\,scad}^{C6CoAm}$	285	μM	scad K_M for C6-CoA	S20, S21
$K_{M\,mcad}^{C6enoCoAm}$	1.08	μM	scad K_M for C6Enoyl-CoA	S20
$K_{M\,scad}^{C4CoAm}$	10.7	μM	scad K_M for C4-CoA	S20, S22
$K_{M\,scad}^{C4enoCoAm}$	1.08	μM	scad K_M for C4Enoyl-CoA	S20
K_{EQ}^{scad}	6.0		scad equilibrium constant	S20-S22

[b] V_{vlcad}, V_{lcad}, V_{mcad} and V_{scad} were adjusted following the criteria to reproduce palmitate oxidation fluxes reported in adult cardiomyocytes [3].

1. 3. Fatty acid enoyl-CoA hydratase (Crotonase, CRO)

The enzyme rate expressions were modelled as single substrate reversible reactions, with the various enoyl CoA of different chain length and hydroxyacyl CoA competing with one another [1].

$$V_{cro}^{DENOMINATOR} = \begin{pmatrix} 1+\frac{C16enoCoA_m}{K_{M\,cro}^{C16enoCoAm}}+\frac{C16OHCoA_m}{K_{M\,cro}^{C16OHCoAm}}+\frac{C14enoCoA_m}{K_{M\,cro}^{C14enoCoAm}}+\frac{C14OHCoA_m}{K_{M\,cro}^{C14OHCoAm}}+\cdots \\ \frac{C12enoCoA_m}{K_{M\,cro}^{C12enoCoAm}}+\frac{C12OHCoA_m}{K_{M\,cro}^{C12OHCoAm}}+\frac{C10enoCoA_m}{K_{M\,cro}^{C10enoCoAm}}+\frac{C10OHCoA_m}{K_{M\,cro}^{C10OHCoAm}}+\cdots \\ \frac{C8enoCoA_m}{K_{M\,cro}^{C8enoCoAm}}+\frac{C8OHCoA_m}{K_{M\,cro}^{C8OHCoAm}}+\frac{C6enoCoA_m}{K_{M\,cro}^{C6enoCoAm}}+\frac{C6OHCoA_m}{K_{M\,cro}^{C6OHCoAm}}+\cdots \\ \frac{C4enoCoA_m}{K_{M\,cro}^{C4enoCoAm}}+\frac{C4OHCoA_m}{K_{M\,cro}^{C4OHCoAm}} \end{pmatrix} + \frac{AceAcCoA_m}{K_{i\,cro}^{AcAcCoAm}} \quad \text{(S23)}$$

$$V_{cro}^{C16} = sf_{cro}^{C16}.V_{cro}\frac{\left(\frac{C16enoCoA_m}{K_{M\,cro}^{C16enoCoAm}}\right)-\left(\frac{C16OHCoA_m}{K_{M\,cro}^{C16enoCoAm}K_{EQ}^{cro}}\right)}{V_{cro}^{DENOMINATOR}} \quad \text{(S24)}$$

$$V_{cro}^{C14} = sf_{cro}^{C14}.V_{cro}\frac{\left(\frac{C14enoCoA_m}{K_{M\,cro}^{C14enoCoAm}}\right)-\left(\frac{C14OHCoA_m}{K_{M\,cro}^{C14enoCoAm}K_{EQ}^{cro}}\right)}{V_{cro}^{DENOMINATOR}} \quad \text{(S25)}$$

$$V_{cro}^{C12} = sf_{cro}^{C12} . V_{cro} \frac{\left(\frac{C12enoCoA_m}{K_{M\,cro}^{C12enoCoA\,m}}\right) - \left(\frac{C12OHCoA_m}{K_{M\,cro}^{C12enoCoA\,m} K_{EQ}^{cro}}\right)}{V_{cro}^{DENOMINATOR}} \quad \text{(S26)}$$

$$V_{cro}^{C10} = sf_{cro}^{C10} . V_{cro} \frac{\left(\frac{C10enoCoA_m}{K_{M\,cro}^{C10enoCoA\,m}}\right) - \left(\frac{C10OHCoA_m}{K_{M\,cro}^{C10enoCoA\,m} K_{EQ}^{cro}}\right)}{V_{cro}^{DENOMINATOR}} \quad \text{(S27)}$$

$$V_{cro}^{C8} = sf_{cro}^{C8} . V_{cro} \frac{\left(\frac{C8enoCoA_m}{K_{M\,cro}^{C8enoCoA\,m}}\right) - \left(\frac{C8OHCoA_m}{K_{M\,cro}^{C8enoCoA\,m} K_{EQ}^{cro}}\right)}{V_{cro}^{DENOMINATOR}} \quad \text{(S28)}$$

$$V_{cro}^{C6} = sf_{cro}^{C6} . V_{cro} \frac{\left(\frac{C6enoCoA_m}{K_{M\,cro}^{C6enoCoA\,m}}\right) - \left(\frac{C6OHCoA_m}{K_{M\,cro}^{C6enoCoA\,m} K_{EQ}^{cro}}\right)}{V_{cro}^{DENOMINATOR}} \quad \text{(S29)}$$

$$V_{cro}^{C4} = sf_{cro}^{C4} . V_{cro} \frac{\left(\frac{C4enoCoA_m}{K_{M\,cro}^{C4enoCoA\,m}}\right) - \left(\frac{C4OHCoA_m}{K_{M\,cro}^{C4enoCoA\,m} K_{EQ}^{cro}}\right)}{V_{cro}^{DENOMINATOR}} \quad \text{(S30)}$$

Table C. Parameter values for fatty acylenoyl-CoA hydratase (cro)

Symbol	Value[c]	Units	Description	Eq.
sf_{cro}^{C16}	0.13		cro scaling factor for C16enoylCoA	S24
sf_{cro}^{C14}	0.2		cro scaling factor for C14enoylCoA	S25
sf_{cro}^{C12}	0.25		cro scaling factor for C12enoylCoA	S26
sf_{cro}^{C10}	0.33		cro scaling factor for C10enoylCoA	S27
sf_{cro}^{C8}	0.58		cro scaling factor for C8enoylCoA	S28
sf_{cro}^{C6}	0.8		cro scaling factor for C6enoylCoA	S29
sf_{cro}^{C4}	1.0		cro scaling factor for C4enoylCoA	S30
V_{cro}	0.27	mM ms^{-1}	cro maximal rate	S24-S30

Symbol	Value	Units	Description	Eq.
$K_{M\,cro}^{C16enoCoAm}$	0.15	mM	cro Michaelis constant (K_M) for C16Enoyl-CoA	S23,S24
$K_{M\,cro}^{C16OHCoAm}$	45	μM	cro K_M for C16OH-CoA	S23
$K_{M\,cro}^{C14enoCoAm}$	0.1	mM	cro K_M for C14Enoyl-CoA	S23,S25
$K_{M\,cro}^{C14OHCoAm}$	45	μM	cro K_M for C14OH-CoA	S23
$K_{M\,cro}^{C12enoCoAm}$	25	μM	cro K_M for C12Enoyl-CoA	S23,S26
$K_{M\,cro}^{C12OHCoAm}$	45	μM	cro K_M for C12OH-CoA	S23
$K_{M\,cro}^{C10enoCoAm}$	25	μM	cro K_M for C10Enoyl-CoA	S23,S27
$K_{M\,cro}^{C10OHCoAm}$	45	μM	cro K_M for C10OH-CoA	S23
$K_{M\,cro}^{C8enoCoAm}$	25	μM	cro K_M for C8Enoyl-CoA	S23,S28
$K_{M\,cro}^{C8OHCoAm}$	45	μM	cro K_M for C8OH-CoA	S23
$K_{M\,cro}^{C6enoCoAm}$	25	μM	cro K_M for C6Enoyl-CoA	S23,S29
$K_{M\,cro}^{C6OHCoAm}$	45	μM	cro K_M for C6OH-CoA	S23
$K_{M\,cro}^{C4enoCoAm}$	40	μM	cro K_M for C4Enoyl-CoA	S23,S30
$K_{M\,cro}^{C4OHCoAm}$	45	μM	cro K_M for C4OH-CoA	S23
K_{EQ}^{cro}	3.13		cro equilibrium constant	S23-S30
$K_{i\,cro}^{AcAcCoAm}$	1.6	μM	cro inhibition constant for AcetoAcCoA	S23

$^{c}V_{cro}$ was adjusted following the same criteria indicated in the footnote of Table A. Other parameters were taken from [1].

1. 4. Medium/short-chain hydroxyacyl-CoA dehydrogenase (mschad)

The enzyme's rate expressions were modelled as bi-reactant reversible reactions, coupled to the reduction of NAD^+ and with the various hydroxyacyl CoA and ketoacyl CoA competing for the same enzyme pool [1].

$$V_{MSCHAD}^{DENOMINATOR} = \begin{pmatrix} 1+\frac{C16OHCoA_m}{K_{M\,mschad}^{C16OHCoA\,m}}+\frac{C16ketoCoA_m}{K_{M\,mschad}^{C16ketoCoA\,m}}+\frac{C14OHCoA_m}{K_{M\,mschad}^{C14OHCoA\,m}}+\frac{C14ketoCoA_m}{K_{M\,mschad}^{C14ketoCoA\,m}}+\cdots \\ \frac{C12OHCoA_m}{K_{M\,mschad}^{C12OHCoA\,m}}+\frac{C12ketoCoA_m}{K_{M\,mschad}^{C12ketoCoA\,m}}+\frac{C10OHCoA_m}{K_{M\,mschad}^{C10OHCoA\,m}}+\frac{C10ketoCoA_m}{K_{M\,mschad}^{C10ketoCoA\,m}}+\cdots \\ \frac{C8OHCoA_m}{K_{M\,mschad}^{C8OHCoA\,m}}+\frac{C8ketoCoA_m}{K_{M\,mschad}^{C8ketoCoA\,m}}+\frac{C6OHCoA_m}{K_{M\,mschad}^{C6OHCoA\,m}}+\frac{C6ketoCoA_m}{K_{M\,mschad}^{C6ketoCoA\,m}}+\cdots \\ \frac{C4OHCoA_m}{K_{M\,mschad}^{C4OHCoA\,m}}+\frac{C4ketoCoA_m}{K_{M\,mschad}^{C4ketoCoA\,m}} \end{pmatrix} \left(1+\frac{NAD_m}{K_{M\,mschad}^{NADm}}+\frac{NADH_m}{K_{M\,mschad}^{NADHm}}\right) \quad \text{(S31)}$$

$$V_{mschad}^{C16} = sf_{mschad}^{C16} \cdot V_{mschad} \frac{\left(\frac{C16OHCoA_m}{K_{M\,mschad}^{C16OHCoA\,m}} \cdot \frac{NAD_m}{K_{M\,mschad}^{NADm}}\right) - \left(\frac{C16ketoCoA_m}{K_{M\,mschad}^{C16OHCoA\,m}} \cdot \frac{NADH_m}{K_{M\,mschad}^{NADm}\,K_{EQ}^{mschad}}\right)}{V_{MSCHAD}^{DENOMINATOR}} \quad \text{(S32)}$$

$$V_{mschad}^{C14} = sf_{mschad}^{C14} \cdot V_{mschad} \frac{\left(\frac{C14OHCoA_m}{K_{M\,mschad}^{C14OHCoA\,m}} \cdot \frac{NAD_m}{K_{M\,mschad}^{NADm}}\right) - \left(\frac{C14ketoCoA_m}{K_{M\,mschad}^{C14OHCoA\,m}} \cdot \frac{NADH_m}{K_{M\,mschad}^{NADm}\,K_{EQ}^{mschad}}\right)}{V_{MSCHAD}^{DENOMINATOR}} \quad \text{(S33)}$$

$$V_{mschad}^{C12} = sf_{mschad}^{C12} \cdot V_{mschad} \frac{\left(\frac{C12OHCoA_m}{K_{M\,mschad}^{C12OHCoA\,m}} \cdot \frac{NAD_m}{K_{M\,mschad}^{NADm}}\right) - \left(\frac{C12ketoCoA_m}{K_{M\,mschad}^{C12OHCoA\,m}} \cdot \frac{NADH_m}{K_{M\,mschad}^{NADm}\,K_{EQ}^{mschad}}\right)}{V_{MSCHAD}^{DENOMINATOR}} \quad \text{(S34)}$$

$$V_{mschad}^{C10} = sf_{mschad}^{C10} \cdot V_{mschad} \frac{\left(\frac{C10OHCoA_m}{K_{M\,mschad}^{C10OHCoA\,m}} \cdot \frac{NAD_m}{K_{M\,mschad}^{NADm}}\right) - \left(\frac{C10ketoCoA_m}{K_{M\,mschad}^{C10OHCoA\,m}} \cdot \frac{NADH_m}{K_{M\,mschad}^{NADm}\,K_{EQ}^{mschad}}\right)}{V_{MSCHAD}^{DENOMINATOR}} \quad \text{(S35)}$$

$$V_{mschad}^{C8} = sf_{mschad}^{C8} \cdot V_{mschad} \frac{\left(\frac{C8OHCoA_m}{K_{M\,mschad}^{C8OHCoA\,m}} \cdot \frac{NAD_m}{K_{M\,mschad}^{NADm}}\right) - \left(\frac{C8ketoCoA_m}{K_{M\,mschad}^{C8OHCoA\,m}} \cdot \frac{NADH_m}{K_{M\,mschad}^{NADm}\,K_{EQ}^{mschad}}\right)}{V_{MSCHAD}^{DENOMINATOR}} \quad \text{(S36)}$$

$$V_{mschad}^{C6} = sf_{mschad}^{C6} \cdot V_{mschad} \frac{\left(\frac{C6OHCoA_m}{K_{M\,mschad}^{C6OHCoA\,m}} \cdot \frac{NAD_m}{K_{M\,mschad}^{NADm}}\right) - \left(\frac{C6ketoCoA_m}{K_{M\,mschad}^{C6OHCoA\,m}} \cdot \frac{NADH_m}{K_{M\,mschad}^{NADm}\,K_{EQ}^{mschad}}\right)}{V_{MSCHAD}^{DENOMINATOR}} \quad \text{(S37)}$$

$$V_{mschad}^{C4} = sf_{mschad}^{C4} \cdot V_{mschad} \frac{\left(\frac{C4OHCoA_m}{K_{M\,mschad}^{C4OHCoA\,m}} \cdot \frac{NAD_m}{K_{M\,mschad}^{NADm}}\right) - \left(\frac{C4ketoCoA_m}{K_{M\,mschad}^{C4OHCoA\,m}} \cdot \frac{NADH_m}{K_{M\,mschad}^{NADm}\,K_{EQ}^{mschad}}\right)}{V_{MSCHAD}^{DENOMINATOR}} \quad \text{(S38)}$$

Table D. Parameter values for medium/short-chain hydroxyacyl-CoA dehydrogenase (mschad)

Symbol	Value[d]	Units	Description	Eq.
sf_{mschad}^{C16}	0.6		Scaling factor of mschad for C16OHCoA	S32
sf_{mschad}^{C14}	0.5		Scaling factor of mschad for C14OHCoA	S33
sf_{mschad}^{C12}	0.43		Scaling factor of mschad for C12OHCoA	S34
sf_{mschad}^{C10}	0.64		Scaling factor of mschad for C10OHCoA	S35
sf_{mschad}^{C8}	0.89		Scaling factor of mschad for C8OHCoA	S36
sf_{mschad}^{C6}	1.0		Scaling factor of mschad for C6OHCoA	S37
sf_{mschad}^{C4}	0.67		Scaling factor of mschad for C4OHCoA	S38
V_{mschad}	0.5	mM ms^{-1}	mschad maximal rate	S32-S38
$K_{M\,mschad}^{C16OHCoAm}$	1.5	μM	mschad K_M for C16OHCoA	S31,S32
$K_{M\,mschad}^{C16ketoCoAm}$	1.4	μM	mschad K_M for C16ketoCoA	S31
$K_{M\,mschad}^{C14OHCoAm}$	1.8	μM	mschad K_M for C14OHCoA	S31,S33
$K_{M\,mschad}^{C14ketoCoAm}$	1.4	μM	mschad K_M for C14ketoCoA	S31
$K_{M\,mschad}^{C12OHCoAm}$	3.7	μM	mschad K_M for C12OHCoA	S31,S34
$K_{M\,mschad}^{C12ketoCoAm}$	1.6	μM	mschad K_M for C12ketoCoA	S31
$K_{M\,mschad}^{C10OHCoAm}$	8.8	μM	mschad K_M for C10OHCoA	S31,S35
$K_{M\,mschad}^{C10ketoCoAm}$	2.3	μM	mschad K_M for C10ketoCoA	S31
$K_{M\,mschad}^{C8OHCoAm}$	16.3	μM	mschad K_M for C8OHCoA	S31,S36
$K_{M\,mschad}^{C8ketoCoAm}$	4.1	μM	mschad K_M for C8ketoCoA	S31
$K_{M\,mschad}^{C6OHCoAm}$	28.6	μM	mschad K_M for C6OHCoA	S31,S37
$K_{M\,mschad}^{C6ketoCoAm}$	5.8	μM	mschad K_M for C6ketoCoA	S31
$K_{M\,mschad}^{C4OHCoAm}$	69.9	μM	mschad K_M for C4OHCoA	S31,S38

Symbol	Value	Units	Description	Eq.
$K_{M\ mschad}^{C4ketoCoAm}$	16.9	μM	mschad K_M for C4ketoCoA	S31
$K_{M\ mschad}^{NADm}$	58.5	μM	mschad K_M for NAD^+	S31-S38
$K_{M\ mschad}^{NADHm}$	5.4	μM	mschad K_M for NADH	S31
K_{EQ}^{mschad}	2.17×10^{-4}		mschad equilibrium constant	S32-S38

[d] V_{mschad} was adjusted following the same criteria indicated in Table A. Other parameters were taken from [1].

1. 5. Medium-chain ketoacyl-CoA thiolase (MCKAT)

The cleavage step rate expressions were modelled as bi-reactant reversible reactions, producing Acyl CoA and requiring CoASH as substrate [1].

$$V_{MCKAT}^{DENOMINATOR} = \left(\begin{array}{l} 1+\frac{C16ketoCoA_m}{K_{M\ mckat}^{C16ketoCoAm}}+\frac{C14CoA_m}{K_{M\ mckat}^{C14CoAm}}+\frac{C14ketoCoA_m}{K_{M\ mckat}^{C14ketoCoAm}}+\frac{C12CoA_m}{K_{M\ mckat}^{C12CoAm}}+\cdots \\ \frac{C12ketoCoA_m}{K_{M\ mckat}^{C12ketoCoAm}}+\frac{C10CoA_m}{K_{M\ mckat}^{C10CoAm}}+\frac{C10ketoCoA_m}{K_{M\ mckat}^{C10ketoCoAm}}+\frac{C8CoA_m}{K_{M\ mckat}^{C8CoAm}}+\cdots \\ \frac{C8ketoCoA_m}{K_{M\ mckat}^{C8ketoCoAm}}+\frac{C6CoA_m}{K_{M\ mckat}^{C6CoAm}}+\frac{C6ketoCoA_m}{K_{M\ mckat}^{C6ketoCoAm}}+\frac{C4CoA_m}{K_{M\ mckat}^{C4CoAm}}+\cdots \\ \frac{C4ketoCoA_m}{K_{M\ mckat}^{C4ketoCoAm}}+\frac{AcCoA_m}{K_{M\ mckat}^{AcCoAm}} \end{array} \right) \left(1+\frac{CoA_m}{K_{M\ mckat}^{CoAm}}+\frac{AcCoA_m}{K_{M\ mckat}^{AcCoAm}} \right) \quad (S39)$$

$$V_{mckat}^{C16} = sf_{mckat}^{C16}\cdot V_{mckat}\frac{\left(\frac{C16ketoCoA_m}{K_{M\ mckat}^{C16ketoCoAm}}\cdot\frac{CoA_m}{K_{M\ mckat}^{CoAm}}\right)-\left(\frac{C14CoA_m}{K_{M\ mckat}^{C16ketoCoAm}}\cdot\frac{AcCoA_m}{K_{M\ mckat}^{CoAm}\ K_{EQ}^{mckat}}\right)}{V_{MCKAT}^{DENOMINATOR}} \quad (S40)$$

$$V_{mckat}^{C14} = sf_{mckat}^{C14}\cdot V_{mckat}\frac{\left(\frac{C14ketoCoA_m}{K_{M\ mckat}^{C14ketoCoAm}}\cdot\frac{CoA_m}{K_{M\ mckat}^{CoAm}}\right)-\left(\frac{C12CoA_m}{K_{M\ mckat}^{C14ketoCoAm}}\cdot\frac{AcCoA_m}{K_{M\ mckat}^{CoAm}\ K_{EQ}^{mckat}}\right)}{V_{MCKAT}^{DENOMINATOR}} \quad (S41)$$

$$V_{mckat}^{C12} = sf_{mckat}^{C12}\cdot V_{mckat}\frac{\left(\frac{C12ketoCoA_m}{K_{M\ mckat}^{C12ketoCoAm}}\cdot\frac{CoA_m}{K_{M\ mckat}^{CoAm}}\right)-\left(\frac{C10CoA_m}{K_{M\ mckat}^{C12ketoCoAm}}\cdot\frac{AcCoA_m}{K_{M\ mckat}^{CoAm}\ K_{EQ}^{mckat}}\right)}{V_{MCKAT}^{DENOMINATOR}} \quad (S42)$$

$$V_{mckat}^{C10} = sf_{mckat}^{C10} \cdot V_{mckat} \frac{\left(\frac{C10ketoCoA_m}{K_{M\,mckat}^{C10ketoCoAm}} \cdot \frac{CoA_m}{K_{M\,mckat}^{CoAm}}\right) - \left(\frac{C8CoA_m}{K_{M\,mckat}^{C10ketoCoAm}} \cdot \frac{AcCoA_m}{K_{M\,mckat}^{CoAm}\,K_{EQ}^{mckat}}\right)}{V_{MCKAT}^{DENOMINATOR}} \quad \text{(S43)}$$

$$V_{mckat}^{C8} = sf_{mckat}^{C8} \cdot V_{mckat} \frac{\left(\frac{C8ketoCoA_m}{K_{M\,mckat}^{C8ketoCoAm}} \cdot \frac{CoA_m}{K_{M\,mckat}^{CoAm}}\right) - \left(\frac{C6CoA_m}{K_{M\,mckat}^{C8ketoCoAm}} \cdot \frac{AcCoA_m}{K_{M\,mckat}^{CoAm}\,K_{EQ}^{mckat}}\right)}{V_{MCKAT}^{DENOMINATOR}} \quad \text{(S44)}$$

$$V_{mckat}^{C6} = sf_{mckat}^{C6} \cdot V_{mckat} \frac{\left(\frac{C6ketoCoA_m}{K_{M\,mckat}^{C6ketoCoAm}} \cdot \frac{CoA_m}{K_{M\,mckat}^{CoAm}}\right) - \left(\frac{C4CoA_m}{K_{M\,mckat}^{C6ketoCoAm}} \cdot \frac{AcCoA_m}{K_{M\,mckat}^{CoAm}\,K_{EQ}^{mckat}}\right)}{V_{MCKAT}^{DENOMINATOR}} \quad \text{(S45)}$$

$$V_{mckat}^{C4} = sf_{mckat}^{C4} \cdot V_{mckat} \frac{\left(\frac{C4ketoCoA_m}{K_{M\,mckat}^{C4ketoCoAm}} \cdot \frac{CoA_m}{K_{M\,mckat}^{CoAm}}\right) - \left(\frac{AcCoA_m}{K_{M\,mckat}^{C4ketoCoAm}} \cdot \frac{AcCoA_m}{K_{M\,mckat}^{CoAm}\,K_{EQ}^{mckat}}\right)}{V_{MCKAT}^{DENOMINATOR}} \quad \text{(S46)}$$

Table E. Parameter values for medium-chain ketoacyl-CoA thiolase (mckat)

Symbol	Value[e]	Units	Description	Eq.
sf_{mckat}^{C16}	0.2		mckat scaling factor for C16ketoCoA	S40
sf_{mckat}^{C14}	0.2		mckat scaling factor for C14ketoCoA	S41
sf_{mckat}^{C12}	0.38		mckat scaling factor for C12ketoCoA	S42
sf_{mckat}^{C10}	0.75		mckat scaling factor for C10ketoCoA	S43
sf_{mckat}^{C8}	0.81		mckat scaling factor for C8ketoCoA	S44
sf_{mckat}^{C6}	1.0		mckat scaling factor for C6ketoCoA	S45
sf_{mckat}^{C4}	0.49		mckat scaling factor for C4ketoCoA	S46
V_{mckat}	0.1884	mM ms^{-1}	mckat maximal rate	S40-S46
$K_{M\,mckat}^{CoAm}$	26.6	μM	mckat Michaelis constant (K_M) for CoA	S39-S46
$K_{M\,mckat}^{AcCoAm}$	30		mckat K_M for AcCoA	S39
$K_{M\,mckat}^{C16ketoCoAm}$	1.1	μM	mckat K_M for C16ketoCoA	S39,S40

Symbol	Value	Units	Description	Eq.
$K_{M\,mckat}^{C14CoAm}$	13.83	μM	mckat K_M for C14CoA	S39
$K_{M\,mckat}^{C14ketoCoAm}$	1.2	μM	mckat K_M for C14ketoCoA	S39,S41
$K_{M\,mckat}^{C12CoAm}$	13.83	μM	mckat K_M for C12CoA	S39
$K_{M\,mckat}^{C12ketoCoAm}$	1.3	μM	mckat K_M for C12ketoCoA	S39,S42
$K_{M\,mckat}^{C10CoAm}$	13.83	μM	mckat K_M for C10CoA	S39
$K_{M\,mckat}^{C10ketoCoAm}$	2.1	μM	mckat K_M for C10ketoCoA	S39,S43
$K_{M\,mckat}^{C8CoAm}$	13.83	μM	mckat K_M for C8CoA	S39
$K_{M\,mckat}^{C8ketoCoAm}$	3.2	μM	mckat K_M for C8ketoCoA	S39,S44
$K_{M\,mckat}^{C6CoAm}$	13.83	μM	mckat K_M for C6CoA	S39
$K_{M\,mckat}^{C6ketoCoAm}$	6.7	μM	mckat K_M for C6ketoCoA	S39,S45
$K_{M\,mckat}^{C4CoAm}$	13.83	μM	mckat K_M for C4CoA	S39
$K_{M\,mckat}^{C4ketoCoAm}$	12.4	μM	mckat K_M for C4ketoCoA	S39,S46
K_{EQ}^{mckat}	1051		mckat equilibrium constant	S40-S46

[e] V_{mschad} was adjusted following the same criteria indicated in Table A. Other parameters were taken from [1].

1. 6. Mitochondrial trifunctional protein (mtp)

The multienzyme complex encompassing the steps catalyzed by acylenoyl-CoA hydratase, hydroxyacyl-CoA dehydrogenase and the ketoacyl-CoA thiolase was modeled as a terreactant reversible reaction, using enoyl CoA intermediates, NAD^+ and CoASH and generating AcylCoA, NADH and AcCoA.

$$V_{MTP}^{DENOMINATOR} = \begin{pmatrix} 1+\frac{C16enoCoA_m}{K_{M\,mtp}^{C16enoCoAm}}+\frac{C14CoA_m}{K_{M\,mtp}^{C14CoAm}}+\frac{C14enoCoA_m}{K_{M\,mtp}^{C14enoCoAm}}+\cdots \\ \frac{C12CoA_m}{K_{M\,mtp}^{C12CoAm}}+\frac{C12enoCoA_m}{K_{M\,mtp}^{C12enoCoAm}}+\frac{C10CoA_m}{K_{M\,mtp}^{C10CoAm}}+\cdots \\ \frac{C10enoCoA_m}{K_{M\,mtp}^{C10enoCoAm}}+\frac{C8CoA_m}{K_{M\,mtp}^{C8CoAm}}\frac{C8enoCoA_m}{K_{M\,mtp}^{C8enoCoAm}}+\cdots \\ \frac{C6CoA_m}{K_{M\,mtp}^{C6CoAm}}+\frac{AcetoAcCoA_m}{K_{i\,mtp}^{AcAcCoAm}} \end{pmatrix} \left(1+\frac{CoA_m}{K_{M\,mtp}^{CoAm}}+\frac{AcCoA_m}{K_{M\,mtp}^{AcCoAm}}\right)\left(1+\frac{NAD_m}{K_{M\,mtp}^{NADm}}+\frac{NADH_m}{K_{M\,mtp}^{NADH\,m}}\right) \quad \text{(S47)}$$

$$V_{mtp}^{C16} = sf_{mtp}^{C16} . V_{mtp} \frac{\left(\frac{C16enoCoA_m}{K_{M\,mtp}^{C16enoCoA\,m}} . \frac{CoA_m}{K_{M\,mtp}^{CoAm}} . \frac{NAD_m}{K_{M\,mtp}^{NADm}} \right) - \left(\frac{C14CoA_m}{K_{M\,mtp}^{C16enoCoA\,m}} . \frac{NADH_m}{K_{M\,mtp}^{NADm}} . \frac{AcCoA_m}{K_{M\,mtp}^{CoAm} K_{EQ}^{mtp}} \right)}{V_{MTP}^{DENOMINATOR}} \quad \text{(S48)}$$

$$V_{mtp}^{C14} = sf_{mtp}^{C14} . V_{mtp} \frac{\left(\frac{C14enoCoA_m}{K_{M\,mtp}^{C14enoCoA\,m}} . \frac{CoA_m}{K_{M\,mtp}^{CoAm}} . \frac{NAD_m}{K_{M\,mtp}^{NADm}} \right) - \left(\frac{C12CoA_m}{K_{M\,mtp}^{C14enoCoA\,m}} . \frac{NADH_m}{K_{M\,mtp}^{NADm}} . \frac{AcCoA_m}{K_{M\,mtp}^{CoAm} K_{EQ}^{mtp}} \right)}{V_{MTP}^{DENOMINATOR}} \quad \text{(S49)}$$

$$V_{mtp}^{C12} = sf_{mtp}^{C12} . V_{mtp} \frac{\left(\frac{C12enoCoA_m}{K_{M\,mtp}^{C12enoCoA\,m}} . \frac{CoA_m}{K_{M\,mtp}^{CoAm}} . \frac{NAD_m}{K_{M\,mtp}^{NADm}} \right) - \left(\frac{C10CoA_m}{K_{M\,mtp}^{C12enoCoA\,m}} . \frac{NADH_m}{K_{M\,mtp}^{NADm}} . \frac{AcCoA_m}{K_{M\,mtp}^{CoAm} K_{EQ}^{mtp}} \right)}{V_{MTP}^{DENOMINATOR}} \quad \text{(S50)}$$

$$V_{mtp}^{C10} = sf_{mtp}^{C10} . V_{mtp} \frac{\left(\frac{C10enoCoA_m}{K_{M\,mtp}^{C10enoCoA\,m}} . \frac{CoA_m}{K_{M\,mtp}^{CoAm}} . \frac{NAD_m}{K_{M\,mtp}^{NADm}} \right) - \left(\frac{C8CoA_m}{K_{M\,mtp}^{C10enoCoA\,m}} . \frac{NADH_m}{K_{M\,mtp}^{NADm}} . \frac{AcCoA_m}{K_{M\,mtp}^{CoAm} K_{EQ}^{mtp}} \right)}{V_{MTP}^{DENOMINATOR}} \quad \text{(S51)}$$

$$V_{mtp}^{C8} = sf_{mtp}^{C8} . V_{mtp} \frac{\left(\frac{C8enoCoA_m}{K_{M\,mtp}^{C8enoCoA\,m}} . \frac{CoA_m}{K_{M\,mtp}^{CoAm}} . \frac{NAD_m}{K_{M\,mtp}^{NADm}} \right) - \left(\frac{C6CoA_m}{K_{M\,mtp}^{C8enoCoA\,m}} . \frac{NADH_m}{K_{M\,mtp}^{NADm}} . \frac{AcCoA_m}{K_{M\,mtp}^{CoAm} K_{EQ}^{mtp}} \right)}{V_{MTP}^{DENOMINATOR}} \quad \text{(S52)}$$

Table F. Parameter values for mitochondrial trifunctional protein (mtp)

Symbol	Value[f]	Units	Description	Eq.
sf_{mtp}^{C16}	1.5		mtp scaling factor for C16enoylCoA	S48
sf_{mto}^{C14}	0.9		mtp scaling factor for C14enoylCoA	S49
sf_{mtp}^{C12}	0.81		mtp scaling factor for C12enoylCoA	S50
sf_{mtp}^{C10}	0.73		mtp scaling factor for C10enoylCoA	S51
sf_{mtp}^{C8}	0.64		mtp scaling factor for C8enoylCoA	S52
V_{mtp}	0.142	mM ms^{-1}	mtp maximal rate	S48-S52
$K_{M\,mtp}^{NAD\;m}$	60	μM	mtp Michaelis constant (K_M) for NAD^+	S47-S52
$K_{M\,mtp}^{NADH\,m}$	50	μM	mtp K_M for NADH	S47
$K_{M\,mtp}^{CoA\,m}$	30	μM	mtp K_M for CoA	S47-S52

Symbol	Value	Units	Description	Eq.
$K_{M\,mtp}^{AcCoAm}$	30	μM	mtp K_M for AcCoA	S47
$K_{M\,mtp}^{C16enoCoAm}$	25	μM	mtp K_M for C16 enoylCoA	S47,S48
$K_{M\,mtp}^{C14enoCoAm}$	25	μM	mtp K_M for C14 enoylCoA	S47,S49
$K_{M\,mtp}^{C12enoCoAm}$	25	μM	mtp K_M for C12 enoylCoA	S47,S50
$K_{M\,mtp}^{C10enoCoAm}$	25	μM	mtp K_M for C10 enoylCoA	S47,S51
$K_{M\,mtp}^{C8enoCoAm}$	25	μM	mtp K_M for C8 enoylCoA	S47,S52
$K_{M\,mtp}^{C14CoAm}$	13.83	μM	mtp K_M for C14 CoA	S47
$K_{M\,mtp}^{C12CoAm}$	13.83	μM	mtp K_M for C12 CoA	S47
$K_{M\,mtp}^{C10CoAm}$	13.83	μM	mtp K_M for C10 CoA	S47
$K_{M\,mtp}^{C8CoAm}$	13.83	μM	mtp K_M for C8 CoA	S47
$K_{M\,mtp}^{C6CoAm}$	13.83	μM	mtp K_M for C6 CoA	S47
$K_{M\,mtp}^{AcAcCoAm}$	30	μM	mtp inhibition constant by AcAcCoA	S47
K_{EQ}^{mtp}	0.71		mtp equilibrium constant	S48-S52
$CoASH_T$	1.0	mM	Sum of mitochondrial CoA species	S53
FAD_T	0.7	mM	Total concentration of flavin in ETF	S54

[f] V_{mtp} was adjusted following the same criteria indicated in Table A. Other parameters were taken from [1].

In addition to the conservation relation for NAD^+/NADH, $NADP^+$/NADPH and antioxidant intermediates glutathione and thioredoxin, β-oxidation introduces two conservation relations for species carrying a Coenzyme A group and FAD/$FADH_2$, indicated as follows:

$$\begin{aligned} CoASH = {} & CoASH_T - AcCoA - SCoA - C16CoA - C16enoCoA - C16OHCoA - C16ketoCoA \cdots \\ & - C14CoA - C14enoCoA - C14OHCoA - C14ketoCoA - C12CoA - C12enoCoA \cdots \\ & - C12OHCoA - C12ketoCoA - C10CoA - C10enoCoA - C10OHCoA - C10ketoCoA \cdots \\ & - C8CoA - C8enoCoA - C8OHCoA - C8ketoCoA - C6CoA - C6enoCoA - C6OHCoA \cdots \\ & - C6ketoCoA - C4CoA - C4enoCoA - C4OHCoA - C4ketoCoA \end{aligned} \quad (S53)$$

$$FAD = FAD_T - FADH_2 \tag{S54}$$

2. Appendix. Ordinary differential equations (ODEs)

$$\frac{d[Ca^{2+}]_m}{dt} = \delta_{Ca}\left(V_{uni} - V_{NaCa}\right) \tag{S55}$$

$$\frac{d\ [ADP]_m}{dt} = V_{ANT} - V_{ATPase} - V_{SL} \tag{S56}$$

$$\frac{d\Delta\Psi_m}{dt} = \frac{V_{He} + V_{He(SDH)} + V_{He(ETF)} - V_{Hu} - V_{ANT} - V_{Hleak} - V_{NaCa} - V_{uni} - V_{IMAC}}{C_{mito}} \tag{S57}$$

$$\begin{aligned}\frac{d[NADH]_m}{dt} = &-V_{O_2} + V_{IDH} + V_{KGDH} + V_{MDH} - V_{THD} + V_{mtp}^{C16} + V_{mschad}^{C16} + V_{mtp}^{C14} + V_{mschad}^{C14} + \cdots \\ &V_{mtp}^{C12} + V_{mschad}^{C12} + V_{mtp}^{C10} + V_{mschad}^{C10} + V_{mtp}^{C8} + V_{mschad}^{C8} + V_{mschad}^{C6} + V_{mschad}^{C4}\end{aligned} \tag{S58}$$

$$\frac{d[H^+]_m}{dt} = \delta_H\left(-V_{He} - V_{He(SDH)} - V_{He(ETF)} + V_{Hu} + V_{NaH} + V_{PiC} + V_{Hleak}\right) \tag{S59}$$

$$\frac{d[Pi]_m}{dt} = -V_{ATPase} + V_{PiC} - V_{SL} \tag{S60}$$

$$\frac{d[ISOC]}{dt} = V_{ACO} - V_{IDH} - V_{IDH\_NADP} \tag{S61}$$

$$\frac{d[\alpha KG]}{dt} = V_{IDH} + V_{IDH\_NADP} - V_{KGDH} + V_{ATT} \tag{S62}$$

$$\frac{d[SCoA]}{dt} = V_{KGDH} - V_{SL} \tag{S63}$$

$$\frac{d[Suc]}{dt} = V_{SL} - V_{O_2SDH} \tag{S64}$$

$$\frac{d[FUM]}{dt} = V_{O_2SDH} - V_{FH} \tag{S65}$$

$$\frac{d[MAL]}{dt} = V_{FH} - V_{MDH} \tag{S66}$$

$$\frac{d[OAA]}{dt} = V_{MDH} - V_{CS} - V_{AAT} \tag{S67}$$

$$\frac{d[C16Carn]_i}{dt} = V_{CPT1} - V_{CACT} \tag{S68}$$

$$\frac{d[C16Carn]_m}{dt} = V_{CACT} - V_{CPT2} \quad \text{(S69)}$$

$$\frac{d[C16CoA]_m}{dt} = V_{CPT2} - V_{vlcad}^{C16} - V_{lcad}^{C16} \quad \text{(S70)}$$

$$\frac{d[C16enoylCoA]_m}{dt} = V_{vlcad}^{C16} + V_{lcad}^{C16} - V_{cro}^{C16} - V_{mtp}^{C16} \quad \text{(S71)}$$

$$\frac{d[C16OHCoA]_m}{dt} = V_{cro}^{C16} - V_{mschad}^{C16} \quad \text{(S72)}$$

$$\frac{d[C16ketoCoA]_m}{dt} = V_{mschad}^{C16} - V_{mckat}^{C16} \quad \text{(S73)}$$

$$\frac{d[C14CoA]_m}{dt} = V_{mckat}^{C16} + V_{mtp}^{C16} - V_{vlcad}^{C14} - V_{lcad}^{C14} \quad \text{(S74)}$$

$$\frac{d[C14enoylCoA]_m}{dt} = V_{vlcad}^{C14} + V_{lcad}^{C14} - V_{cro}^{C14} - V_{mtp}^{C14} \quad \text{(S75)}$$

$$\frac{d[C14OHCoA]_m}{dt} = V_{cro}^{C14} - V_{mschad}^{C14} \quad \text{(S76)}$$

$$\frac{d[C14ketoCoA]_m}{dt} = V_{mschad}^{C14} - V_{mckat}^{C14} \quad \text{(S77)}$$

$$\frac{d[C12CoA]_m}{dt} = V_{mckat}^{C14} + V_{mtp}^{C14} - V_{vlcad}^{C12} - V_{lcad}^{C12} - V_{mcad}^{C12} \quad \text{(S78)}$$

$$\frac{d[C12enoylCoA]_m}{dt} = V_{vlcad}^{C12} + V_{lcad}^{C12} + V_{mcad}^{C12} - V_{cro}^{C12} - V_{mtp}^{C12} \quad \text{(S79)}$$

$$\frac{d[C12OHCoA]_m}{dt} = V_{cro}^{C12} - V_{mschad}^{C12} \quad \text{(S80)}$$

$$\frac{d[C12ketoCoA]_m}{dt} = V_{mschad}^{C12} - V_{mckat}^{C12} \quad \text{(S81)}$$

$$\frac{d[C10CoA]_m}{dt} = V_{mckat}^{C12} + V_{mtp}^{C12} - V_{lcad}^{C10} - V_{mcad}^{C10} \quad \text{(S82)}$$

$$\frac{d[C10enoylCoA]_m}{dt} = V_{lcad}^{C10} + V_{mcad}^{C10} - V_{cro}^{C10} - V_{mtp}^{C10} \quad \text{(S83)}$$

$$\frac{d[C10OHCoA]_m}{dt} = V_{cro}^{C10} - V_{mschad}^{C10} \quad \text{(S84)}$$

$$\frac{d[C10ketoCoA]_m}{dt} = V_{mschad}^{C10} - V_{mckat}^{C10} \tag{S85}$$

$$\frac{d[C8CoA]_m}{dt} = V_{mckat}^{C10} + V_{mtp}^{C10} - V_{lcad}^{C8} - V_{mcad}^{C8} \tag{S86}$$

$$\frac{d[C8enoylCoA]_m}{dt} = V_{lcad}^{C8} + V_{mcad}^{C8} - V_{cro}^{C8} - V_{mtp}^{C8} \tag{S87}$$

$$\frac{d[C8OHCoA]_m}{dt} = V_{cro}^{C8} - V_{mschad}^{C8} \tag{S88}$$

$$\frac{d[C8ketoCoA]_m}{dt} = V_{mschad}^{C8} - V_{mckat}^{C8} \tag{S89}$$

$$\frac{d[C6CoA]_m}{dt} = V_{mckat}^{C8} + V_{mtp}^{C8} - V_{scad}^{C6} - V_{mcad}^{C6} \tag{S90}$$

$$\frac{d[C6enoylCoA]_m}{dt} = V_{scad}^{C6} + V_{mcad}^{C6} - V_{cro}^{C6} \tag{S91}$$

$$\frac{d[C6OHCoA]_m}{dt} = V_{cro}^{C6} - V_{mschad}^{C6} \tag{S92}$$

$$\frac{d[C6ketoCoA]_m}{dt} = V_{mschad}^{C6} - V_{mckat}^{C6} \tag{S93}$$

$$\frac{d[C4CoA]_m}{dt} = V_{mckat}^{C6} - V_{scad}^{C4} - V_{mcad}^{C4} \tag{S94}$$

$$\frac{d[C4enoylCoA]_m}{dt} = V_{scad}^{C4} + V_{mcad}^{C4} - V_{cro}^{C4} \tag{S95}$$

$$\frac{d[C4OHCoA]_m}{dt} = V_{cro}^{C4} - V_{mschad}^{C4} \tag{S96}$$

$$\frac{d[C4ketoCoA]_m}{dt} = V_{mschad}^{C4} - V_{mckat}^{C4} \tag{S97}$$

$$\frac{d[AcCoA]_m}{dt} = -V_{CS} + V_{mtp}^{C16} + V_{mckat}^{C16} + V_{mtp}^{C14} + V_{mckat}^{C14} + V_{mtp}^{C12} + V_{mckat}^{C12} + \cdots$$
$$V_{mtp}^{C10} + V_{mckat}^{C10} + V_{mtp}^{C8} + V_{mckat}^{C8} + V_{mckat}^{C6} + 2V_{mckat}^{C4} \tag{S98}$$

$$\frac{d[FADH_2]_m}{dt} = -V_{O_2}^{ETF} + V_{vlcad}^{C16} + V_{lcad}^{C16} + V_{vlcad}^{C14} + V_{lcad}^{C14} + V_{vlcad}^{C12} + V_{lcad}^{C12} + \cdots$$
$$V_{mcad}^{C12} + V_{lcad}^{C10} + V_{mcad}^{C10} + V_{lcad}^{C8} + V_{mcad}^{C8} + V_{mcad}^{C6} + V_{scad}^{C6} + V_{mcad}^{C4} + V_{scad}^{C4} \tag{S99}$$

$$\frac{d[NADPH]_m}{dt} = V_{IDH\_NADP} + V_{THD} - V_{GRm} - V_{TxRm} \tag{S100}$$

$$\frac{d[O_2^{\bullet-}]_m}{dt} = shunt\left(V_{O_2} + V_{O_2SDH}\right) - V_{MnSOD} - V_{ROS}^{Tr} \tag{S101}$$

$$\frac{d[O_2^{\bullet-}]_i}{dt} = \frac{v_m}{v_i} V_{ROS}^{Tr} - V_{CuZnSOD} \tag{S102}$$

$$\frac{d[H_2O_2]_m}{dt} = V_{MnSOD} - V_{difH_2O_2} - V_{GPXm} - V_{TxPXm} \tag{S103}$$

$$\frac{d[H_2O_2]_i}{dt} = V_{CuZnSOD} + \frac{v_m}{v_i} V_{difH_2O_2} - V_{GPXi} - V_{TxPXi} - V_{CAT} \tag{S104}$$

$$\frac{d[GSH]_m}{dt} = V_{GRm} - V_{GPXm} - V_{GRXm} + V_{GST} - V_{PSSGm} \tag{S105}$$

$$\frac{d[GSH]_i}{dt} = V_{GRi} - V_{GPXi} - V_{GRXi} + \frac{v_m}{v_i} V_{GST} - V_{PSSGi} \tag{S106}$$

$$\frac{d[GSSG]_m}{dt} = 0.5\left(V_{GPXm} - V_{GRm}\right) + V_{GRXm} \tag{S107}$$

$$\frac{d[TxR]_m}{dt} = V_{TxRm} - V_{TxPXm} \tag{S108}$$

$$\frac{d[TxR]_i}{dt} = V_{TxRi} - V_{TxPXi} \tag{S109}$$

$$\frac{d[PSSG]_m}{dt} = V_{PSSGm} - V_{GRXm} \tag{S110}$$

$$\frac{d[PSSG]_i}{dt} = V_{PSSGi} - V_{GRXi} \tag{S111}$$

Equations S68-S99 correspond to the intermediates and final products of β-oxidation that will feed the TCA cycle and the respiratory chain.

3. Appendix. Rate equations of mitochondrial energy metabolism

The equations in this and the following sections were included in the two compartment model of mitochondrial energetics, redox and ROS metabolism reported in Kembro et al (2013) [2]. The equations and parameters are reproduced here and details about the model parameterization are in the original publication [2].

3.1. TCA cycle rate equations

$$V_{CS} = \frac{k_{cat}^{CS}\ E_T^{CS}}{\left(1+\frac{K_M^{AcCoA}}{[AcCoA]}\right)\left(1+\frac{K_M^{OAA}}{[OAA]}\right)} \tag{S112}$$

$$V_{ACO} = k_f^{ACO}\left([CIT] - \frac{[ISOC]}{K_E^{ACO}}\right) \tag{S113}$$

$$V_{IDH} = k_{cat}^{IDH} E_T^{IDH} \begin{bmatrix} \left(1+\frac{[H^+]_m}{k_{h,1}}+\frac{k_{h,2}}{[H^+]_m}\right)+f_i^{IDH}\left(\frac{K_{Midh}^{NAD}}{[NAD]}\right)+\ldots \\ f_a^{IDH}\left(\frac{K_M^{ISOC}}{[ISOC]}\right)^{ni}+f_a^{IDH} f_i^{IDH}\left(\frac{K_M^{ISOC}}{[ISOC]}\right)^{ni}\left(\frac{K_{Midh}^{NAD}}{[NAD]}\right) \end{bmatrix}^{-1} \tag{S114}$$

$$f_a^{IDH} = \left[\left(1+\frac{[ADP^{3-}]_m}{K_{ADP}^a}\right)\left(1+\frac{[Ca^{2+}]_m}{K_{Ca}^a}\right)\right]^{-1}$$

$$f_i^{IDH} = \left(1+\frac{[NADH]}{K_{i,NADH}}\right)$$

$$V_{KGDH} = \frac{k_{cat}^{KGDH} E_T^{KGDH}}{1+\frac{[H^+]_m}{k_{h,1a}}+\frac{k_{h,2a}}{[H^+]_m}+f_a^{KGDH}\left(\frac{k_M^{\alpha KG}}{[\alpha KG]}\right)^{n_{\alpha KG}}+f_a^{KGDH}\frac{k_{M\_kgdh}^{NAD}}{[NAD]}} \tag{S115}$$

$$f_a^{KGDH} = \left[\left(1+\frac{[Mg^{2+}]}{K_D^{Mg^{2+}}}\right)\left(1+\frac{[Ca^{2+}]_m}{K_D^{Ca^{2+}}}\right)\right]^{-1}$$

$$V_{SL} = k_f^{SL}\left([SCoA][ADP]_m[Pi]_m - \frac{[Suc][ATP]_m[CoA]}{K_{E,app}^{SL}}\right) \tag{S116}$$

$$K_{E,app}^{SL} = K_{Eq}^{SL}\frac{P_{SUC}P_{ATP}}{P_{Pi}P_{ADP}}$$

Succinate dehydrogenase is included together with the respiratory complexes

$$V_{FH} = k_f^{FH}\left([FUM] - \frac{[MAL]}{K_E^{FH}}\right) \tag{S117}$$

$$V_{MDH} = \frac{k_{cat}^{MDH} E_T^{MDH} f_{h,a} f_{h,i}}{1 + \frac{K_M^{MAL}}{[MAL]}\left(1 + \frac{[OAA]}{K_i^{OAA}}\right) + \frac{K_M^{NAD}}{[NAD]} + \frac{K_M^{MAL}}{[MAL]}\left(1 + \frac{[OAA]}{K_i^{OAA}}\right)\frac{K_M^{NAD}}{[NAD]}} \tag{S118}$$

$$f_{h,a} = \left(1 + \frac{[H^+]}{k_{h1}} + \frac{[H^+]^2}{k_{h1}k_{h2}}\right)^{-1} + k_{offset}$$

$$f_{h,i} = \left(1 + \frac{k_{h3}}{[H^+]} + \frac{k_{h3}k_{h4}}{[H^+]^2}\right)$$

$$V_{AAT} = k_f^{AAT}[OAA][GLU]\frac{k_{ASP}K_E^{ATT}}{\left(k_{ASP}K_E^{AAT} + [\alpha KG]k_f^{AAT}\right)} \tag{S119}$$

Table G. Parameter values of the tricarboxylic acid cycle

Symbol	Value	Units	Description	Eq.
k_{cat}^{CS}	2.35×10^{-4}	ms^{-1}	Catalytic constant of citrate synthase (CS)	S112
E_T^{CS}	0.4	mM	CS concentration	S112
K_M^{AcCoA}	0.0126	mM	CS Michaelis constant for AcCoA	S112
K_M^{OAA}	6.4×10^{-4}	mM	CS Michaelis constant for OAA	S112
$C_{k\text{int}}$	1.3	mM	Sum of TCA cycle intermediates	
C_{PN}	1.0	mM	Sum of pyridine nucleotides	
k_f^{ACO}	1.17×10^{-4}	ms^{-1}	Forward rate constant of aconitase (ACO)	S113
K_E^{ACO}	2.22		ACO equilibrium constant	S113
$K_{i,NADH}$	0.19	mM	Inhibition constant by NADH	S114
k_{cat}^{IDH}	1.188	ms^{-1}	Rate constant of isocitrate dehydrogenase (IDH)	S114

Symbol	Value	Units	Description	Eq.
E_T^{IDH}	0.109	mM	IDH concentration	S114
$k_{h,1}$	1×10^{-5}	mM	IDH ionization constant	S114
$k_{h,2}$	9×10^{-4}	mM	IDH ionization constant	S114
K_M^{ISOC}	1.52	mM	IDH Michaelis constant for isocitrate	S114
n_i	2.0		IDH cooperativity for isocitrate	S114
K_{Midh}^{NAD}	0.923	mM	Michaelis constant for NAD^+	S114
K_{ADP}^{a}	0.62	mM	Activation constant by ADP	S114
K_{Ca}^{a}	5×10^{-4}	mM	IDH activation constant for Ca^{2+}	S114
E_T^{KGDH}	0.5	mM	α-ketoglutarate dehydrogenase (KGDH) concentration	S115
k_{cat}^{KDGH}	0.0132	ms^{-1}	KGDH rate constant	S115
$k_M^{\alpha KG}$	30	mM	KGDH Michaelis constant for alpha-ketoglutarate (αKG)	S115
$K_{M\_kgdh}^{NAD}$	38.7	mM	KGDH Michaelis constant for NAD^+	S115
$k_{h,1a}$	4×10^{-5}	mM	KGDH ionization constant	S115
$k_{h,2a}$	7×10^{-5}	mM	KGDH ionization constant	S115
$K_D^{Mg^{2+}}$	0.0308	mM	Activation constant for Mg^{2+}	S115
$K_D^{Ca^{2+}}$	1.5×10^{-4}	mM	Activation constant for Ca^{2+}	S115
$n_{\alpha KG}$	1.2		KGDH Hill coefficient for α KG	S115
$[Mg^{2+}]_m$	0.4	mM	Mg^{2+} concentration in mitochondria	S115
k_f^{SL}	2.8×10^{-3}	mM^{-1} ms^{-1}	Forward rate constant of succinate lyase (SL)	S116

Symbol	Value	Units	Description	Eq.
K_E^{SL}	3.115		SL equilibrium constant	S116
k_f^{FH}	8.3×10^{-3}	ms^{-1}	Forward rate constant for fumarate hydratase (FH)	S117
K_E^{FH}	1.0		FH equilibrium constant	S118
k_{h1}	1.131×10^{-5}	mM	Ionization constant of malate dehydrogenase (MDH)	S118
k_{h2}	26.7	mM	MDH ionization constant	S118
k_{h3}	6.68×10^{-9}	mM	MDH ionization constant	S118
k_{h4}	5.62×10^{-6}	mM	MDH ionization constant	S118
k_{offset}	3.99×10^{-2}		Offset of MDH pH activation factor	S118
k_{cat}^{MDH}	0.1242	ms^{-1}	MDH rate constant	S118
E_T^{MDH}	0.154	mM	Total MDH concentration	S118
K_M^{MAL}	1.493	mM	MDH Michaelis constant for malate	S118
K_i^{OAA}	0.031	mM	Inhibition constant for oxalacetate	S118
K_M^{NAD}	0.2244	mM	MDH Michaelis constant for NAD^+	S118
[GLU]	1×10^{-3}	mM	Glutamate concentration	S119
k_f^{AAT}	0.0214	ms^{-1}	Forward rate constant of aspartate amino transferase (AAT)	S119
K_E^{AAT}	6.6		AAT equilibrium constant	S119
k_{ASP}	1.5×10^{-6}	ms^{-1}	Rate constant of aspartate consumption	S119

3.2. Oxidative Phosphorylation rate equations

$$V_{O_2} = 0.5\rho^{res} \frac{\left(r_a + r_{c1} e^{\left(\frac{6F\Delta\Psi_B}{RT}\right)}\right) e^{\left(\frac{FA_{res}}{RT}\right)} - r_a e^{\left(\frac{g6F\Delta\mu_H}{RT}\right)} + r_{c2} e^{\left(\frac{FA_{res}}{RT}\right)} e^{\left(\frac{g6F\Delta\mu_H}{RT}\right)}}{\left(1 + r_1 e^{\left(\frac{FA_{res}}{RT}\right)}\right) e^{\left(\frac{6F\Delta\Psi_B}{RT}\right)} + \left(r_2 + r_3 e^{\left(\frac{FA_{res}}{RT}\right)}\right) e^{\left(\frac{g6F\Delta\mu_H}{RT}\right)}} \quad \text{(S120)}$$

$$V_{He} = 6\rho^{res} \frac{\left(r_a e^{\left(\frac{A_{res}F}{RT}\right)} - (r_a + r_b) e^{\left(\frac{g6F\Delta\mu_H}{RT}\right)}\right)}{\left(1 + r_1 e^{\left(\frac{FA_{res}}{RT}\right)}\right) e^{\left(\frac{6F\Delta\Psi_B}{RT}\right)} + \left(r_2 + r_3 e^{\left(\frac{FA_{res}}{RT}\right)}\right) e^{\left(\frac{g6F\Delta\mu_H}{RT}\right)}}$$

$$A_{res} = \frac{RT}{F} \ln\left(K_{res} \sqrt{\frac{[NADH]}{[NAD^+]}}\right)$$

$$V_{O_2SDH} = 0.5\rho^{res(SDH)} \frac{\left(r_a + r_{c1} e^{\left(\frac{4F\Delta\Psi_B}{RT}\right)}\right) e^{\left(\frac{FA_{RSDH}}{RT}\right)} - r_a e^{\left(\frac{g4F\Delta\mu_H}{RT}\right)} + r_{c2} e^{\left(\frac{FA_{RSDH}}{RT}\right)} e^{\left(\frac{g4F\Delta\mu_H}{RT}\right)}}{\left(1 + r_1 e^{\left(\frac{FA_{RSDH}}{RT}\right)}\right) e^{\left(\frac{4F\Delta\Psi_B}{RT}\right)} + \left(r_2 + r_3 e^{\left(\frac{FA_{RSDH}}{RT}\right)}\right) e^{\left(\frac{g4F\Delta\mu_H}{RT}\right)}} \left(\frac{1}{1 + \frac{[OAA]}{K_i^{OAA}}}\right) \quad \text{(S121)}$$

$$V_{He(SDH)} = 4\rho^{res(SDH)} \frac{\left(r_a e^{\left(\frac{A_{RSDH}F}{RT}\right)} - (r_a + r_b) e^{\left(\frac{g4F\Delta\mu_H}{RT}\right)}\right)}{\left(1 + r_1 e^{\left(\frac{FA_{RSDH}}{RT}\right)}\right) e^{\left(\frac{4F\Delta\Psi_B}{RT}\right)} + \left(r_2 + r_3 e^{\left(\frac{FA_{RSDH}}{RT}\right)}\right) e^{\left(\frac{g4F\Delta\mu_H}{RT}\right)}} \left(\frac{1}{1 + \frac{[OAA]}{K_i^{OAA}}}\right)$$

$$A_{RSDH} = \frac{RT}{F} \ln\left(K_{RSDH,app} \sqrt{\frac{[SUC]}{[FUM]}}\right)$$

$$K_{RSDH,app} = \frac{K_{res(SDH)}}{P_{SUC}}$$

$$V_{O_2}^{ETF} = 0.5\rho^{res(ETF)} \frac{\left(r_a + r_{c1}e^{\left(\frac{4F\Delta\Psi_B}{RT}\right)}\right)e^{\left(\frac{FA_{ETF}}{RT}\right)} - r_a e^{\left(\frac{g4F\Delta\mu_H}{RT}\right)} + r_{c2}e^{\left(\frac{FA_{ETF}}{RT}\right)}e^{\left(\frac{g4F\Delta\mu_H}{RT}\right)}}{\left(1 + r_1 e^{\left(\frac{FA_{ETF}}{RT}\right)}\right)e^{\left(\frac{4F\Delta\Psi_B}{RT}\right)} + \left(r_2 + r_3 e^{\left(\frac{FA_{ETF}}{RT}\right)}\right)e^{\left(\frac{g4F\Delta\mu_H}{RT}\right)}} \quad \text{(S122)}$$

$$V_{He(ETF)} = 4\rho^{res(ETF)} \frac{\left(r_a e^{\left(\frac{A_{ETF}F}{RT}\right)} - (r_a + r_b)e^{\left(\frac{g4F\Delta\mu_H}{RT}\right)}\right)}{\left(1 + r_1 e^{\left(\frac{FA_{ETF}}{RT}\right)}\right)e^{\left(\frac{4F\Delta\Psi_B}{RT}\right)} + \left(r_2 + r_3 e^{\left(\frac{FA_{ETF}}{RT}\right)}\right)e^{\left(\frac{g4F\Delta\mu_H}{RT}\right)}} \quad \text{(S122)}$$

$$A_{ETF} = \frac{RT}{F}\ln\left(K_{ETF,app}\sqrt{\frac{[FADH2]}{[FAD]}}\right)$$

$$K_{ETF,app} = \frac{K_{res(FAD)}}{P_{H_2O}}$$

$$V_{ATPase} = -\rho^{F1} \frac{\left(100p_a + p_{c1}\exp(3F\Delta\Psi_B / RT)\right)\exp(A_{F1}F/RT) - \left(\begin{array}{l} p_a \exp(3F\Delta\mu_H / RT) \\ \cdots + p_{c2}\exp(A_{F1}F/RT)\exp(3F\Delta\mu_H / RT)\end{array}\right)}{\left(1 + p_1\exp(A_{F1}F/RT)\right)\exp(3F\Delta\Psi_B / RT) + \left(p_2 + p_3\exp(A_{F1}F/RT)\right)\exp(3F\Delta\mu_H / RT)} \quad \text{(S123)}$$

$$V_{Hu} = -3\rho^{F1} \frac{p_a\left(1 + \exp(A_{F1}F/RT)\right) - \left(p_a + p_b\right)\exp(3F\Delta\mu_H / RT)}{\left(1 + p_1\exp(A_{F1}F/RT)\right)\exp(3F\Delta\Psi_B / RT) + \left(p_2 + p_3\exp(A_{F1}F/RT)\right)\exp(3F\Delta\mu_H / RT)}$$

$$A_{F1} = \frac{RT}{F}\ln(K_{app}^{ATPase}\frac{[MgATP^{2-}]}{[ADP_{free}][Pi_{total}]})$$

$$K_{app}^{ATPase} = K_{eq}^{ATPase}[H^+]^1\frac{P_{ATP}P_{H_2O}}{P_{ADP}P_{Pi}}$$

$$V_{ANT} = \mathrm{V}_{\mathrm{maxANT}} \frac{\left(1 - \frac{[ATP^{4-}]_i \times [ADP^{3-}]_m}{[ADP^{3-}]_i \times [ATP^{4-}]_m}\right)\exp(-F\Delta\Psi_m / RT)}{\left(1 + \frac{[ATP^{4-}]_i}{[ADP^{3-}]_i}\exp(-hF\Delta\Psi / RT)\right)\left(1 + \frac{[ADP^{3-}]_m}{[ATP^{4-}]_m}\right)} \quad \text{(S124)}$$

Table H. Parameter values of oxidative phosphorylation

Symbol	Value	Units	Description	Eq.
r_a	6.394×10^{-13}	ms^{-1}	Sum of products of rate constants	S120
r_b	1.762×10^{-16}	ms^{-1}	Sum of products of rate constants	S120
r_{c1}	2.656×10^{-22}	ms^{-1}	Sum of products of rate constants	S120
r_{c2}	8.632×10^{-30}	ms^{-1}	Sum of products of rate constants	S120
r_1	2.077×10^{-18}		Sum of products of rate constants	S120
r_2	1.728×10^{-9}		Sum of products of rate constants	S120
r_3	1.059×10^{-26}		Sum of products of rate constants	S120
ρ^{res}	0.2	mM	Concentration of electron carriers (respiratory complexes I-III-IV)	S120
K_{res}	1.35×10^{18}		Equilibrium constant of respiration	S120
$\rho^{res(SDH)}$	0. 017	mM	Concentration of electron carriers (respiratory complexes II-III-IV)	S121
$\Delta\Psi_B$	50	mV	Phase boundary potential	S120-S122
g	0.85		Correction factor for voltage	S120-S122
K_i^{OAA}	0.15		Inhibition constant for OAA	S121
$K_{res(SDH)}$	5.765×10^{13}		Equilibrium constant of SDH	S121
$\rho^{res(ETF)}$	0. 75	mM	Concentration of electron carriers (respiratory complexes II-III-IV)	S122
$K_{res(FAD)}$	6.949×10^{16}		Equilibrium constant of $FADH_2$	S122
p_a	1.656×10^{-8}	ms^{-1}	Sum of products of rate constants	S123

Symbol	Value	Units	Description	Eq.
p_b	3.373×10^{-10}	ms^{-1}	Sum of products of rate constants	S123
p_{c1}	9.651×10^{-17}	ms^{-1}	Sum of products of rate constants	S123
p_{c2}	4.585×10^{-17}	ms^{-1}	Sum of products of rate constants	S123
p_1	1.346×10^{-4}		Sum of products of rate constants	S123
p_2	7.739×10^{-7}		Sum of products of rate constants	S123
p_3	6.65×10^{-15}		Sum of products of rate constants	S123
ρ^{F1}	1.5	mM	Concentration of F_1F_0-ATPase	S123
K_{eq}^{ATPase}	1.71×10^{6}		Equilibrium constant of ATP synthesis	S123
V_{maxANT}	3.15	mM ms^{-1}	Maximal rate of the ANT	S124
h^{ANT}	0.5		Fraction of $\Delta\Psi_B$	S124

3.3. Acid-base equilibria of adenine nucleotides and phosphate

$$[ATP^{4-}]_m = \frac{[ATP_{total}]_m}{\left(1+\frac{[H^+]_m}{K_{a,ATP}}+\frac{[Mg^{2+}]_m}{K_{Mg,ATP}}\right)} \quad \text{(S125)}$$

$$[HATP^{3-}]_m = \frac{[ATP^{4-}]_m[H^+]_m}{K_{a,ATP}}$$

$$[MgATP^{2-}]_m = \frac{[ATP^{4-}]_m[Mg^{2+}]_m}{K_{Mg,ATP}}$$

$$[ADP^{3-}]_m = \frac{[ADP_{total}]_m}{\left(1 + \frac{[H^+]_m}{K_{a,ADP}} + \frac{[Mg^{2+}]_m}{K_{Mg,ADP}}\right)}$$

$$[HADP^{2-}]_m = \frac{[ADP^{3-}]_m[H^+]_m}{K_{a,ADP}}$$

$$[MgADP^{-}]_m = \frac{[ADP^{3-}]_m[Mg^{2+}]_m}{K_{Mg,ADP}}$$

$$[H_2PO_4^-]_m = \frac{[Pi]_{total}}{1 + \frac{[H^+]_m}{K_{a,Pi}}} \tag{S126}$$

$$[HPO_4^{2-}]_m = \frac{[H_2PO_4^-]_m K_{a,Pi}}{[H^+]_m}$$

$$[ATP^{4-}]_i = \frac{[ATP_{total}]_i}{\left(1 + \frac{[H^+]_i}{K_{a,ATP}} + \frac{[Mg^{2+}]_i}{K_{Mg,ATP}}\right)} \tag{S127}$$

$$[ADP^{3-}]_i = \frac{[ADP_{total}]_i}{\left(1 + \frac{[H^+]_i}{K_{a,ADP}} + \frac{[Mg^{2+}]_i}{K_{Mg,ADP}}\right)}$$

$$[ATP_{total}] = [ATP^{4-}] + [HATP^{3-}] + [MgATP^{-}] \tag{S128}$$

$$[ATP_{free}] = [ATP^{4-}] + [HATP^{3-}]$$

$$[ADP_{free}] = [ADP^{3-}] + [HADP^{2-}]$$

$$[Pi_{total}] = [H_2Pi^{-}] + [H\ Pi^{2-}]$$

3.4. Polynomials for species undergoing acid-base equilibrium, ionic gradients, and conservation relations

$$P_{ATP} = 1 + \frac{[H^+]_m}{K_{a,ATP}} + \frac{[Mg^{2+}]_m}{K_{Mg,ATP}} \tag{S129}$$

$$P_{ADP} = 1 + \frac{[H^+]_m}{K_{a,ADP}} + \frac{[Mg^{2+}]_m}{K_{Mg,ADP}}$$

$$P_{Pi} = 1 + \frac{[H^+]_m}{K_{a,Pi}}$$

$$P_{SUC} = 1 + \frac{[H^+]_m}{K_{a,SUC}}$$

$$P_{H_2O} = 1 + \frac{[H^+]_m}{K_{a,H_2O}}$$

$$\Delta\mu_H = -2.303\frac{RT}{F}\Delta pH + \Delta\Psi_m \qquad \text{(S130)}$$

$$\Delta pH = pH_i - pH_m$$

$$\Delta\Psi_m = \Psi_i - \Psi_m$$

$$[NAD^+] = C_{PN} - [NADH] \qquad \text{(S131)}$$

$$[ATP_{total}] = C_A - [ADP_{total}] \qquad \text{(S132)}$$

Table I. Parameter values used in acid base equilibria and conservation relations

Symbol	Value	Units	Description	Eq.
$K_{a,ADP}$	4.17×10^{-7}		ADP dissociation constant	S125
$K_{a,ATP}$	3.31×10^{-7}		ATP dissociation constant	S125
$K_{a,Pi}$	1.78×10^{-7}		Pi dissociation constant	S126
$K_{Mg,ATP}$	6.46×10^{-5}		Mg^{2+}ATP dissociation constant	S125
$K_{Mg,ADP}$	5.62×10^{-4}		Mg^{2+}ADP dissociation constant	S125
$K_{a,SUC}$	6.3×10^{-6}		Ka of succinate dissociation constant	S129
K_{a,H_2O}	1×10^{-14}	M	Dissociation constant for water	S129
C_A	1.5	mM	Total sum of adenine nucleotides	S132
$[ADP]_i$	0.005-0.1	mM	Cytoplasmic ADP concentration	S127
C_{PN}	1.0	mM	Sum of pyridine nucleotides	S131

4. Appendix. Ionic fluxes rate equations

$$V_{uni} = V_{\max}^{uni} \frac{\frac{[Ca^{2+}]_i}{K_{trans}}\left(1+\frac{[Ca^{2+}]_i}{K_{trans}}\right)^3 \frac{2F\left(\Delta\Psi_m - \Delta\Psi^\circ\right)}{RT}}{\left(\left(1+\frac{[Ca^{2+}]_i}{K_{trans}}\right)^4 + \frac{L}{\left(1+\frac{[Ca^{2+}]_i}{K_{act}}\right)^{n_a}}\right)\left(1-e^{\left(\frac{-2F\left(\Delta\Psi_m-\Delta\Psi^\circ\right)}{RT}\right)}\right)} \quad \text{(S133)}$$

$$V_{NaCa} = V_{\max}^{NaCa} \frac{e^{\left(\frac{bF\left(\Delta\Psi_m-\Delta\Psi^\circ\right)}{RT}\right)} e^{\left(\ln\frac{[Ca^{2+}]_m}{[Ca^{2+}]_i}\right)}}{\left(1+\frac{K_{Na}}{[Na^+]_i}\right)^n \left(1+\frac{K_{Ca}}{[Ca^{2+}]_m}\right)} \quad \text{(S134)}$$

$$J_{NHE} = c_{NHE} \frac{\frac{\beta_1^+\beta_2^+ - \beta_1^-\beta_2^-}{\beta_1^+ + \beta_1^- + \beta_2^+ + \beta_2^-}}{1+10^{n_i(pH_i - pK_i)}} \quad \text{(S135)}$$

$$\beta_1^+ = \frac{k_1^+ K_{H\_NHE}[Na^+]_m}{K_{H\_NHE}[Na^+]_m + K_{H\_NHE}K_{Na\_NHE} + K_{Na\_NHE}[H^+]_m}$$

$$\beta_2^+ = \frac{k_2^+ K_{Na\_NHE}[H^+]_i}{K_{H\_NHE}[Na^+]_i + K_{H\_NHE}K_{Na\_NHE} + K_{Na\_NHE}[H^+]_i}$$

$$\beta_1^- = \frac{k_1^- K_{H\_NHE}[Na^+]_i}{K_{H\_NHE}[Na^+]_i + K_{H\_NHE}K_{Na\_NHE} + K_{Na\_NHE}[H^+]_i}$$

$$\beta_2^- = \frac{k_2^- K_{Na\_NHE}[H^+]_m}{K_{H\_NHE}[Na^+]_m + K_{H\_NHE}K_{Na\_NHE} + K_{Na\_NHE}[H^+]_m}$$

$$J_{PIC} = c_{PiC} \frac{V_{PIC,f}\frac{[HPO_4^{2-}]_i[OH^-]_m}{K_{Pi,i}K_{OH,m}} - V_{PIC,b}\frac{[HPO_4^{2-}]_m[OH^-]_i}{K_{Pi,m}K_{OH,i}}}{\left(\begin{array}{l}1+\frac{[HPO_4^{2-}]_i}{K_{Pi,i}} + \frac{[OH^-]_m}{K_{OH,m}} + \frac{[HPO_4^{2-}]_m}{K_{Pi,m}} + \ldots \\ \frac{[OH^-]_i}{K_{OH,i}} + \frac{[HPO_4^{2-}]_m[OH^-]_i}{K_{Pi,m}K_{OH,i}} + \frac{[HPO_4^{2-}]_i[OH^-]_m}{K_{Pi,i}K_{OH,m}}\end{array}\right)} \quad \text{(S136)}$$

$$V_{Hleak} = g_H\left(1+\frac{F_{FA}.C16CoA_c^{\,4}}{(C16CoA_c + C16CoA_{Ref})^4}\right)\Delta\mu_H \quad \text{(S137)}$$

Table J. Parameter values for the mitochondrial ion handling equations

Symbol	Value	Units	Description	Eq.
$V_{\max}^{uni}$	4.459×10^{-3}	mM ms^{-1}	V_{max} Ca^{2+} uniporter	S133
$\Delta\Psi^{\circ}$	91	mV	Offset membrane potential	S133
K_{act}	3.8×10^{-4}	mM	Activation constant	S133
K_{trans}	0.019	mM	K_d for translocated Ca^{2+}	S133
L	110.0		K_{eq} for conformational transitions in uniporter	S133
n_a	2.8		Uniporter activation cooperativity	S133
$V_{\max}^{NaCa}$	1.833×10^{-4}	mM ms^{-1}	V_{max} of Na^+/Ca^{2+} exchanger	S134
b	0.5		$\Delta\Psi_m$ dependence on Na^+/Ca^{2+} exchanger	S134
K_{Na}	9.4	mM	Exchanger Na^{2+} constant	S134
K_{Ca}	3.75×10^{-4}	mM	Exchanger Ca^{2+} constant	S134
n	3.0		Na^+/Ca^{2+} exchanger cooperativity	S134
δ_{Ca}	3×10^{-4}		Fraction of free $[Ca^{2+}]_m$	S55
δ_H	1×10^{-5}	dimensionless	mitochondria H^+ buffering capacity	S59
k_1^+	0.0252	ms^{-1}	NHE forward rate constant	S135
k_1^-	0.0429	ms^{-1}	NHE backward rate constant	S135
k_4^+	0.16	ms^{-1}	NHE forward rate constant	S135
k_4^-	0.0939	ms^{-1}	NHE backward rate constant	S135
K_{Na_NHE}	24	mM	Na^+ dissociation constant	S135
K_{H_NHE}	1.585×10^{-4}	mM	H^+ dissociation constant	S135

Symbol	Value	Units	Description	Eq.
pK_i	8.52		Proton inhibitory constant	S135
n_i_NHE	3		Hill coefficient for H^+ binding	S135
c_{NHE}	0.00785	mM	NHE concentration	S135
$K_{Pi,i}$	11.06	mM	Extra-matrix Pi binding constant	S136
$K_{Pi,m}$	11.06	mM	Mitochondrial matrix Pi binding constant	S136
$K_{OH,m}$	4.08×10^{-5}	mM	Mitochondrial matrix OH-binding constant	S136
$K_{OH,i}$	4.08×10^{-5}	mM	Extra-matrix OH- binding constant	S136
$V_{PIC,f}$	7.35×10^{-3}	mM ms^{-1}	Forward V_{max} of phosphate carrier	S136
$V_{PIC,b}$	7.35×10^{-3}	mM ms^{-1}	Backward V_{max} of phosphate carrier	S136
$[Pi]_i$	3.0	mM	Cytoplasmic phosphate concentration	S136
g_H	2.0×10^{-6}	mM ms^{-1} mV^{-1}	Ionic conductance of the inner membrane	S136
FFA	1.0×10^{2}		Leak-activation factor by PCoA	S137
$C16CoA_{Ref}$	35	μM	Reference concentration of PCoA	S137
$[H^+]_i$	1×10^{-4}	mM	Cytoplasmic H^+ concentration	S136
$[Na^+]_i$	10.0	mM	Cytoplasmic Na^+ concentration	S134, S135
$[Ca^{2+}]_i$	1×10^{-4}	mM	Cytoplasmic Ca^{2+} concentration	S133
C_{mito}	1.812×10^{-3}	mM mV^{-1}	Inner membrane capacitance	S57

5. Appendix. Redox balance, ROS generation, transport and scavenging

$$V_{IMAC} = \left(a + \frac{b}{1 + \frac{K_{cc}}{[O_2^{\bullet -}]_i}} \right) \left(GL + \frac{G_{max}}{1 + e^{\left(K\left(\Delta\Psi_m^b\right) + \Delta\Psi_m\right)}} \right) \Delta\Psi_m \quad \text{(S138)}$$

$$V_{ROS}^{Tr} = j \frac{V_{IMAC}}{\Delta\Psi_m} \left(-\Delta\Psi_m - \frac{RT}{F} \log\left(\frac{[O_2^{\bullet -}]_m}{[O_2^{\bullet -}]_i} \right) \right) \quad \text{(S139)}$$

Table K. Parameter values used in ROS transport

Symbol	Value	Units	Description	Eq.
a	1×10^{-3}	dimensionless	Basal IMAC conductance	S138
b	1×10^{4}	dimensionless	Activation factor by cytoplasmic $O_2^{\cdot -}$	S138
K_{cc}	1×10^{-2}	mM	Activation constant by cytoplasmic $O_2^{\cdot -}$	S138
GL	3.5×10^{-8}		IMAC integral conductance	S138
G_{max}	3.9085×10^{-6}		IMAC leak conductance at saturation	S138
K	7.0×10^{-2}	mV^{-1}	Steepness factor	S138
$\Delta\Psi_m^b$	4	mV	Potential at half saturation	S138
j	0.1	dimensionless	Fraction of IMAC conductance	S139
$\frac{RT}{F}$	26.730818	mV		

$$V_{MnSOD} = \frac{2\, k_{SOD}^1\, k_{SOD}^5 \left(k_{SOD}^1 + k_{SOD}^3 \left(1 + \frac{[H_2O_2]_m}{K_i^{H_2O_2}} \right) \right) E_{MnSOD}^T [O_2^{\bullet -}]_m}{k_{SOD}^5 \left(2\, k_{SOD}^1 + k_{SOD}^3 \left(1 + \frac{[H_2O_2]_m}{K_i^{H_2O_2}} \right) \right) + [O_2^{\bullet -}]_m\, k_{SOD}^1\, k_{SOD}^3 \left(1 + \frac{[H_2O_2]_m}{K_i^{H_2O_2}} \right)} \quad \text{(S140)}$$

$$V_{\mathrm{CuZnSOD}} = \frac{2\ k^{1}_{\mathrm{SOD}}\ k^{5}_{\mathrm{SOD}} \left(k^{1}_{\mathrm{SOD}} + k^{3}_{\mathrm{SOD}} \left(1 + \frac{[\mathrm{H_2O_2}]_{\mathrm{i}}}{\mathrm{K}_{\mathrm{i}}^{\mathrm{H_2O_2}}} \right) \right) \mathrm{E}^{\mathrm{T}}_{\mathrm{CuZnSOD}} [\mathrm{O}_2^{\bullet -}]_i}{k^{5}_{\mathrm{SOD}} \left(2\ k^{1}_{\mathrm{SOD}} + k^{3}_{\mathrm{SOD}} \left(1 + \frac{[\mathrm{H_2O_2}]_{\mathrm{i}}}{\mathrm{K}_{\mathrm{i}}^{\mathrm{H_2O_2}}} \right) \right) + [\mathrm{O}_2^{\bullet -}]_i\ k^{1}_{\mathrm{SOD}}\ k^{3}_{\mathrm{SOD}} \left(1 + \frac{[\mathrm{H_2O_2}]_i}{\mathrm{K}_{\mathrm{i}}^{\mathrm{H_2O_2}}} \right)} \tag{S141}$$

$$V_{diff_{H_2O_2}} = c_{diff_{H_2O_2}} \left([H_2O_2]_m - [H_2O_2]_i \right) \tag{S142}$$

$$V_{GPX_m} = \frac{E_T^{GPXm} [\mathrm{H_2O_2}]_{\mathrm{m}} [\mathrm{GSH}]_{\mathrm{m}}}{\Phi_1 [\mathrm{GSH}]_{\mathrm{m}} + \Phi_2 [\mathrm{H_2O_2}]_{\mathrm{m}}} \tag{S143}$$

$$V_{GPX_i} = \frac{E_T^{GPXi} [\mathrm{H_2O_2}]_{\mathrm{i}} [\mathrm{GSH}]_{\mathrm{i}}}{\Phi_1 [\mathrm{GSH}]_{\mathrm{i}} + \Phi_2 [\mathrm{H_2O_2}]_{\mathrm{i}}} \tag{S144}$$

$$V_{GR_m} = \frac{k^{1}_{\mathrm{GR}}\ \mathrm{E}_T^{\mathrm{GRm}}}{1 + \frac{K_M^{\mathrm{GSSG}}}{[\mathrm{GSSG}]} + \frac{K_M^{\mathrm{NADPH}}}{[\mathrm{NADPH}]_{\mathrm{m}}} + \frac{K_M^{\mathrm{GSSG}}}{[GSSG]_m} \frac{K_M^{\mathrm{NADPH}}}{[\mathrm{NADPH}]_{\mathrm{m}}}} \tag{S145}$$

$$V_{GR_i} = \frac{k^{1}_{\mathrm{GR}}\ \mathrm{E}_T^{\mathrm{GRi}}}{1 + \frac{K_M^{\mathrm{GSSG}}}{\mathrm{V}_{GSS}} + \frac{K_M^{\mathrm{NADPH}}}{[\mathrm{NADPH}]_{\mathrm{i}}} + \frac{K_M^{\mathrm{GSSG}}}{[GSSG]_i} \frac{K_M^{\mathrm{NADPH}}}{[\mathrm{NADPH}]_{\mathrm{i}}}} \tag{S146}$$

$$V_{GRX_m} = \frac{k_{grx_m} K_{eq}^{GRX} \left([GSH]_m \right)^2 GrxT\ [PSSG]_m}{\left([\mathrm{GSSG}]_{\mathrm{m}} + K_{eq}^{GRX} \left([GSH]_m \right)^2 \right) \left(\frac{K_{eq}^{GRX} \left([GSH]_m \right)^2 GrxT}{[\mathrm{GSSG}]_{\mathrm{m}} + K_{eq}^{GRX} \left([GSH]_m \right)^2} + K_m^{Grx} \right) \left([\mathrm{PSSG}]_{\mathrm{m}} + K_m^{PSSG} \right)} \tag{S147}$$

$$V_{GRX_i} = \frac{k_{grx_i} K_{eq}^{GRX} \left([GSH]_i \right)^2 GrxT\ [PSSG]_i}{\left(\mathrm{V_{GSS}} + K_{eq}^{GRX} \left([GSH]_i \right)^2 \right) \left(\frac{K_{eq}^{GRX} \left([GSH]_i \right)^2 GrxT}{[GSSG]_i + K_{eq}^{GRX} \left([GSH]_i \right)^2} + K_m^{Grx} \right) \left([\mathrm{PSSG}]_{\mathrm{i}} + K_m^{PSSG} \right)} \tag{S148}$$

$$V_{PSSG_m} = \frac{k^{1}_{\mathrm{PSH}}\ \mathrm{E}_T^{\mathrm{PSH}}\ (PSSGT - [PSSG]_m)}{\left(1 + \frac{K_M^{GSH}}{[GSH]_m} \right) \left(1 + \frac{[H_2O_2]_m}{K_{act}^{H2O2}} \right)} \tag{S149}$$

$$V_{PSSG_i} = \frac{k^1_{\mathrm{PSH}}\ \mathrm{E}_T^{\mathrm{PSH}}\ (PSSGT - \left[PSSG\right]_i)}{\left(1 + \frac{K_M^{GSH}}{[GSH]_i}\right)\left(1 + \frac{[H_2O_2]_i}{K_{act}^{H2O2}}\right)} \quad \text{(S150)}$$

$$\mathrm{G}_T = \mathrm{G}_T - \left[\mathrm{GSH}\right]_{\mathrm{m}} - \left[\mathrm{GSH}\right]_i - 2\left[\mathrm{GSSG}\right] - \left[\mathrm{PSSG}\right]_{\mathrm{m}} - \left[\mathrm{PSSG}\right]_{\mathrm{i}} - 2\left[GSSG\right]_i \quad \text{(S151)}$$

$$\left[\mathrm{GSSG}\right]_i = 0.5\left(\mathrm{G}_T - [\mathrm{GSH}]_{\mathrm{m}} - [\mathrm{GSH}]_i - 2[\mathrm{GSSG}]_{\mathrm{m}} - [\mathrm{PSSG}]_{\mathrm{m}} - [\mathrm{PSSG}]_{\mathrm{i}}\right) \quad \text{(S152)}$$

$$V_{GST} = c_{GST}\,\frac{\left(\left[GSH\right]_i - \left[GSH\right]_m\right)}{\left[GSH\right]_i + k_{0.5}^{GST}} \quad \text{(S153)}$$

$$V_{TxPX_m} = \frac{E_T^{Prx3m}[H_2O_2]_m\ [TrxSH_2]_m}{\Phi_{1Prx}[TrxSH_2]_m + \Phi_{2Prx}[H_2O_2]_m} \quad \text{(S154)}$$

$$V_{TxPX_i} = \frac{E_T^{Prxi}[\mathrm{H_2O_2}]_{\mathrm{i}}\,[\mathrm{TrxSH_2}]_{\mathrm{i}}}{\Phi_{1Prx}[\mathrm{TrxSH_2}]_{\mathrm{i}} + \Phi_{2Prx}[\mathrm{H_2O_2}]_i} \quad \text{(S155)}$$

$$V_{TxR_m} = \frac{k^1_{\mathrm{Trx}R}\ \mathrm{E}_T^{Tr\mathrm{xR2m}}}{1 + \frac{K_M^{\mathrm{TrxSS}}}{[TrxSS]_m} + \frac{K_{Mtrx}^{\mathrm{NADPH}}}{[NADPH]_m} + \frac{K_M^{\mathrm{TrxSS}}}{[TrxSS]_m}\frac{K_{Mtrx}^{\mathrm{NADPH}}}{[NADPH]_m}} \quad \text{(S156)}$$

$$V_{TxR_i} = \frac{k^1_{\mathrm{TrxR}}\ \mathrm{E}_T^{\mathrm{TrxRi}}}{1 + \frac{K_M^{\mathrm{TrxSS}}}{[TrxSS]_i} + \frac{K_{Mtrx}^{\mathrm{NADPH}}}{[NADPH]_i} + \frac{K_M^{\mathrm{TrxSS}}}{[TrxSS]_i}\frac{K_{Mtrx}^{\mathrm{NADPH}}}{[NADPH]_i}} \quad \text{(S157)}$$

$$[\mathrm{TrxSS}]_{\mathrm{m}} = TrxT_m - [\mathrm{TrxSH_2}]_{\mathrm{m}} \quad \text{(S158)}$$

$$[\mathrm{TrxSS}]_{\mathrm{i}} = TrxT_i - [\mathrm{TrxSH_2}]_{\mathrm{i}} \quad \text{(S159)}$$

$$V_{CAT} = 2k^1_{CAT}E^T_{CAT}[\mathrm{H_2O_2}]_{\mathrm{i}}\,e^{-fr[\mathrm{H_2O_2}]_{\mathrm{i}}} \quad \text{(S160)}$$

Table L. Parameter values corresponding to ROS production and scavenging

Symbol	Value	Units	Description	Eq
k_{SOD}^{1}	1.2×10^{3}	$mM^{-1}ms^{-1}$	SOD second-order rate constant	S140,S141
k_{SOD}^{3}	24	$mM^{-1}ms^{-1}$	SOD second-order rate constant	S140,S141
k_{SOD}^{5}	2.4×10^{-4}	ms^{-1}	SOD first-order rate constant	S140,S141
$K_{i}^{H_2O_2}$	0.5	mM	Inhibition constant for H_2O_2	S140,S141
E_{MnSOD}^{T}	3.0×10^{-3}	mM	MnSOD matrix concentration	S140
$E_{CuZnSOD}^{T}$	3.0×10^{-3}	mM	Cu,ZnSOD concentration	S141
$c_{diff_{H_2O_2}}$	2×10^{-4}	ms^{-1}	Diffusion constant for H_2O_2	S142
Φ_1	5.0×10^{-3}	mM ms	GPX activity constant	S143,S144
Φ_2	0.75	mM ms	GPX activity constant	S143,S144
E_T^{GPXm}	1.0×10^{-4}	mM	GPX matrix concentration	S143
E_T^{GPXi}	5.0×10^{-5}	mM	GPX extra-matrix concentration	S144
k_{GR}^{1}	2.5×10^{-3}	ms^{-1}	Catalytic constant of glutathione reductase (GR)	S145,S146
E_{T}^{GRm}	9.0×10^{-4}	mM	GR matrix concentration	S145
E_{T}^{GRi}	9.0×10^{-4}	mM	GR extra-matrix concentration	S146
K_{M}^{NADPH}	0.015	mM	GR Michaelis constant for NADPH	S145,S35
K_{M}^{GSSG}	0.06	mM	GR Michaelis constant for GSSG	S145,S146
$[NADPH]_i$	7.5×10^{-2}	mM	Extra-matrix NADPH concentration	S145,S146
G_T	6	mM	Total pool of glutathione	S151,S152
k_{grx_m}	3.6×10^{-4}	$mM\ s^{-1}$	Rate constant of mitochondrial matrix glutaredoxin (GRX) reaction	S147
k_{grx_i}	3.6×10^{-4}	$mM\ s^{-1}$	GRX extra-matrix rate constant	S148
K_{eq}^{GRX}	1.37×10^{-3}	mM^{-1}	GRX equilibrium constant	S147,S148

Symbol	Value	Units	Description	Eq
K_m^{Grx}	0.01	mM	GRX Michaelis constant for GSH	S147,S148
K_m^{PSSG}	0.0005	mM	Michaelis constant for glutathionylated proteins	S147,S148
k_{PSH}^{1}	0.64	ms^{-1}	Rate constant of protein glutathionylation	S149
$\mathrm{E}_T^{\mathrm{PSH}}$	8×10^{-4}	mM	Concentration of proteins that can become glutathionylated	S149,S150
K_M^{GSH}	0.75	mM	Michaelis constant of GSH for glutathionylation	S149,S150
K_{act}^{H2O2}	1×10^{-3}	mM	Activation constant of H_2O_2 for protein glutathionylation	S149,S150
$GrxT$	0.002	mM	Glutaredoxin concentration	S149,S150
c_{GST}	1.5×10^{-8}	ms^{-1}	Rate constant of glutathione transporter	S153
$k_{0.5}^{GST}$	2.6	mM	Transport association constant of GSH	S153
E_T^{Prx3m}	3.0×10^{-3}	mM	Mitochondrial matrix concentration of Trx peroxidase (Prx)	S154
E_T^{Prx3i}	0.1	mM	Prx extra-matrix concentration	S155
Φ_{1Prx}	3.83	mM ms	Constant for TxPX activity	S154,S155
Φ_{2Prx}	1.85	mM ms	Constant for TxPX activity	S154,S155
$\mathrm{E}_T^{Trx\mathrm{R2m}}$	3.5×10^{-4}	mM	Mitochondrial matrix concentration of thioredoxin reductase2 (TrxR2)	S156
$\mathrm{E}_T^{Trx\mathrm{R}i}$	3.5×10^{-4}	mM	TrxR extra-matrix concentration	S157
K_M^{TrxSS}	0.035	mM	TrxR Michaelis constant for oxidized Trx [Trx(SS)]	S156,S157
$\mathrm{K}_{Mtrx}^{NADPH}$	0.012	mM	Trx Michaelis constant for NADPH	S156,S157
k_{TrxR}^{1}	22.7×10^{-3}	ms^{-1}	TrxR rate constant	S156,S157
$TrxT_m$	0.025	mM	Total pool of mitochondrial matrix thioredoxin	S158
$TrxT_i$	0.05	mM	Total pool of extra-matrix thioredoxin	S159

Symbol	Value	Units	Description	Eq
k_{CAT}^{1}	17	$mM^{-1}ms^{-1}$	Rate constant of catalase (CAT)	S160
E_{CAT}^{T}	1.0×10^{-6}	mM	CAT extra-matrix concentration	S160
fr	5.0×10^{-2}	mM^{-1}	CAT hydrogen peroxide inhibition factor	S160

6. Appendix. Mitochondrial NADPH reduction

$$NADPm = C_{NADPm} - \left[NADPH\right]_m \quad \text{(S161)}$$

$$V_{IDP\_NADP} = \left(1 + \frac{\left[H^+\right]_m}{k_{m\_IDP}^{H+}}\right) \left(\begin{array}{l} 1 + \frac{\left[ISOC\right]}{k_{m\_IDP}^{ISOC}} + \frac{NADP_m}{k_{m\_IDP}^{NADP}}\left(1 + \frac{k_{i\_IDP}^{NADP}}{NADP_m}\right) + \frac{\left[aKG\right]}{k_{m\_IDP}^{aKG}} + \frac{\left[NADPH_m\right]}{k_{m\_IDP}^{NADPH}} + \ldots \\ \ldots \frac{\left[ISOC\right]}{k_{m\_IDP}^{ISOC}} \frac{NADP_m}{k_{m\_IDP}^{NADP}}\left(1 + \frac{k_{i\_IDP}^{NADP}}{NADP_m}\right) + \frac{\left[aKG\right]}{k_{m\_IDP}^{aKG}} \frac{\left[NADPH_m\right]}{k_{m\_IDP}^{NADPH}} + \ldots \\ \ldots \frac{\left[ISOC\right]}{k_{m\_IDP}^{ISOC}} \frac{\left[NADPH_m\right]}{k_{m\_IDP}^{NADPH}} + \frac{\left[aKG\right]}{k_{m\_IDP}^{aKG}} \frac{NADP_m}{k_{m\_IDP}^{NADP}}\left(1 + \frac{k_{i\_IDP}^{NADP}}{NADP_m}\right) \end{array} \right) \quad \text{(S162)}$$

$$V_{IDH_{NADP}} = \frac{V_f^{IDH} \frac{k\left[ISOC\right]}{k_{m\_IDP}^{ISOC}} \frac{NADP_m}{k_{m\_IDP}^{NADP}}\left(1 + \frac{k_{i\_IDP}^{NADP}}{VNADH_m}\right) - V_b^{IDH} \frac{\left[aKG\right]}{k_{m\_IDP}^{aKG}} \frac{\left[NADP_m\right]}{k_{m\_IDP}^{NADPH}}}{V_{IDP\_NADP}} \quad \text{(S163)}$$

$$V_{THDen} = 1 + \frac{\left[NADH_m\right]}{k_{m\_THD}^{NADHm}} + \frac{NAD}{k_{m\_THD}^{NAD}} + \frac{NADP_m}{k_{m\_THD}^{NADP}} + \frac{\left[NADPH_m\right]}{k_{m\_THD}^{NADPH}} + \frac{\left[NADH_m\right]}{k_{m\_THD}^{NADHm}} \frac{NADP_m}{k_{m\_THD}^{NADP}} e^{(F/10RT).\Delta\mu_H} + \frac{\left[NADPH_m\right]}{k_{m\_THD}^{NADPH}} \frac{\left[NADH_m\right]}{k_{m\_THD}^{NADHm}} e^{(1-(F/10RT).\Delta\mu_H)} + \frac{NAD}{k_{m\_THD}^{NAD}} \frac{NADP_m}{k_{m\_THD}^{NADP}} e^{(F/10RT).\Delta\mu_H} . e^{(1-(F/10RT).\Delta\mu_H)} + \frac{\left[NADH_m\right]}{k_{m\_THD}^{NADHm}} \frac{\left[NADPH_m\right]}{k_{m\_THD}^{NADPH}} \quad \text{(S164)}$$

$$V_{THD} = \frac{E_T^{THD} . k_a^{THD} \frac{\left[NADH\right]_m}{k_{m\_THD}^{NADHm}} \frac{NADP_m}{k_{m\_THD}^{NADP}} e^{(F/10RT).\Delta\mu_H} - E_T^{THD} . k_b^{THD} \frac{NAD}{k_{m\_THD}^{NAD}} \frac{\left[NADPH_m\right]}{k_{m\_THD}^{NADPH}} e^{(1-(F/10RT).\Delta\mu_H)}}{V_{THDen}} \quad \text{(S165)}$$

Table M. Parameter values used in mitochondrial NADPH handling

Symbol	Value	Units	Description	Eq.
c_{NADPm}	0.1	mM	Sum of NADPH plus $NADP^{+}$	S161
$k_{m\_IDP}^{H+}$	0.5	mM	Dissociation constant for H^{+} of isocitrate dehydrogenase2 (IDH2)	S162
$k_{m\_IDP}^{ISOC}$	3.9×10^{-3}	mM	IDH2 Michaelis constant for ISOC	S162,S163
$k_{m\_IDP}^{NADP}$	6.7×10^{-3}	mM	IDH2 Michaelis constant for NADP	S162,S163
$k_{i\_IDP}^{NADP}$	2×10^{-6}	mM	IDH2 inhibition constant for NADP	S162,S163
$k_{m\_IDP}^{NADPH}$	1.2×10^{-2}	mM	IDH2 Michaelis constant for NADPH	S162,S163
$k_{m\_IDP}^{aKG}$	0.51	mM	IDH2 Michaelis constant for αKG	S162,S163
V_{f}^{IDH}	8.7×10-5	mM ms^{-1}	Maximal rate of IDH2 in the forward direction	S163
V_{fb}^{IDH}	5.45×10^{-6}	mM ms^{-1}	Maximal rate of IDH2 in the reverse direction	S163
$k_{m\_THD}^{NADPH}$	0.02	mM	Michaelis constant for NADPH in transhydrogenase (THD)	S164,S165
$k_{m\_THD}^{NADHm}$	0.01	mM	THD Michaelis constant for NADH	S164,S165
$k_{m\_THD}^{NAD}$	0.125	mM	THD Michaelis constant for NAD	S164,S165
$k_{m\_THD}^{NADP}$	0.017	mM	THD Michaelis constant for NADP	S164,S165
E_{T}^{THD}	1.187×10^{-5}	mM	THD enzyme concentration	S165
k_{a}^{THD}	1.17474	ms^{-1}	THD forward catalytic constant	S165
k_{b}^{THD}	10	ms^{-1}	THD reverse catalytic constant	S165

7. Appendix. Initial Conditions

Table N. State variables initial values

Symbol	Value	Units	Description
$[Ca^{2+}]_m$	2.64×10^{-5}	mM	Mitochondrial matrix Ca^{2+}
$[ADP]_m$	0.0049	mM	Mitochondrial matrix ADP
$\Delta\Psi_m$	193.0	mV	Mitochondrial membrane potential
[NADH]	0.746	mM	Mitochondrial matrix NADH
$[H^+]_m$	8.1×10^{-5}	mM	Mitochondrial matrix H^+
$[Pi]_m$	11.05	mM	Mitochondrial matrix Pi
[ISOC]	0.0315	mM	Isocitrate
[αKG]	0.0383	mM	α-ketoglutarate
[SCoA]	0.229	mM	Succinyl CoA
[Suc]	0.065	mM	Succinate
[FUM]	0.2244	mM	Fumarate
[MAL]	0.2203	mM	Malate
[OAA]	0.185	mM	Oxalacetate
$[C16Carn]_i$	0.807	mM	Cytosolic Palmitoyl Carnitine
$[C16Carn]_m$	1.92	mM	Mitochondrial Palmitoyl Carnitine
$[C16CoA]_m$	0.527	mM	Palmitoyl CoA
$[C16enoylCoA]_m$	9.9×10^{-4}	mM	C16 trans hexadec2 enoylCoA
$[C16OHCoA]_m$	1.1×10^{-4}	mM	3-hydroxy palmitoylCoA
$[C16ketoCoA]_m$	3.3×10^{-4}	mM	3-keto palmitoylCoA
$[C14CoA]_m$	0.0905	mM	Myristoyl CoA
$[C14enoylCoA]_m$	1.6×10^{-4}	mM	Trans tetradec 2 enoyl CoA
$[C14OHCoA]_m$	4.6×10^{-5}	mM	3-hydroxy myristoylCoA
$[C14ketoCoA]_m$	1.3×10^{-4}	mM	3-keto myristoylCoA
$[C12CoA]_m$	0.0117	mM	Lauroyl CoA
$[C12enoylCoA]_m$	2.2×10^{-5}	mM	Trans-dodec-2-enoyl CoA
$[C12OHCoA]_m$	4.0×10^{-5}	mM	3-hydroxy-lauroyl CoA
$[C12ketoCoA]_m$	4.2×10^{-5}	mM	3-keto lauroyl CoA

Symbol	Value	Units	Description
$[C10CoA]_m$	0.0061	mM	Decanoyl CoA
$[C10enoylCoA]_m$	1.1×10^{-5}	mM	Trans dec-2-enoyl CoA
[C10OHCoA]m	3.1×10^{-5}	mM	3-hydroxy decanoyl CoA
$[C10ketoCoA]_m$	1.8×10^{-5}	mM	3-keto decanoyl CoA
$[C8CoA]_m$	0.0031	mM	Octanoyl CoA
$[C8enoylCoA]_m$	5.9×10^{-6}	mM	Trans oct-2-enoyl CoA
$[C8OHCoA]_m$	1.4×10^{-5}	mM	3-hydroxy octanoyl CoA
$[C8ketoCoA]_m$	9.2×10^{-6}	mM	3-keto octanoyl CoA
$[C6CoA]_m$	0.0016	mM	Hexanoyl CoA
$[C6enoylCoA]_m$	2.9×10^{-6}	mM	Trans hex-2-enoyl CoA
$[C6OHCoA]_m$	1.4×10^{-5}	mM	3-hydroxy hexanoyl CoA
$[C6ketoCoA]_m$	9.2×10^{-6}	mM	3-keto hexanoyl CoA
$[C4CoA]_m$	0.0078	mM	Butanoyl CoA
$[C4enoylCoA]_m$	1.5×10^{-5}	mM	Trans but-2-enoyl CoA
$[C4OHCoA]_m$	4.9×10^{-5}	mM	3-hydroxy butanoyl CoA
$[C4ketoCoA]_m$	3.4×10^{-5}	mM	3-keto butanoyl CoA
$[AcCoA]_m$	0.0024	mM	Mitochondrial AcetylCoA
$[FADH_2]_m$	0.6	mM	Mitochondrial FADH
$[NADPH]_m$	0.097	mM	Mitochondrial NADPH
$[Na^+]_m$	0.0985	mM	Mitochondrial matrix Na^+
$[O_2^{\bullet-}]_m$	5.5×10^{-7}	mM	Matrix Superoxide
$[O_2^{\bullet-}]_i$	1.1×10^{-10}	mM	Extra-matrix Superoxide
$[H_2O_2]_m$	9.0×10^{-5}	mM	Matrix hydrogen peroxide
$[H_2O_2]_i$	7.7×10^{-8}	mM	Extra-matrix Hydrogen peroxide
$[GSH]_m$	1.21	mM	Mitochondrial matrix GSH
$[GSH]_i$	1.21	mM	Extra-matrix GSH
$[GSSG]_m$	1.76	mM	Mitochondrial matrix GSSG
$[TrxSH_2]_m$	0.0243	mM	Mitochondrial matrix $TrxSH_2$
$[TrxSH_2]_i$	0.0499	mM	Extra-matrix $TrxSH_2$

Symbol	Value	Units	Description
$[PSSG]_m$	8.4×10^{-4}	mM	Mitochondrial matrix PSSG
$[PSSG]_i$	3.6×10^{-5}	mM	Extra-matrix PSSG

References

1. van Eunen K, Simons SM, Gerding A, Bleeker A, den Besten G, et al. (2013) Biochemical competition makes fatty-acid beta-oxidation vulnerable to substrate overload. PLoS Comput Biol 9: e1003186.
2. Kembro JM, Aon MA, Winslow RL, O'Rourke B, Cortassa S (2013) Integrating mitochondrial energetics, redox and ROS metabolic networks: a two-compartment model. Biophys J 104: 332-343.
3. Mazumder PK, O'Neill BT, Roberts MW, Buchanan J, Yun UJ, et al. (2004) Impaired cardiac efficiency and increased fatty acid oxidation in insulin-resistant ob/ob mouse hearts. Diabetes 53: 2366-2374.

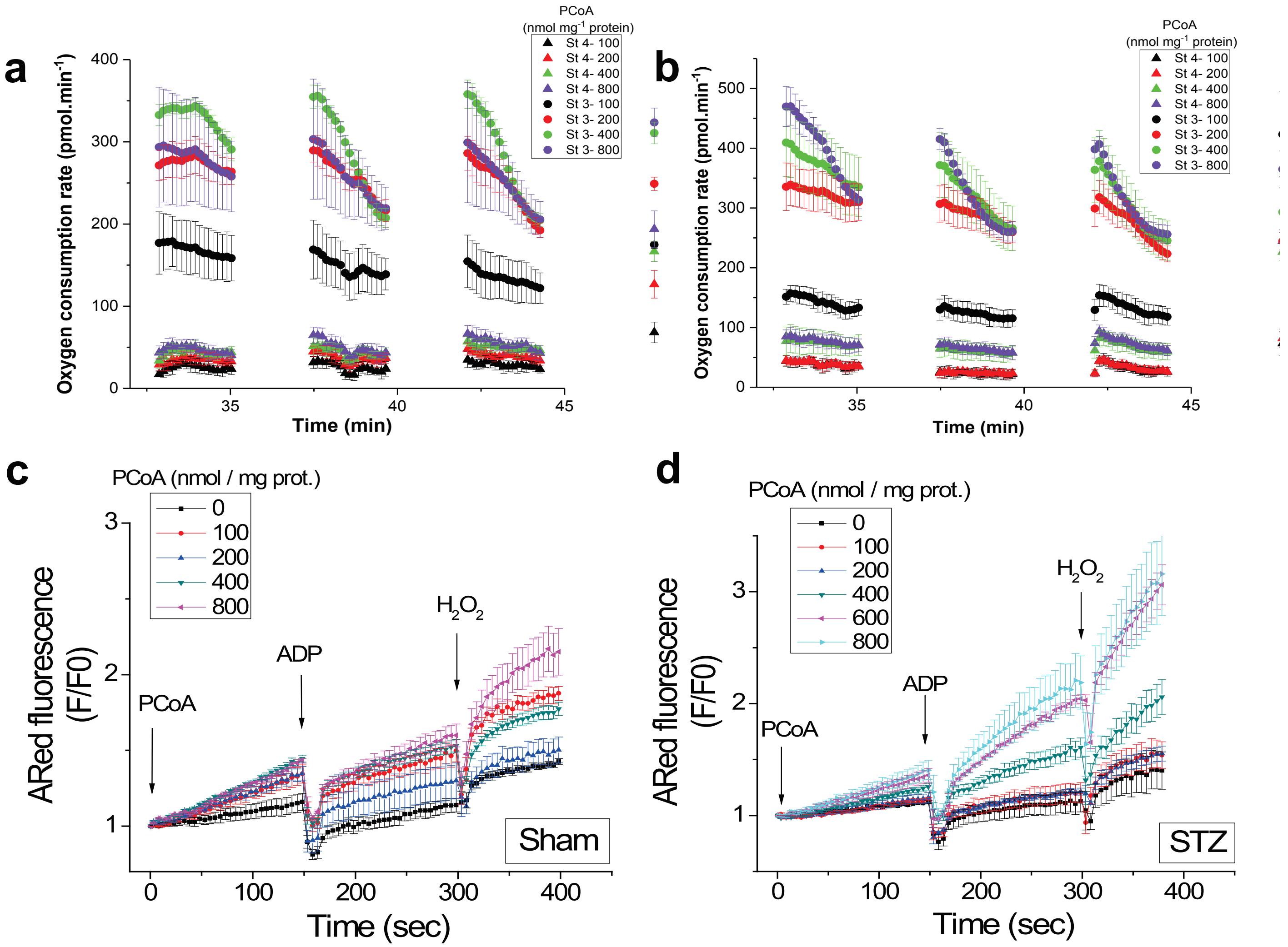

Figure C. Representative traces of the time courses of oxygen consumption flux and H2O2 emission from heart mitochondria of diabetic and control guinea pigs consuming PCoA. Panels a and b display the output of the Seahorse oxygen consumption rate (OCR) averaged over 4-6 technical repeats in an experiment in which PCoA was provided at the levels indicated in the legend as electron donor in the presence of 0.5 mM carnitine and 0.5 mM Malate to enable β-oxidation conditions under states 4 and 3 conditions (0.5 mM ADP). The first 6-8 points of OCR were averaged and normalized per mitochondrial protein to obtain the V_{O2} values shown in Fig. 2 of the main text. Panel a displays the results obtained with mitochondria isolated from a Sham animal while panel b corresponds to a STZ-treated guinea pig.

The rate of H_2O_2 emission from mitochondria monitored with Amplex Red (ARed) was recorded in a Molecular Devices FlexStation as described under Material and Methods. Panel c shows the average of 4 technical repeats from Sham heart mitochondria, while panel d corresponds to mitochondria isolated from hearts of STZ-treated guinea pigs. At the end of the recording a known amount of H_2O_2 was added to calibrate the signal. The slopes of the ARed fluorescence are determined as the V_{H2O2} values reported in Fig. 2 of the main text.

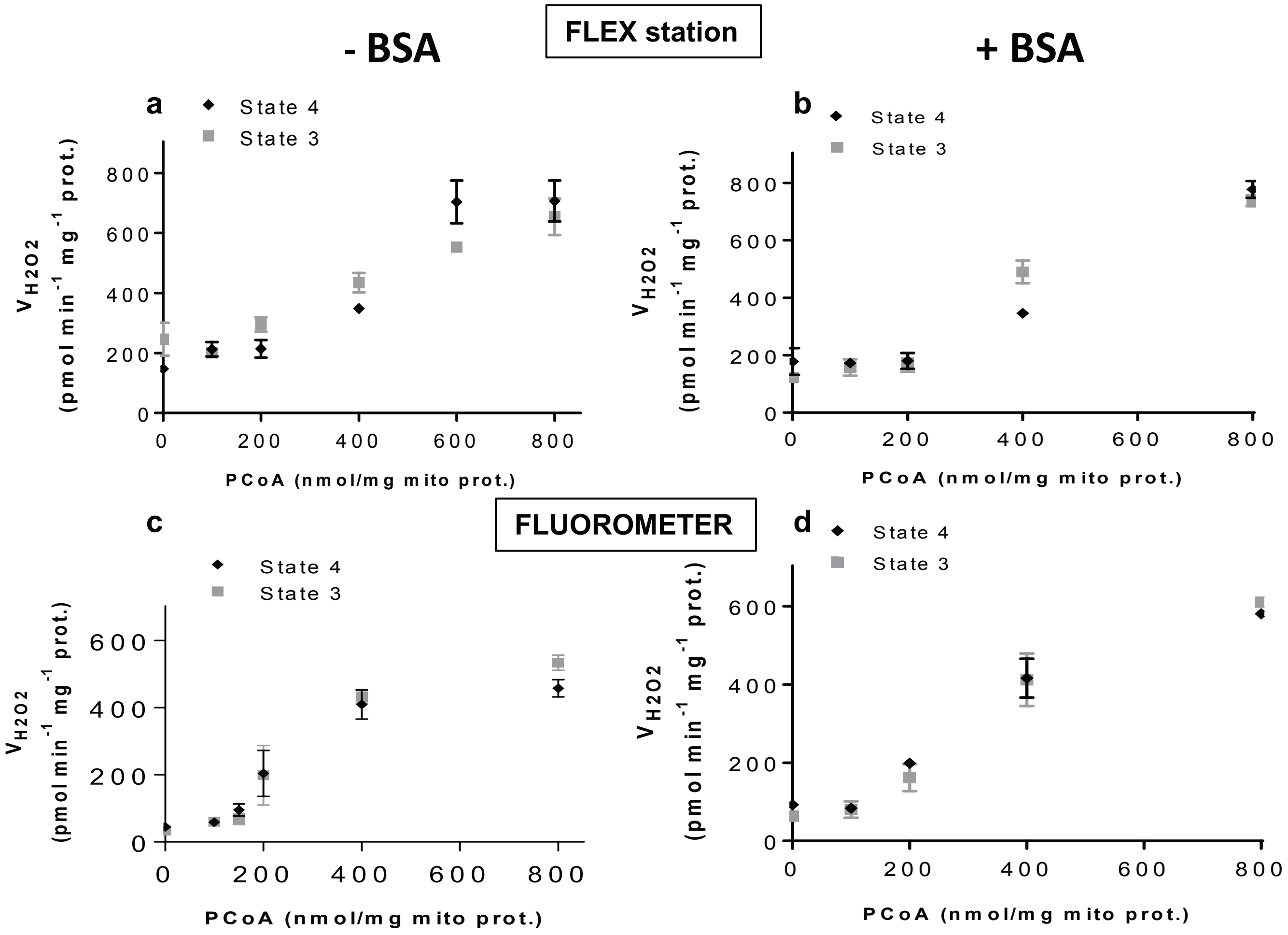

Figure D. Control of the effect of Bovine Serum Albumin (BSA) on the rate of H_2O_2 emission from mitochondria as a function of the PCoA concentration. The Amplex Red measurements were performed in two different setups (96 well plate on a Molecular Devices Flex Station (a, b) and a single cuvette PTI fluorometer (c, d) in the absence (a, c) or presence of 0.2% fatty acid-free BSA, and both with or without 0.5mM ADP (state 3 or state 4 respiration, respectively). We designed these experiments to control for the possible BSA sequestering of part of the PCoA thus decreasing its availability for mitochondria.

MATLAB code for the model

```
function dydt = FAO_007_CodePLOSCompBiol_2017(t,kmrgd) %

ADP_cyto=0.05;

RhoF1=1.5;

VmANT=3.15;

Pi_cyto=3;

GLU=1.0E-3;

RhoREN=1.0E-1;

RhoSDH=1.7E-2;

gh=1.0E-6;

ketf=6.949E16;

RhoETF=0.75;

kresf=5.765E13;

KeqF1=1.71E6;

Catp=1.01;      %total cytosolic adenine nucleotides

Cai=1.0E-4;

beta_matr=1e-5;     %Mitochondrial buffering capacity

Cm=1.5;             %total mitochondrial adenine nucleotides

Conc_NHE=0.00785; %concentration NHE

%TCA cycle enzyme activities

KcsicoA=0.056;

KcsacCoA=4.4E-3;

KcsiacCoA=4.4E-3;

KcsOaa=3.3E-3;

KCS=0.00023523;

kfACO=0.00011688;

kfidh=11.88;

kfKG=0.013245;

KfSL=0.0028;

kfFH=0.0083;

kfMDH=0.1242;

kfAAT=0.0214;
```

```
KSLeq=3.115;

kh_1=1E-5;

kh_2=9E-4;

kh_1a=4E-5;

kh_2a=7E-5;

khm1=1.131E-5;

khm2=2.67E1;

khm3=6.68E-9;

khm4=5.62E-6;

kmoff=3.99E-2;

Mg=0.4;

KkgCa= 1.5E-4;

VmPiC=4.9;

Cimat=1.812E-3;

VNai=10.0;

Vmuni= 0.004459;

VmNaCa=18.33e-5;

fCam = 3.0E-4;

ATPm = Cm - kmrgd(2);

%% Acid-base dissociation equilibria and polynomia

H_mito_1000= kmrgd(5)/1000;

Mg_1000=Mg/1000;

Hi=1.0E-4;

KaidADP=6.2E-1;

KaidCa=5.0E-4;

KaPi=1.78E-7;

KaATP=3.31E-7;

KMgATP=6.46E-5;

KaADP=4.17E-7;

KMgADP= 5.62E-4;

KaSUC=6.3E-6;

KaH2O=1.0E-14;
```

```
%
ATP4 = ATPm / ( 1+ H_mito_1000/KaATP + Mg_1000/ KMgATP);
ATPMg= ATP4 * Mg_1000 / KMgATP;
HATP=  ATP4 * H_mito_1000 / KaATP ;
ADP3=  kmrgd(2) / ( 1+ H_mito_1000/KaADP + Mg_1000/ KMgADP);
% ADPMg= ADP3 * Mg_1000/KMgADP;
HADP=  ADP3 * H_mito_1000/KaADP ;
H2Pi = kmrgd(6)/(1+ KaPi/H_mito_1000);
% HPi= H2Pi*KaPi/(H_mito_1000);
ATP4_cyto= (Catp-ADP_cyto) / (1+ Hi/1000/KaATP + 1/1000/KMgATP);
ADP3_cyto= (ADP_cyto)/ (1+ Hi/1000/KaADP + 1/1000/KMgADP);
polyATP=1+ H_mito_1000/KaATP + Mg_1000/KMgATP;
polyADP=1+ H_mito_1000/KaADP + Mg_1000/KMgADP;
polyPi = 1+ H_mito_1000/KaPi;
polyH2O =1+ H_mito_1000/KaH2O;
SUC_poly=1+ H_mito_1000/KaSUC;
%% CPT1
sf_cpt1C16=1;
V_cpt1=1.0E-3;
Kmcpt1_C16CoA_cy=0.0138;
Kmcpt1_Carn_cy=0.125;
Kmcpt1_C16Carn_cy=1.36;
Kmcpt1_CoA_cy=0.407;
Kicpt1_MalCoA=0.0091;
Keq_cpt1=0.45;
n_cpt1=2.4799;
MalCoA_cy=0;
Carn_cy= 0.400;
CoA_cy=1.40;
Carn_mat=0.950;
C16CoA_cy=0.0125;
C16CoA_cyt=C16CoA_cy;
```

```
%CACT:: Parameters
Vf_cact=7.0E-1;
Vr_cact= 7.0E-1;
Kmcact_C16Carn_cy=0.015;
Kmcact_Carn_mat=0.130;
Kmcact_C16Carn_mat=0.15;
Kmcact_Carn_cy=1.30;
Kicact_C16Carn_cy=0.56;
Kicact_Carn_cy=0.200;
Keq_cact=1;
%CPT2:: Parameters
sf_cpt2C16=0.85;
V_cpt2=1.9551E-2;
Kmcpt2_C16Carn_mat=0.051;
Kmcpt2_CoA_mat=0.030;
Kmcpt2_C16CoA_mat=0.038;
Kmcpt2_Carn_mat=0.350;
Keq_cpt2=2.22;
FADt_mat=0.7;
NADt_mat=1.0;
CoAT_mat=1.0;
%VLCAD:: Parameters
sf_vlcadC16=1;
sf_vlcadC14=0.80;
sf_vlcadC12=0.42;
V_vlcad=2.001E-3;
Kmvlcad_C16CoA_mat=0.0065;
Kmvlcad_C14CoA_mat=0.004;
Kmvlcad_C12CoA_mat=0.0027;
Kmvlcad_FAD_mat=1.2E-4;
Kmvlcad_C16EnoylCoA_mat=0.00108;
Kmvlcad_C14EnoylCoA_mat=0.00108;
```

```
Kmvlcad_C12EnoylCoA_mat=0.00108;
Kmvlcad_FADH_mat=0.0242;
Keq_vlcad=6;
%LCAD:: Parameters
sf_lcadC16=0.9;
sf_lcadC14=1;
sf_lcadC12=0.95;%0.9
sf_lcadC10=0.85;%0.75
sf_lcadC8=0.4;
V_lcad=2.499E-3;
Kmlcad_C16CoA_mat=0.0025;
Kmlcad_C14CoA_mat=0.0074;
Kmlcad_C12CoA_mat=0.009;
Kmlcad_C10CoA_mat=0.0243;
Kmlcad_C8CoA_mat=0.123;
Kmlcad_FAD_mat=1.2E-6;
Kmlcad_C16EnoylCoA_mat=0.00108;
Kmlcad_C14EnoylCoA_mat=0.00108;
Kmlcad_C12EnoylCoA_mat=      0.00108;
Kmlcad_C10EnoylCoA_mat=      0.00108;
Kmlcad_C8EnoylCoA_mat=       0.00108;
Kmlcad_FADH_mat=0.0242;
Keq_lcad=6;
%MCAD:: Parameters
sf_mcadC12=0.68;
sf_mcadC10=    0.8;
sf_mcadC8=     0.87;
sf_mcadC6=     1;
sf_mcadC4=     0.12;
V_mcad=0.2505;
Kmmcad_C12CoA_mat=  0.0057;
Kmmcad_C10CoA_mat=  0.0054;
```

Kmmcad_C8CoA_mat= 0.004;

Kmmcad_C6CoA_mat= 0.0094;

Kmmcad_C4CoA_mat= 0.135;

Kmmcad_FAD_mat=1.2E-6;

Kmmcad_C12EnoylCoA_mat= 0.00108;

Kmmcad_C10EnoylCoA_mat= 0.00108;

Kmmcad_C8EnoylCoA_mat= 0.00108;

Kmmcad_C6EnoylCoA_mat= 0.00108;

Kmmcad_C4EnoylCoA_mat= 0.00108;

Kmmcad_FADH_mat=0.0242;

Keq_mcad=6;

%SCAD:: Parameters

sf_scadC6=0.3;

sf_scadC4=1;

V_scad=0.2505;

Kmscad_C6CoA_mat=0.285;

Kmscad_C4CoA_mat= 0.0107;

Kmscad_FAD_mat=1.2E-4;

Kmscad_C6EnoylCoA_mat= 0.00108;

Kmscad_C4EnoylCoA_mat= 0.00108;

Kmscad_FADH_mat=0.0242;

Keq_scad=6;

%CROT:: Parameters

sf_crotC16=0.13;

sf_crotC14= 0.2;

sf_crotC12= 0.25;

sf_crotC10= 0.33;

sf_crotC8= 0.58;

V_crot=2.7E-1;

Kmcrot_C16EnoylCoA_mat= 0.150;

Kmcrot_C14EnoylCoA_mat= 0.100;

Kmcrot_C12EnoylCoA_mat= 0.025;

```
sf_crotC6=          0.8;
sf_crotC4=          1;
Kmcrot_C10EnoylCoA_mat=          0.025;
Kmcrot_C8EnoylCoA_mat=           0.025;
Kmcrot_C6EnoylCoA_mat=           0.025;
Kmcrot_C4EnoylCoA_mat=           0.040;
Kmcrot_C16OHCoA_mat=0.045;
Kmcrot_C14OHCoA_mat=0.045;
Kmcrot_C12OHCoA_mat=0.045;
Kmcrot_C10OHCoA_mat=0.045;
Kmcrot_C8OHCoA_mat=0.045;
Kmcrot_C6OHCoA_mat=0.045;
Kmcrot_C4OHCoA_mat=0.045;
Keq_crot=3.13;
AcacetylCoA_mat=kmrgd(43);
Kicrot_AcacetylCoA_mat=0.0016;
%
%M/SCHAD:: Parameters
sf_mschadC16=0.6;
sf_mschadC14=0.5;
sf_mschadC12=0.43;
sf_mschadC10=  0.64;
sf_mschadC8=   0.89;
sf_mschadC6=   1;
sf_mschadC4=   0.67;
V_mschad=0.5;
Kmmschad_C16OHCoA_mat=       0.0015;
Kmmschad_C14OHCoA_mat=       0.0018;
Kmmschad_C12OHCoA_mat=       0.0037;
Kmmschad_C10OHCoA_mat=       0.0088;
Kmmschad_C8OHCoA_mat=        0.0163;
Kmmschad_C6OHCoA_mat=        0.0286;
```

```
Kmmschad_C4OHCoA_mat=       0.0699;
Kmmschad_NAD_mat=   0.0585;
Kmmschad_C16KetoCoA_mat=    0.0014;
Kmmschad_C14KetoCoA_mat=    0.0014;
Kmmschad_C12KetoCoA_mat=    0.0016;
Kmmschad_C10KetoCoA_mat=    0.0023;
Kmmschad_C8KetoCoA_mat=     0.0041;
Kmmschad_C6KetoCoA_mat=     0.0058;
Kmmschad_C4KetoCoA_mat=     0.0169;
Kmmschad_NADH_mat=  0.0054;
Keq_mschad=2.17*10-4;
%
%MCKAT
sf_mckatC16=0.2;
sf_mckatC14=0.2;
sf_mckatC12=0.38;
sf_mckatC10=0.75;
sf_mckatC8=0.81;
sf_mckatC6=1;
sf_mckatC4=0.49;
V_mckat=0.1884;
Kmmckat_C16KetoCoA_mat=     0.0011;
Kmmckat_C14KetoCoA_mat=     0.0012;
Kmmckat_C12KetoCoA_mat=     0.0013;
Kmmckat_C10KetoCoA_mat=     0.0021;
Kmmckat_C8KetoCoA_mat=      0.0032;
Kmmckat_C6KetoCoA_mat=      0.0067;
Kmmckat_C4KetoCoA_mat=      0.0124;
Kmmckat_CoA_mat=    0.0266;
Kmmckat_C16CoA_mat= 0.01383;
Kmmckat_C14CoA_mat= 0.01383;
Kmmckat_C12CoA_mat= 0.01383;
```

```
Kmmckat_C10CoA_mat=  0.01383;

Kmmckat_C8CoA_mat=   0.01383;

Kmmckat_C6CoA_mat=   0.01383;

Kmmckat_C4CoA_mat=   0.01383;

Kmmckat_AcCoA_mat=   0.030;

Keqmckat=1051;

%

%MTP

sfmtpC16=1.5;

sfmtpC14=0.9;

sfmtpC12=0.81;

sfmtpC10=0.73;

sfmtpC8=0.64;

V_mtp=0.1419;

Kmmtp_C16EnoylCoA_mat=0.025;

Kmmtp_C14EnoylCoA_mat=0.025;

Kmmtp_C12EnoylCoA_mat=0.025;

Kmmtp_C10EnoylCoA_mat=0.025;

Kmmtp_C8EnoylCoA_mat=0.025;

Kmmtp_NAD_mat=       0.060;

Kmmtp_CoA_mat=       0.030;

Kmmtp_C16CoA_mat=    0.01383;

Kmmtp_C14CoA_mat=    0.01383;

Kmmtp_C12CoA_mat=    0.01383;

Kmmtp_C10CoA_mat=    0.01383;

Kmmtp_C8CoA_mat=     0.01383;

Kmmtp_C6CoA_mat=     0.01383;

Kmmtp_NADH_mat=      0.050;

Kmmtp_AcCoA_mat=     0.030;

Kimtp_AcetoacetylCoA_mat= 0.030;

Keqmtp=0.71;

%% Parameters redox, antioxidants....
```

```
kgrxm=3.6E-4;

kgrx=3.6E-4;

KeqGRX=1.37E-3;

GrxT= 0.002;

Vmimac=3.9085E-6;

shunt= 0.01;

EtCuZnSOD=3e-3;

EtMnSOD=3e-3;

NADPmat_T=1.0E-1;

PSSGT= 0.001;        %PSSG total;

kcatPSH= 0.64;       % kcat enzyme PSH + GSH

EtPSH=0.8e-3;

kmGSH=0.75;

kactH2O2=1e-3;       % k activation of H2O2

KmGrx=0.01 ;         % VGRXm, VGRX

KmPSSG= 0.0005;      % VGRXm, VGRX

c_difH2O2=2.0E-4;

c_VGST=1.5E-8;

EtGPXm=10e-5;

EtGRm=9.0E-4;

kcat=1.7E1;

Etcat=1e-6;

EtGPX=5E-5;

kGR=2.5E-3;

EtGR=9.0E-4; %5.0E-3; the GSH scavenging system

EoTxPXm= 0.003;

EoTxPX=0.1;

Phi2Trx= 1.85;       %from Sztajer et al., 2001

Phi1Trx= 3.83;       % en VTxPxm y VTxPx

TrxTm=0.025;         % total amount of TrsSH2m

TrxT= 0.05 ;         % total amount of TrsSH2i

Etxrm=0.00035;       % in VTxRm
```

```
Etxr=0.00035;       % in VTxR

KMTrxSS= 0.035;

KMnadph= 0.012;

kcXR= 22.75E-3;

NADPHc=7.5E-2;

% %

% General parameters and expressions conservation relations

Vlcyt= 4.0; % absolute values are not necessary because they are taken into account when rates are translated into mM ms-1

Vlmat= 1.0; % mitochondrial matrix volume

NAD=NADt_mat-kmrgd(4);

CoA_mat=CoAT_mat-(kmrgd(9)+kmrgd(16)+kmrgd(17)+kmrgd(18)+kmrgd(19)+kmrgd(20)+kmrgd(21)+kmrgd(22)+kmrgd(23)+ …
kmrgd(24)+kmrgd(25)+kmrgd(26)+kmrgd(27)+kmrgd(28)+kmrgd(29)+kmrgd(30)+kmrgd(31)+kmrgd(32)+kmrgd(33)+kmrgd(34)+...
kmrgd(35)+kmrgd(36)+kmrgd(37)+kmrgd(38)+kmrgd(39)+kmrgd(40)+kmrgd(41)+kmrgd(42)+kmrgd(43)+kmrgd(44));

DpH=-log10(1.0E-4)+log10(kmrgd(5));

V4=-2.303*2.670818E1*DpH+kmrgd(3);

%% Metabolic rate expressions TCA cycle and oxidative phosphorilation

VAREN=1.35E18*sqrt(kmrgd(4)/NAD);

VNOden=1/((exp(3.0E2/2.670818E1)+2.077E-18*exp(3.0E2/2.670818E1)*VAREN)+(1.728E-9+1.059E-
26*VAREN)*exp(5.1/2.670818E1*V4));

VHNe=6*RhoREN*(6.394E-13*VAREN-(6.394E-13+1.762E-16)*exp(5.1/2.670818E1*V4))*VNOden;

VNO=0.5*RhoREN*((6.394E-13+2.656E-22*exp(3.0E2/2.670818E1)+8.632E-30*exp(5.1/2.670818E1*V4))*VAREN-6.394E-13* …
exp(5.1/2.670818E1*V4))*VNOden;

Kresf_app=kresf/SUC_poly;

VARSDH=2.670818E1*(log(Kresf_app*sqrt(kmrgd(10)/kmrgd(11))));

VHSDHden=RhoSDH/((1+2.077E-18*exp(VARSDH/2.670818E1))*exp(2.0E2/2.670818E1)+(1.728E-9 + ...

          1.059E-26*exp(VARSDH/2.670818E1))*exp(3.4/2.670818E1*V4))/(1+kmrgd(13)/1.5E-1);

VHSDH=4*(6.394E-13*exp(VARSDH/2.670818E1)-(6.394E-13+1.762E-16)*exp(3.4/2.670818E1*V4))*VHSDHden;

FAD=(FADt_mat-kmrgd(45));

Ketf_app=ketf/polyH2O;

VARETF=2.670818E1*(log(Ketf_app*sqrt(kmrgd(45)/FAD)));

VETFHden=RhoETF/((1+2.077E-18*exp(VARETF/2.670818E1))*exp(2.0E2/2.670818E1)+(1.728E-9 + ...

          1.059E-26*exp(VARETF/2.670818E1))*exp(3.4/2.670818E1*V4));

VETFH=4*(6.394E-13*exp(VARETF/2.670818E1) - (6.394E-13+1.762E-16)*exp(3.4/2.670818E1*V4))*VETFHden;

VO2ETF=5.0E-1*((6.394E-13+2.656E-22*exp(2.0E2/2.670818E1))*exp(VARETF/2.670818E1)+ ...
```

-(6.394E-13-8.632E-30*exp(VARETF/2.670818E1))*exp(3.4/2.670818E1*V4))*VETFHden;

KATPase_app=KeqF1*H_mito_1000*polyATP*polyH2O/(polyADP*polyPi);

VAF1=KATPase_app/kmrgd(6)*(ATPMg/(ADP3+HADP));

VHuden=-RhoF1/(exp(1.5E2/2.670818E1)+1.346E-4*exp(1.5E2/2.670818E1)*VAF1+(7.739E-7+6.65E-15*VAF1)* …
exp(3/2.670818E1*V4));

VATPase=((1.656E-6+9.651E-17*exp(1.5E2/2.670818E1)-4.585E-17*exp(3/2.670818E1*V4))*VAF1-1.656E-8*exp(3/2.670818E1*…
V4)) *VHuden;

Vhu=(3.0E2*1.656E-8+3.0E2*1.656E-8*VAF1-3*(1.656E-8+3.373E-10)*exp(3/2.670818E1*V4))*VHuden;

VANT=VmANT*(1-(ATP4_cyto*ADP3)/(ADP3_cyto*ATP4))*exp(-kmrgd(3)/2.670818E1)/((1+ATP4_cyto/(ADP3_cyto)*exp(-1.8721E-2*..
kmrgd(3)))*(1+ADP3/(ATP4)));

V2FRTdsi=2*(kmrgd(3)-9.1E1)/2.670818E1;

Vuniden=(((1+Cai/1.9E-2)^4)+1.1E2/((1+Cai/3.8E-4)^(2.8)))*(1-exp(-V2FRTdsi));

Vuni=(Vmuni/1.9E-2*Cai*V2FRTdsi*(1+Cai/1.9E-2)^3)/Vuniden;

VnaCa=VmNaCa*exp(2.5E-1*V2FRTdsi)*kmrgd(1)/(Cai*((1+9.4/VNai)^3)*(1+3.75E-4/kmrgd(1)));

Vhleak=gh*(1+15.0*C16CoA_cy^4/(C16CoA_cy^4+2.5E-2^4))*V4;

% TCA cycle rates

VCS=KCS*kmrgd(44)*kmrgd(13)/(KcsiacCoA*KcsOaa*(1+CoA_mat/KcsicoA)+KcsacCoA*(1+CoA_mat/KcsicoA)*kmrgd(13)+KcsOaa*..
kmrgd(44)+kmrgd(44)*kmrgd(13));

VACO =kfACO*(1.3-kmrgd(8)-kmrgd(9)-kmrgd(10)-kmrgd(11)-kmrgd(12)-kmrgd(13)-kmrgd(7)*1.45045);

Va=1/((1+ADP3/KaidADP)*(1+kmrgd(1)/KaidCa));

Vi=1+kmrgd(4)/1.9E-1;

Vb=1.0+kmrgd(5)/kh_1+kh_2/kmrgd(5);

VIDH=kfidh*1.09E-1/(Vb+9.23E-1/NAD*Vi+((1.52/kmrgd(7))^2)*Va*(1+9.23E-1/NAD*Vi));

VKGa=1/((1+1.2987E1)*(1+kmrgd(1)/KkgCa));

VKGDH=kfKG*5E-1/(1.0 + kmrgd(5)/kh_1a+kh_2a/kmrgd(5)+VKGa*(3.0E1/kmrgd(8))^1.2+VKGa*(3.87E1/NAD));

KSLeq_app = KSLeq * SUC_poly * polyATP / (polyADP * polyPi);

VSL=KfSL*(kmrgd(9)*kmrgd(6)*kmrgd(2)-kmrgd(10)*(ATP4+HATP)*CoA_mat/KSLeq_app);

VO2SDH=5.0E-1*((6.394E-13+2.656E-22*exp(2.0E2/2.670818E1))*exp(VARSDH/2.670818E1)+ ...

-(6.394E-13-8.632E-30*exp(VARSDH/2.670818E1))*exp(3.4/2.670818E1*V4))*VHSDHden;

VFH=kfFH*(kmrgd(11)-kmrgd(12)/1);

V26=(1/(1+khm3/kmrgd(5)+khm3*khm4/(kmrgd(5)^2)))^2;

V27=1/(1+kmrgd(5)/khm1+(kmrgd(5)^2)/(khm1*khm2))+kmoff;

VMDH=kfMDH*V26*V27*1.54E-1/(1+1.493/kmrgd(12)*(1+kmrgd(13)/3.1E-2)+2.244E-1/NAD+1.493/kmrgd(12)*(1+kmrgd(13)/3.1E-2)*..
2.244E-1/NAD);

```
VAAT=1.5E-6*6.6*GLU*kmrgd(13)/(1.5E-6*6.6/kfAAT+kmrgd(8));

%% Ionic transport expressions

VNam=-2/3.0E-4*kmrgd(1)-kmrgd(2)-1.812E-3*kmrgd(3)-kmrgd(5)/1.0E-5+kmrgd(6)+1.01E1;

VBeta1p=(1.6E-1*2.4E1*Hi)/(1.585E-4*2.4E1+2.4E1*Hi+1.585E-4*VNai);

VBeta1n=(9.39E-2*2.4E1*kmrgd(5))/(1.585E-4*2.4E1+2.4E1*kmrgd(5)+1.585E-4*VNam);

VBeta2p=(2.52E-2*1.585E-4*VNam)/(1.585E-4*2.4E1+2.4E1*kmrgd(5)+1.585E-4*VNam);

VBeta2n=(4.29E-2*1.585E-4*VNai)/(1.585E-4*2.4E1+2.4E1*Hi+1.585E-4*VNai);

VpHm=-log10(kmrgd(5))+3;

VNaH=Conc_NHE*(((VBeta1p*VBeta2p)-(VBeta1n*VBeta2n))/(VBeta1p+VBeta1n+VBeta2p+VBeta2n))/(1+10^(3*(VpHm-8.52)));

VPiC=VmPiC/60000*(9.0E1*Pi_cyto*1.0E-8/kmrgd(5)/(1.106E1*4.084E-5)-9.0E1*H2Pi*1.0E-4/(1.106E1*4.084E-5))/(1 + …
Pi_cyto/1.106E1+1.0E-8/kmrgd(5)/4.084E-5+Pi_cyto*1.0E-8/kmrgd(5)/(1.106E1*4.084E-5)+H2Pi/1.106E1+1.0E-4/4.084E-5 +…
H2Pi*1.0E-4 /(1.106E1*4.084E-5));

JH=-VHNe - VHSDH - VETFH + Vhu + VNaH + VPiC + Vhleak;

%% Production of oxygen radicals, antioxidant defenses and regeneration of NADPH

VNADPm=NADPmat_T-kmrgd(46);

VdenID_NADP=(1+kmrgd(5)/5.0E-1)*(1+kmrgd(7)/3.9E-3+VNADPm/6.7E-3*(1+2.0E-6/VNADPm)+kmrgd(8)/5.1E-1+kmrgd(46)/1.2E-2
+kmrgd(7)/3.9E-3*VNADPm/6.7E-3*(1+2.0E-6/VNADPm)+kmrgd(8)/5.1E-1*kmrgd(46)/1.2E-2+kmrgd(7)/3.9E-3*kmrgd(46)/1.2E-2
+kmrgd(8)/5.1E-1*VNADPm/6.7E-3 *(1+2.0E-6/VNADPm));

VIDH_NADP=(8.72E-5*kmrgd(7)/3.9E-3*VNADPm/6.7E-3*(1+2.0E-6/VNADPm)-5.45E-6*kmrgd(8)/5.1E-1* …
kmrgd(46)/1.2E-2)/VdenID_NADP;

VDNADP=exp(3.7441E-3*V4);

VDNAD=exp(1-3.7441E-3*V4);

VTHDen=1+kmrgd(4)/1.0E-2+NAD/1.25E-1+VNADPm/1.7E-2+kmrgd(46)/2.0E-2+kmrgd(4)/1.0E-2*VNADPm/1.7E-2*VDNADP +…
kmrgd(46)/2.0E-2*kmrgd(4)/1.0E-2*VDNAD+NAD/1.25E-1*VNADPm/1.7E-2*VDNADP*VDNAD+kmrgd(4)/1.0E-2*kmrgd(46)/2.0E-2;

VTHD=(1.1875E-5*1.174737*kmrgd(4)/1.0E-2*VNADPm/1.7E-2*VDNADP-1.1875E-4*NAD/1.25E-1*kmrgd(46)/2.0E-2*…
VDNAD)/VTHDen;

VIMAC=(1.0E-3+1.0E4/(1+1.0E-2/kmrgd(48)))*(3.5E-8+Vmimac/(1+exp(7.0E-2*(4.0+kmrgd(3)))))*kmrgd(3);

VtrROS=-1.0E-1*(-kmrgd(3)-2.6730818E1*log(kmrgd(47)/kmrgd(48)))/kmrgd(3)*VIMAC;

VMnSOD=2*1.2E3*2.5E-4*(1.2E3+2.4E1*(1+kmrgd(49)/5.0E-1))*EtMnSOD*kmrgd(47)/(2.5E-4*(2*1.2E3+2.4E1*(1+kmrgd(49)/5.0E-1))
+1.2E3*2.4E1*(1+kmrgd(49)/5.0E-1)*kmrgd(47));

VCuZnSOD=2*1.2E3*2.5E-4*(1.2E3+2.4E1*(1+kmrgd(50)/5.0E-1))*EtCuZnSOD*kmrgd(48)/(2.5E-4 *(2*1.2E3+2.4E1*(1+…
kmrgd(50)/5.0E-1))+1.2E3*2.4E1*(1+kmrgd(50)/5.0E-1)*kmrgd(48));

VGPXm=EtGPXm*kmrgd(49)*kmrgd(51)/(5.0E-3*kmrgd(51)+7.5E-1*kmrgd(49));

VGRm=kGR*EtGRm/(1+6.0E-2/kmrgd(53)+1.5E-2/kmrgd(46)+6.0E-2/kmrgd(53)*1.5E-2/kmrgd(46));

VGPX=EtGPX*kmrgd(50)*kmrgd(52)/(5.0E-3*kmrgd(52)+7.5E-1*kmrgd(50));

VGSS=5.0E-1*(6.0-kmrgd(51)-kmrgd(52)-2*kmrgd(53)-kmrgd(56)-kmrgd(57));
```

```
VGR=kGR*EtGR/(1+6.0E-2/VGSS+1.5E-2/NADPHc+6.0E-2/VGSS*1.5E-2/NADPHc);

VGRXm=kgrxm*KeqGRX*kmrgd(51)^2*GrxT*kmrgd(56)/((kmrgd(53)+KeqGRX*kmrgd(51)^2)*(KeqGRX*kmrgd(51)^2*…
GrxT/(kmrgd(53)+KeqGRX*kmrgd(51)^2)+KmGrx)*(kmrgd(56)+KmPSSG));

VGRX=kgrx*KeqGRX*kmrgd(52)^2*GrxT*kmrgd(57)/((VGSS+KeqGRX*kmrgd(52)^2)*(KeqGRX*kmrgd(52)^2*GrxT/(VGSS+ …
KeqGRX*kmrgd(52)^2)+KmGrx)*(kmrgd(57)+KmPSSG));

TrxSSm=TrxTm-kmrgd(54);

TrxSS=TrxT-kmrgd(55);

VTxPXm = EoTxPXm*kmrgd(49)*kmrgd(54)/(Phi2Trx*kmrgd(54)+Phi1Trx*kmrgd(49));

VTxPX = EoTxPX*kmrgd(50)*kmrgd(55)/(Phi2Trx*kmrgd(55)+Phi1Trx*kmrgd(50));

VTxRm = Etxrm*kcXR/(1+KMTrxSS/TrxSSm+KMnadph/kmrgd(46)+KMTrxSS/TrxSSm*KMnadph/kmrgd(46));

VTxR = Etxr*kcXR/(1+KMTrxSS/TrxSS+KMnadph/NADPHc+KMTrxSS/TrxSS*KMnadph/NADPHc);

VdifH2O2=c_difH2O2*(kmrgd(49)-kmrgd(50));

Vcat=2*kcat*Etcat*kmrgd(50)*exp(-5.0E-2*kmrgd(50));

VGST=c_VGST*(kmrgd(52)-kmrgd(51))/(kmrgd(52)+2.6);

VPSSGm=kcatPSH*EtPSH*(PSSGT-kmrgd(56))/(1+(kmGSH/kmrgd(51))/(1+(kmrgd(49)/kactH2O2)));

VPSSGi=kcatPSH*EtPSH*(PSSGT-kmrgd(57))/(1+(kmGSH/kmrgd(52))/(1+(kmrgd(50)/kactH2O2)));

%% beta - Oxidation

Vcpt1C16=sf_cpt1C16*V_cpt1*((C16CoA_cyt*Carn_cy)/(Kmcpt1_C16CoA_cy * Kmcpt1_Carn_cy)-(kmrgd(14)*…
CoA_cy)/(Kmcpt1_C16Carn_cy * Kmcpt1_CoA_cy * Keq_cpt1))/((1+C16CoA_cyt/Kmcpt1_C16CoA_cy +…
kmrgd(14)/Kmcpt1_C16Carn_cy  +(MalCoA_cy/Kicpt1_MalCoA)^(n_cpt1) )*(1+Carn_cy/Kmcpt1_Carn_cy+CoA_cy/Kmcpt1_CoA_cy));

VcactC16=Vf_cact*((kmrgd(14)*Carn_mat-(kmrgd(15)*Carn_cy)/Keq_cact))/(kmrgd(14)*Carn_mat+Kmcact_Carn_mat*kmrgd(14)+…
Kmcact_C16Carn_cy*Carn_mat*(1+Carn_cy/Kicact_Carn_cy)+Vf_cact/(Vr_cact*Keq_cact)*(Kmcact_Carn_cy*kmrgd(15)*(1+…
kmrgd(14)/Kicact_C16Carn_cy)+Carn_cy* (Kmcact_C16Carn_mat+kmrgd(15))));

Vcpt2C16=sf_cpt2C16*V_cpt2*((kmrgd(15)*CoA_mat)/(Kmcpt2_C16Carn_mat*Kmcpt2_CoA_mat)+ …
-(kmrgd(16)*Carn_mat)/(Kmcpt2_C16Carn_mat*Kmcpt2_CoA_mat*Keq_cpt2))/((1+(kmrgd(15)/Kmcpt2_C16Carn_mat+…
kmrgd(16)/Kmcpt2_C16CoA_mat))*(1+CoA_mat/Kmcpt2_CoA_mat+Carn_mat/Kmcpt2_Carn_mat));

VvlcadC16=sf_vlcadC16*V_vlcad*((kmrgd(16) *(FADt_mat-kmrgd(45)))/(Kmvlcad_C16CoA_mat*Kmvlcad_FAD_mat)-(kmrgd(17)*…
kmrgd(45))/(Kmvlcad_C16CoA_mat*Kmvlcad_FAD_mat*Keq_vlcad))/((1+(kmrgd(24)/Kmvlcad_C12CoA_mat+ ...
kmrgd(25)/Kmvlcad_C12EnoylCoA_mat+kmrgd(20)/Kmvlcad_C14CoA_mat+kmrgd(21)/Kmvlcad_C14EnoylCoA_mat+…
kmrgd(16)/Kmvlcad_C16CoA_mat+kmrgd(17)/Kmvlcad_C16EnoylCoA_mat))*(1+(FADt_mat-kmrgd(45))/Kmvlcad_FAD_mat+…
kmrgd(45)/Kmvlcad_FADH_mat));

VvlcadC14=sf_vlcadC14*V_vlcad*((kmrgd(20) *(FADt_mat-kmrgd(45)))/(Kmvlcad_C14CoA_mat*Kmvlcad_FAD_mat)-(kmrgd(21)*…
kmrgd(45))/(Kmvlcad_C14CoA_mat*Kmvlcad_FAD_mat*Keq_vlcad))/((1+(kmrgd(24)/Kmvlcad_C12CoA_mat+…
kmrgd(25)/Kmvlcad_C12EnoylCoA_mat+kmrgd(20)/Kmvlcad_C14CoA_mat+kmrgd(21)/Kmvlcad_C14EnoylCoA_mat+…
kmrgd(16)/Kmvlcad_C16CoA_mat+kmrgd(17)/Kmvlcad_C16EnoylCoA_mat))*(1+(FADt_mat-kmrgd(45))/Kmvlcad_FAD_mat+…
kmrgd(45)/Kmvlcad_FADH_mat));

VvlcadC12=sf_vlcadC12*V_vlcad*((kmrgd(24) *(FADt_mat-kmrgd(45)))/(Kmvlcad_C12CoA_mat*Kmvlcad_FAD_mat)-(kmrgd(25)*…
kmrgd(45))/(Kmvlcad_C12CoA_mat*Kmvlcad_FAD_mat*Keq_vlcad))/((1+(kmrgd(24)/Kmvlcad_C12CoA_mat+ ...
kmrgd(25)/Kmvlcad_C12EnoylCoA_mat+kmrgd(20)/Kmvlcad_C14CoA_mat+kmrgd(21)/Kmvlcad_C14EnoylCoA_mat+...
kmrgd(16)/Kmvlcad_C16CoA_mat+kmrgd(17)/Kmvlcad_C16EnoylCoA_mat))*(1+(FADt_mat-kmrgd(45))/Kmvlcad_FAD_mat+…
kmrgd(45)/Kmvlcad_FADH_mat));
```

```
VlcadC16=sf_lcadC16*V_lcad*((kmrgd(16) *(FADt_mat-kmrgd(45)))/(Kmlcad_C16CoA_mat*Kmlcad_FAD_mat)-(kmrgd(17)*…
kmrgd(45))/(Kmlcad_C16CoA_mat*Kmlcad_FAD_mat*Keq_lcad))/((1+(kmrgd(32)/Kmlcad_C8CoA_mat+…
kmrgd(33)/Kmlcad_C8EnoylCoA_mat+kmrgd(28)/Kmlcad_C10CoA_mat+kmrgd(29)/Kmlcad_C10EnoylCoA_mat+…
kmrgd(24)/Kmlcad_C12CoA_mat+ kmrgd(25)/Kmlcad_C12EnoylCoA_mat+kmrgd(20)/Kmlcad_C14CoA_mat+…
kmrgd(21)/Kmlcad_C14EnoylCoA_mat+kmrgd(16)/Kmlcad_C16CoA_mat+kmrgd(17)/Kmlcad_C16EnoylCoA_mat))*(1+…
(FADt_mat-kmrgd(45))/Kmlcad_FAD_mat+kmrgd(45)/Kmlcad_FADH_mat));

VlcadC14=sf_lcadC14*V_lcad*((kmrgd(20) *(FADt_mat-kmrgd(45)))/(Kmlcad_C14CoA_mat*Kmlcad_FAD_mat)-(kmrgd(21)*…
kmrgd(45))/(Kmlcad_C14CoA_mat*Kmlcad_FAD_mat*Keq_lcad))/((1+(kmrgd(32)/Kmlcad_C8CoA_mat+…
kmrgd(33)/Kmlcad_C8EnoylCoA_mat+kmrgd(28)/Kmlcad_C10CoA_mat+kmrgd(29)/Kmlcad_C10EnoylCoA_mat+…
kmrgd(24)/Kmlcad_C12CoA_mat+ …
kmrgd(25)/Kmlcad_C12EnoylCoA_mat+kmrgd(20)/Kmlcad_C14CoA_mat+kmrgd(21)/Kmlcad_C14EnoylCoA_mat+…
kmrgd(16)/Kmlcad_C16CoA_mat+kmrgd(17)/Kmlcad_C16EnoylCoA_mat))*(1+(FADt_mat-kmrgd(45))/Kmlcad_FAD_mat+…
kmrgd(45)/Kmlcad_FADH_mat));

VlcadC12=sf_lcadC12*V_lcad*((kmrgd(24) *(FADt_mat-kmrgd(45)))/(Kmlcad_C12CoA_mat*Kmlcad_FAD_mat)-(kmrgd(25)*…
kmrgd(45))/(Kmlcad_C12CoA_mat*Kmlcad_FAD_mat*Keq_lcad))/((1+(kmrgd(32)/Kmlcad_C8CoA_mat+…
kmrgd(33)/Kmlcad_C8EnoylCoA_mat+kmrgd(28)/Kmlcad_C10CoA_mat+kmrgd(29)/Kmlcad_C10EnoylCoA_mat+…
kmrgd(24)/Kmlcad_C12CoA_mat+ kmrgd(25)/Kmlcad_C12EnoylCoA_mat+kmrgd(20)/Kmlcad_C14CoA_mat+ …
kmrgd(21)/Kmlcad_C14EnoylCoA_mat+kmrgd(16)/Kmlcad_C16CoA_mat+kmrgd(17)/Kmlcad_C16EnoylCoA_mat))*(1+…
(FADt_mat-kmrgd(45))/Kmlcad_FAD_mat+kmrgd(45)/Kmlcad_FADH_mat));

VlcadC10=sf_lcadC10*V_lcad*((kmrgd(28) *(FADt_mat-kmrgd(45)))/(Kmlcad_C10CoA_mat*Kmlcad_FAD_mat)-(kmrgd(29)*…
kmrgd(45))/(Kmlcad_C10CoA_mat*Kmlcad_FAD_mat*Keq_lcad))/((1+(kmrgd(32)/Kmlcad_C8CoA_mat+…
kmrgd(33)/Kmlcad_C8EnoylCoA_mat+kmrgd(28)/Kmlcad_C10CoA_mat+kmrgd(29)/Kmlcad_C10EnoylCoA_mat+…
kmrgd(24)/Kmlcad_C12CoA_mat+ kmrgd(25)/Kmlcad_C12EnoylCoA_mat+kmrgd(20)/Kmlcad_C14CoA_mat+…
kmrgd(21)/Kmlcad_C14EnoylCoA_mat+kmrgd(16)/Kmlcad_C16CoA_mat+kmrgd(17)/Kmlcad_C16EnoylCoA_mat))*(1+...
(FADt_mat-kmrgd(45))/Kmlcad_FAD_mat+kmrgd(45)/Kmlcad_FADH_mat));

VlcadC8=sf_lcadC8*V_lcad*((kmrgd(32) *(FADt_mat-kmrgd(45)))/(Kmlcad_C8CoA_mat*Kmlcad_FAD_mat)-(kmrgd(33)*…
kmrgd(45))/(Kmlcad_C8CoA_mat*Kmlcad_FAD_mat*Keq_lcad))/((1+(kmrgd(32)/Kmlcad_C8CoA_mat+…
kmrgd(33)/Kmlcad_C8EnoylCoA_mat+kmrgd(28)/Kmlcad_C10CoA_mat+kmrgd(29)/Kmlcad_C10EnoylCoA_mat+…
kmrgd(24)/Kmlcad_C12CoA_mat+ kmrgd(25)/Kmlcad_C12EnoylCoA_mat+kmrgd(20)/Kmlcad_C14CoA_mat+…
kmrgd(21)/Kmlcad_C14EnoylCoA_mat+kmrgd(16)/Kmlcad_C16CoA_mat+kmrgd(17)/Kmlcad_C16EnoylCoA_mat))*(1+...
(FADt_mat-kmrgd(45))/Kmlcad_FAD_mat+kmrgd(45)/Kmlcad_FADH_mat));

VmcadC4=sf_mcadC4*V_mcad*((kmrgd(40) *(FADt_mat-kmrgd(45)))/(Kmmcad_C4CoA_mat*Kmmcad_FAD_mat)-(kmrgd(41)*…
kmrgd(45))/(Kmmcad_C4CoA_mat*Kmmcad_FAD_mat*Keq_mcad))/((1+(kmrgd(40)/Kmmcad_C4CoA_mat+…
kmrgd(41)/Kmmcad_C4EnoylCoA_mat+kmrgd(36)/Kmmcad_C6CoA_mat+kmrgd(37)/Kmmcad_C6EnoylCoA_mat+…
kmrgd(32)/Kmmcad_C8CoA_mat+ kmrgd(33)/Kmmcad_C8EnoylCoA_mat+kmrgd(28)/Kmmcad_C10CoA_mat+…
kmrgd(29)/Kmmcad_C10EnoylCoA_mat+kmrgd(24)/Kmmcad_C12CoA_mat+kmrgd(25)/Kmmcad_C12EnoylCoA_mat))*(1+...
(FADt_mat-kmrgd(45))/Kmmcad_FAD_mat+kmrgd(45)/Kmmcad_FADH_mat));

VmcadC6=sf_mcadC6*V_mcad*((kmrgd(36) *(FADt_mat-kmrgd(45)))/(Kmmcad_C6CoA_mat*Kmmcad_FAD_mat)-(kmrgd(37)*...
kmrgd(45))/(Kmmcad_C6CoA_mat*Kmmcad_FAD_mat*Keq_mcad))/((1+(kmrgd(40)/Kmmcad_C4CoA_mat+…
kmrgd(41)/Kmmcad_C4EnoylCoA_mat+kmrgd(36)/Kmmcad_C6CoA_mat+kmrgd(37)/Kmmcad_C6EnoylCoA_mat+…
kmrgd(32)/Kmmcad_C8CoA_mat+kmrgd(33)/Kmmcad_C8EnoylCoA_mat+kmrgd(28)/Kmmcad_C10CoA_mat+…
kmrgd(29)/Kmmcad_C10EnoylCoA_mat+… kmrgd(24)/Kmmcad_C12CoA_mat+kmrgd(25)/Kmmcad_C12EnoylCoA_mat))*(1+...
(FADt_mat-kmrgd(45))/Kmmcad_FAD_mat+kmrgd(45)/Kmmcad_FADH_mat));

VmcadC8=sf_mcadC8*V_mcad*((kmrgd(32) *(FADt_mat-kmrgd(45)))/(Kmmcad_C8CoA_mat*Kmmcad_FAD_mat)-(kmrgd(33)* ...
kmrgd(45))/(Kmmcad_C8CoA_mat*Kmmcad_FAD_mat*Keq_mcad))/((1+(kmrgd(40)/Kmmcad_C4CoA_mat+…
kmrgd(41)/Kmmcad_C4EnoylCoA_mat+kmrgd(36)/Kmmcad_C6CoA_mat+kmrgd(37)/Kmmcad_C6EnoylCoA_mat+…
kmrgd(32)/Kmmcad_C8CoA_mat+kmrgd(33)/Kmmcad_C8EnoylCoA_mat+kmrgd(28)/Kmmcad_C10CoA_mat+…
kmrgd(29)/Kmmcad_C10EnoylCoA_mat+kmrgd(24)/Kmmcad_C12CoA_mat+kmrgd(25)/Kmmcad_C12EnoylCoA_mat))*(1+...
(FADt_mat-kmrgd(45))/Kmmcad_FAD_mat+kmrgd(45)/Kmmcad_FADH_mat));

VmcadC10=sf_mcadC10*V_mcad*((kmrgd(28) *(FADt_mat-kmrgd(45)))/(Kmmcad_C10CoA_mat*Kmmcad_FAD_mat)-(kmrgd(29)* ...
kmrgd(45))/(Kmmcad_C10CoA_mat*Kmmcad_FAD_mat*Keq_mcad))/((1+(kmrgd(40)/Kmmcad_C4CoA_mat+…
kmrgd(41)/Kmmcad_C4EnoylCoA_mat+kmrgd(36)/Kmmcad_C6CoA_mat+kmrgd(37)/Kmmcad_C6EnoylCoA_mat+…
```

kmrgd(32)/Kmmcad_C8CoA_mat+ kmrgd(33)/Kmmcad_C8EnoylCoA_mat+kmrgd(28)/Kmmcad_C10CoA_mat+…
kmrgd(29)/Kmmcad_C10EnoylCoA_mat+kmrgd(24)/Kmmcad_C12CoA_mat+kmrgd(25)/Kmmcad_C12EnoylCoA_mat))*(1+...
(FADt_mat-kmrgd(45))/Kmmcad_FAD_mat+kmrgd(45)/Kmmcad_FADH_mat));

VmcadC12=sf_mcadC12*V_mcad*((kmrgd(24) *(FADt_mat-kmrgd(45)))/(Kmmcad_C12CoA_mat*Kmmcad_FAD_mat)-(kmrgd(25)* ...
kmrgd(45))/(Kmmcad_C12CoA_mat*Kmmcad_FAD_mat*Keq_mcad))/((1+(kmrgd(40)/Kmmcad_C4CoA_mat+…
kmrgd(41)/Kmmcad_C4EnoylCoA_mat+kmrgd(36)/Kmmcad_C6CoA_mat+kmrgd(37)/Kmmcad_C6EnoylCoA_mat+…
kmrgd(32)/Kmmcad_C8CoA_mat+ kmrgd(33)/Kmmcad_C8EnoylCoA_mat+kmrgd(28)/Kmmcad_C10CoA_mat+…
kmrgd(29)/Kmmcad_C10EnoylCoA_mat+kmrgd(24)/Kmmcad_C12CoA_mat+kmrgd(25)/Kmmcad_C12EnoylCoA_mat))*(1+...
(FADt_mat-kmrgd(45))/Kmmcad_FAD_mat+kmrgd(45)/Kmmcad_FADH_mat));

VscadC4=sf_scadC4*V_scad*((kmrgd(40) *(FADt_mat-kmrgd(45)))/(Kmscad_C4CoA_mat*Kmscad_FAD_mat)-(kmrgd(41)*...
kmrgd(45))/(Kmscad_C4CoA_mat*Kmscad_FAD_mat*Keq_scad))/((1+(kmrgd(40)/Kmscad_C4CoA_mat+…
kmrgd(41)/Kmscad_C4EnoylCoA_mat+kmrgd(36)/Kmscad_C6CoA_mat+kmrgd(37)/Kmscad_C6EnoylCoA_mat))*(1+…
(FADt_mat-kmrgd(45))/Kmscad_FAD_mat+kmrgd(45)/Kmscad_FADH_mat));

VscadC6=sf_scadC6*V_scad*((kmrgd(36) *(FADt_mat-kmrgd(45)))/(Kmscad_C6CoA_mat*Kmscad_FAD_mat)-(kmrgd(37)* ...
kmrgd(45))/(Kmscad_C6CoA_mat*Kmscad_FAD_mat*Keq_scad))/((1+(kmrgd(40)/Kmscad_C4CoA_mat+…
kmrgd(41)/Kmscad_C4EnoylCoA_mat+kmrgd(36)/Kmscad_C6CoA_mat+kmrgd(37)/Kmscad_C6EnoylCoA_mat))*(1+…
(FADt_mat-kmrgd(45))/Kmscad_FAD_mat+kmrgd(45)/Kmscad_FADH_mat));

VcrotC4=sf_crotC4*V_crot*(kmrgd(41)/Kmcrot_C4EnoylCoA_mat-kmrgd(42)/(Kmcrot_C4OHCoA_mat*Keq_crot))/((1+…
(kmrgd(41)/Kmcrot_C4EnoylCoA_mat+kmrgd(42)/Kmcrot_C4OHCoA_mat+kmrgd(37)/Kmcrot_C6EnoylCoA_mat+…
kmrgd(38)/Kmcrot_C6OHCoA_mat+kmrgd(33)/Kmcrot_C8EnoylCoA_mat+kmrgd(34)/Kmcrot_C8OHCoA_mat+…
kmrgd(29)/Kmcrot_C10EnoylCoA_mat+kmrgd(30)/Kmcrot_C10OHCoA_mat+kmrgd(25)/Kmcrot_C12EnoylCoA_mat+…
kmrgd(26)/Kmcrot_C12OHCoA_mat+kmrgd(21)/Kmcrot_C14EnoylCoA_mat+kmrgd(22)/Kmcrot_C14OHCoA_mat+…
kmrgd(17)/Kmcrot_C16EnoylCoA_mat+kmrgd(18)/Kmcrot_C16OHCoA_mat))+(AcacetylCoA_mat/Kicrot_AcacetylCoA_mat));

VcrotC6=sf_crotC6*V_crot*(kmrgd(37)/Kmcrot_C6EnoylCoA_mat-kmrgd(38)/(Kmcrot_C6OHCoA_mat*Keq_crot)) /((1+…
(kmrgd(41)/Kmcrot_C4EnoylCoA_mat+kmrgd(42)/Kmcrot_C4OHCoA_mat+kmrgd(37)/Kmcrot_C6EnoylCoA_mat+…
kmrgd(38)/Kmcrot_C6OHCoA_mat+kmrgd(33)/Kmcrot_C8EnoylCoA_mat+kmrgd(34)/Kmcrot_C8OHCoA_mat+…
kmrgd(29)/Kmcrot_C10EnoylCoA_mat+kmrgd(30)/Kmcrot_C10OHCoA_mat+kmrgd(25)/Kmcrot_C12EnoylCoA_mat+…
kmrgd(26)/Kmcrot_C12OHCoA_mat+kmrgd(21)/Kmcrot_C14EnoylCoA_mat+kmrgd(22)/Kmcrot_C14OHCoA_mat+…
kmrgd(17)/Kmcrot_C16EnoylCoA_mat+kmrgd(18)/Kmcrot_C16OHCoA_mat))+(AcacetylCoA_mat/Kicrot_AcacetylCoA_mat));

VcrotC8=sf_crotC8*V_crot*(kmrgd(33)/Kmcrot_C8EnoylCoA_mat-kmrgd(34)/(Kmcrot_C8OHCoA_mat*Keq_crot))/((1+…
(kmrgd(41)/Kmcrot_C4EnoylCoA_mat+kmrgd(42)/Kmcrot_C4OHCoA_mat+kmrgd(37)/Kmcrot_C6EnoylCoA_mat+…
kmrgd(38)/Kmcrot_C6OHCoA_mat+kmrgd(33)/Kmcrot_C8EnoylCoA_mat+kmrgd(34)/Kmcrot_C8OHCoA_mat+…
kmrgd(29)/Kmcrot_C10EnoylCoA_mat+kmrgd(30)/Kmcrot_C10OHCoA_mat+kmrgd(25)/Kmcrot_C12EnoylCoA_mat+…
kmrgd(26)/Kmcrot_C12OHCoA_mat+kmrgd(21)/Kmcrot_C14EnoylCoA_mat+kmrgd(22)/Kmcrot_C14OHCoA_mat+…
kmrgd(17)/Kmcrot_C16EnoylCoA_mat+kmrgd(18)/Kmcrot_C16OHCoA_mat))+(AcacetylCoA_mat/Kicrot_AcacetylCoA_mat));

VcrotC10=sf_crotC10*V_crot*(kmrgd(29)/Kmcrot_C10EnoylCoA_mat-kmrgd(30)/(Kmcrot_C10OHCoA_mat*Keq_crot))/((1+…
(kmrgd(41)/Kmcrot_C4EnoylCoA_mat+kmrgd(42)/Kmcrot_C4OHCoA_mat+kmrgd(37)/Kmcrot_C6EnoylCoA_mat+…
kmrgd(38)/Kmcrot_C6OHCoA_mat+kmrgd(33)/Kmcrot_C8EnoylCoA_mat+kmrgd(34)/Kmcrot_C8OHCoA_mat+…
kmrgd(29)/Kmcrot_C10EnoylCoA_mat+kmrgd(30)/Kmcrot_C10OHCoA_mat+kmrgd(25)/Kmcrot_C12EnoylCoA_mat+…
kmrgd(26)/Kmcrot_C12OHCoA_mat+kmrgd(21)/Kmcrot_C14EnoylCoA_mat+kmrgd(22)/Kmcrot_C14OHCoA_mat+…
kmrgd(17)/Kmcrot_C16EnoylCoA_mat+kmrgd(18)/Kmcrot_C16OHCoA_mat))+(AcacetylCoA_mat/Kicrot_AcacetylCoA_mat));

VcrotC12=sf_crotC12*V_crot*(kmrgd(25)/Kmcrot_C12EnoylCoA_mat-kmrgd(26)/(Kmcrot_C12OHCoA_mat*Keq_crot)) /((1+…
(kmrgd(41)/Kmcrot_C4EnoylCoA_mat+kmrgd(42)/Kmcrot_C4OHCoA_mat+kmrgd(37)/Kmcrot_C6EnoylCoA_mat+…
kmrgd(38)/Kmcrot_C6OHCoA_mat+kmrgd(33)/Kmcrot_C8EnoylCoA_mat+kmrgd(34)/Kmcrot_C8OHCoA_mat+…
kmrgd(29)/Kmcrot_C10EnoylCoA_mat+kmrgd(30)/Kmcrot_C10OHCoA_mat+kmrgd(25)/Kmcrot_C12EnoylCoA_mat+…
kmrgd(26)/Kmcrot_C12OHCoA_mat+kmrgd(21)/Kmcrot_C14EnoylCoA_mat+kmrgd(22)/Kmcrot_C14OHCoA_mat+…
kmrgd(17)/Kmcrot_C16EnoylCoA_mat+kmrgd(18)/Kmcrot_C16OHCoA_mat))+(AcacetylCoA_mat/Kicrot_AcacetylCoA_mat));

VcrotC14=sf_crotC14*V_crot*(kmrgd(21)/Kmcrot_C14EnoylCoA_mat-kmrgd(22)/(Kmcrot_C14OHCoA_mat*Keq_crot)) /((1+…
(kmrgd(41)/Kmcrot_C4EnoylCoA_mat+kmrgd(42)/Kmcrot_C4OHCoA_mat+kmrgd(37)/Kmcrot_C6EnoylCoA_mat+…
kmrgd(38)/Kmcrot_C6OHCoA_mat+kmrgd(33)/Kmcrot_C8EnoylCoA_mat+kmrgd(34)/Kmcrot_C8OHCoA_mat+…
kmrgd(29)/Kmcrot_C10EnoylCoA_mat+kmrgd(30)/Kmcrot_C10OHCoA_mat+kmrgd(25)/Kmcrot_C12EnoylCoA_mat+…

kmrgd(26)/Kmcrot_C12OHCoA_mat+kmrgd(21)/Kmcrot_C14EnoylCoA_mat+kmrgd(22)/Kmcrot_C14OHCoA_mat+…
kmrgd(17)/Kmcrot_C16EnoylCoA_mat+kmrgd(18)/Kmcrot_C16OHCoA_mat))+(AcacetylCoA_mat/Kicrot_AcacetylCoA_mat));

VcrotC16=sf_crotC16*V_crot*(kmrgd(17)/Kmcrot_C16EnoylCoA_mat-kmrgd(18)/(Kmcrot_C16OHCoA_mat*Keq_crot)) /((1+…
(kmrgd(41)/Kmcrot_C4EnoylCoA_mat+kmrgd(42)/Kmcrot_C4OHCoA_mat+kmrgd(37)/Kmcrot_C6EnoylCoA_mat+…
kmrgd(38)/Kmcrot_C6OHCoA_mat+kmrgd(33)/Kmcrot_C8EnoylCoA_mat+kmrgd(34)/Kmcrot_C8OHCoA_mat+…
kmrgd(29)/Kmcrot_C10EnoylCoA_mat+kmrgd(30)/Kmcrot_C10OHCoA_mat+kmrgd(25)/Kmcrot_C12EnoylCoA_mat+…
kmrgd(26)/Kmcrot_C12OHCoA_mat+kmrgd(21)/Kmcrot_C14EnoylCoA_mat+kmrgd(22)/Kmcrot_C14OHCoA_mat+…
kmrgd(17)/Kmcrot_C16EnoylCoA_mat+kmrgd(18)/Kmcrot_C16OHCoA_mat))+(AcacetylCoA_mat/Kicrot_AcacetylCoA_mat));

VmschadC4=sf_mschadC4*V_mschad*((kmrgd(42)*(NADt_mat-kmrgd(4)))/(Kmmschad_C4OHCoA_mat*Kmmschad_NAD_mat)+…
-(kmrgd(43)*kmrgd(4))/(Kmmschad_C4OHCoA_mat*Kmmschad_NAD_mat*Keq_mschad))/((1+...
(kmrgd(42)/Kmmschad_C4OHCoA_mat+kmrgd(43)/Kmmschad_C4KetoCoA_mat+kmrgd(38)/Kmmschad_C6OHCoA_mat+…
kmrgd(39)/Kmmschad_C6KetoCoA_mat+kmrgd(34)/Kmmschad_C8OHCoA_mat+kmrgd(35)/Kmmschad_C8KetoCoA_mat+...
kmrgd(30)/Kmmschad_C10OHCoA_mat+kmrgd(31)/Kmmschad_C10KetoCoA_mat+kmrgd(26)/Kmmschad_C12OHCoA_mat+…
kmrgd(27)/Kmmschad_C12KetoCoA_mat+kmrgd(22)/Kmmschad_C14OHCoA_mat+kmrgd(23)/Kmmschad_C14KetoCoA_mat+…
kmrgd(18)/Kmmschad_C16OHCoA_mat+kmrgd(19)/Kmmschad_C16KetoCoA_mat))*(1+…
(NADt_mat-kmrgd(4))/Kmmschad_NAD_mat+kmrgd(4)/Kmmschad_NADH_mat));

VmschadC6=sf_mschadC6*V_mschad*((kmrgd(38) *(NADt_mat-kmrgd(4)))/(Kmmschad_C6OHCoA_mat*Kmmschad_NAD_mat)+…
-(kmrgd(39)*kmrgd(4))/(Kmmschad_C6OHCoA_mat*Kmmschad_NAD_mat*Keq_mschad))/((1+...
(kmrgd(42)/Kmmschad_C4OHCoA_mat+kmrgd(43)/Kmmschad_C4KetoCoA_mat+kmrgd(38)/Kmmschad_C6OHCoA_mat+…
kmrgd(39)/Kmmschad_C6KetoCoA_mat+kmrgd(34)/Kmmschad_C8OHCoA_mat+kmrgd(35)/Kmmschad_C8KetoCoA_mat+...
kmrgd(30)/Kmmschad_C10OHCoA_mat+kmrgd(31)/Kmmschad_C10KetoCoA_mat+kmrgd(26)/Kmmschad_C12OHCoA_mat+…
kmrgd(27)/Kmmschad_C12KetoCoA_mat+kmrgd(22)/Kmmschad_C14OHCoA_mat+kmrgd(23)/Kmmschad_C14KetoCoA_mat+…
kmrgd(18)/Kmmschad_C16OHCoA_mat+kmrgd(19)/Kmmschad_C16KetoCoA_mat))*(1+…
(NADt_mat-kmrgd(4))/Kmmschad_NAD_mat+kmrgd(4)/Kmmschad_NADH_mat));

VmschadC8=sf_mschadC8*V_mschad*((kmrgd(34) *(NADt_mat-kmrgd(4)))/(Kmmschad_C8OHCoA_mat*Kmmschad_NAD_mat)+….
-(kmrgd(35)*kmrgd(4))/(Kmmschad_C8OHCoA_mat*Kmmschad_NAD_mat*Keq_mschad))/((1+...
(kmrgd(42)/Kmmschad_C4OHCoA_mat+kmrgd(43)/Kmmschad_C4KetoCoA_mat+kmrgd(38)/Kmmschad_C6OHCoA_mat+…
kmrgd(39)/Kmmschad_C6KetoCoA_mat+kmrgd(34)/Kmmschad_C8OHCoA_mat+kmrgd(35)/Kmmschad_C8KetoCoA_mat+...
kmrgd(30)/Kmmschad_C10OHCoA_mat+kmrgd(31)/Kmmschad_C10KetoCoA_mat+kmrgd(26)/Kmmschad_C12OHCoA_mat+…
kmrgd(27)/Kmmschad_C12KetoCoA_mat+kmrgd(22)/Kmmschad_C14OHCoA_mat+kmrgd(23)/Kmmschad_C14KetoCoA_mat+…
kmrgd(18)/Kmmschad_C16OHCoA_mat+kmrgd(19)/Kmmschad_C16KetoCoA_mat))*(1+…
(NADt_mat-kmrgd(4))/Kmmschad_NAD_mat+kmrgd(4)/Kmmschad_NADH_mat));

VmschadC10=sf_mschadC10*V_mschad*((kmrgd(30)*(NADt_mat-kmrgd(4)))/(Kmmschad_C10OHCoA_mat*Kmmschad_NAD_mat)+..
-(kmrgd(31)*kmrgd(4))/(Kmmschad_C10OHCoA_mat*Kmmschad_NAD_mat*Keq_mschad)) /((1+...
(kmrgd(42)/Kmmschad_C4OHCoA_mat+kmrgd(43)/Kmmschad_C4KetoCoA_mat+kmrgd(38)/Kmmschad_C6OHCoA_mat+…
kmrgd(39)/Kmmschad_C6KetoCoA_mat+kmrgd(34)/Kmmschad_C8OHCoA_mat+kmrgd(35)/Kmmschad_C8KetoCoA_mat+...
kmrgd(30)/Kmmschad_C10OHCoA_mat+kmrgd(31)/Kmmschad_C10KetoCoA_mat+kmrgd(26)/Kmmschad_C12OHCoA_mat+…
kmrgd(27)/Kmmschad_C12KetoCoA_mat+kmrgd(22)/Kmmschad_C14OHCoA_mat+kmrgd(23)/Kmmschad_C14KetoCoA_mat+…
kmrgd(18)/Kmmschad_C16OHCoA_mat+kmrgd(19)/Kmmschad_C16KetoCoA_mat))*(1+…
(NADt_mat-kmrgd(4))/Kmmschad_NAD_mat+kmrgd(4)/Kmmschad_NADH_mat));

VmschadC12=sf_mschadC12*V_mschad*((kmrgd(26)*(NADt_mat-kmrgd(4)))/(Kmmschad_C12OHCoA_mat*Kmmschad_NAD_mat)+..
-(kmrgd(27)*kmrgd(4))/(Kmmschad_C12OHCoA_mat*Kmmschad_NAD_mat*Keq_mschad)) /((1+...
(kmrgd(42)/Kmmschad_C4OHCoA_mat+kmrgd(43)/Kmmschad_C4KetoCoA_mat+kmrgd(38)/Kmmschad_C6OHCoA_mat+…
kmrgd(39)/Kmmschad_C6KetoCoA_mat+kmrgd(34)/Kmmschad_C8OHCoA_mat+kmrgd(35)/Kmmschad_C8KetoCoA_mat+...
kmrgd(30)/Kmmschad_C10OHCoA_mat+kmrgd(31)/Kmmschad_C10KetoCoA_mat+kmrgd(26)/Kmmschad_C12OHCoA_mat+…
kmrgd(27)/Kmmschad_C12KetoCoA_mat+kmrgd(22)/Kmmschad_C14OHCoA_mat+kmrgd(23)/Kmmschad_C14KetoCoA_mat+…
kmrgd(18)/Kmmschad_C16OHCoA_mat+kmrgd(19)/Kmmschad_C16KetoCoA_mat))*(1+…
(NADt_mat-kmrgd(4))/Kmmschad_NAD_mat+kmrgd(4)/Kmmschad_NADH_mat));

VmschadC14=sf_mschadC14*V_mschad*((kmrgd(22) *(NADt_mat-kmrgd(4)))/(Kmmschad_C14OHCoA_mat*Kmmschad_NAD_mat)+.
-(kmrgd(23)*kmrgd(4))/(Kmmschad_C14OHCoA_mat*Kmmschad_NAD_mat*Keq_mschad))/((1+...
(kmrgd(42)/Kmmschad_C4OHCoA_mat+kmrgd(43)/Kmmschad_C4KetoCoA_mat+kmrgd(38)/Kmmschad_C6OHCoA_mat+…
kmrgd(39)/Kmmschad_C6KetoCoA_mat+kmrgd(34)/Kmmschad_C8OHCoA_mat+kmrgd(35)/Kmmschad_C8KetoCoA_mat+...
kmrgd(30)/Kmmschad_C10OHCoA_mat+kmrgd(31)/Kmmschad_C10KetoCoA_mat+kmrgd(26)/Kmmschad_C12OHCoA_mat+…

```
kmrgd(27)/Kmmschad_C12KetoCoA_mat+kmrgd(22)/Kmmschad_C14OHCoA_mat+kmrgd(23)/Kmmschad_C14KetoCoA_mat+…
kmrgd(18)/Kmmschad_C16OHCoA_mat+kmrgd(19)/Kmmschad_C16KetoCoA_mat))*(1+…
(NADt_mat-kmrgd(4))/Kmmschad_NAD_mat+kmrgd(4)/Kmmschad_NADH_mat));

VmschadC16=sf_mschadC16*V_mschad*((kmrgd(18) *(NADt_mat-kmrgd(4)))/(Kmmschad_C16OHCoA_mat*Kmmschad_NAD_mat)+.
-(kmrgd(19)*kmrgd(4))/(Kmmschad_C16OHCoA_mat*Kmmschad_NAD_mat*Keq_mschad)) /((1+...
(kmrgd(42)/Kmmschad_C4OHCoA_mat+kmrgd(43)/Kmmschad_C4KetoCoA_mat+kmrgd(38)/Kmmschad_C6OHCoA_mat+…
kmrgd(39)/Kmmschad_C6KetoCoA_mat+kmrgd(34)/Kmmschad_C8OHCoA_mat+kmrgd(35)/Kmmschad_C8KetoCoA_mat+...
kmrgd(30)/Kmmschad_C10OHCoA_mat+kmrgd(31)/Kmmschad_C10KetoCoA_mat+kmrgd(26)/Kmmschad_C12OHCoA_mat+…
kmrgd(27)/Kmmschad_C12KetoCoA_mat+kmrgd(22)/Kmmschad_C14OHCoA_mat+kmrgd(23)/Kmmschad_C14KetoCoA_mat+…
kmrgd(18)/Kmmschad_C16OHCoA_mat+kmrgd(19)/Kmmschad_C16KetoCoA_mat))*(1+…
(NADt_mat-kmrgd(4))/Kmmschad_NAD_mat+kmrgd(4)/Kmmschad_NADH_mat));

VmckatC16= sf_mckatC16*V_mckat*((kmrgd(19)*CoA_mat)/(Kmmckat_C16KetoCoA_mat*Kmmckat_CoA_mat)-(kmrgd(20)*…
kmrgd(44))/(Kmmckat_C16KetoCoA_mat*Kmmckat_CoA_mat*Keqmckat))/(1+((kmrgd(19)/Kmmckat_C16KetoCoA_mat+…
kmrgd(16)/Kmmckat_C16CoA_mat+kmrgd(23)/Kmmckat_C14KetoCoA_mat+kmrgd(20)/Kmmckat_C14CoA_mat+...
kmrgd(27)/Kmmckat_C12KetoCoA_mat+kmrgd(24)/Kmmckat_C12CoA_mat+kmrgd(31)/Kmmckat_C10KetoCoA_mat+…
kmrgd(28)/Kmmckat_C10CoA_mat+kmrgd(35)/Kmmckat_C8KetoCoA_mat+kmrgd(32)/Kmmckat_C8CoA_mat+…
kmrgd(39)/Kmmckat_C6KetoCoA_mat+kmrgd(36)/Kmmckat_C6CoA_mat+kmrgd(43)/Kmmckat_C4KetoCoA_mat+…
kmrgd(40)/Kmmckat_C4CoA_mat)+kmrgd(44)/Kmmckat_AcCoA_mat)*(1+CoA_mat/Kmmckat_CoA_mat+…
kmrgd(44)/Kmmckat_AcCoA_mat));

VmckatC14= sf_mckatC14*V_mckat*((kmrgd(23)*CoA_mat)/(Kmmckat_C14KetoCoA_mat*Kmmckat_CoA_mat)-(kmrgd(24)*…
kmrgd(44))/(Kmmckat_C14KetoCoA_mat*Kmmckat_CoA_mat*Keqmckat))/(1+((kmrgd(19)/Kmmckat_C16KetoCoA_mat+…
kmrgd(16)/Kmmckat_C16CoA_mat+kmrgd(23)/Kmmckat_C14KetoCoA_mat+kmrgd(20)/Kmmckat_C14CoA_mat+...
kmrgd(27)/Kmmckat_C12KetoCoA_mat+kmrgd(24)/Kmmckat_C12CoA_mat+kmrgd(31)/Kmmckat_C10KetoCoA_mat+…
kmrgd(28)/Kmmckat_C10CoA_mat+kmrgd(35)/Kmmckat_C8KetoCoA_mat+kmrgd(32)/Kmmckat_C8CoA_mat+…
kmrgd(39)/Kmmckat_C6KetoCoA_mat+kmrgd(36)/Kmmckat_C6CoA_mat+kmrgd(43)/Kmmckat_C4KetoCoA_mat+…
kmrgd(40)/Kmmckat_C4CoA_mat)+kmrgd(44)/Kmmckat_AcCoA_mat)*(1+CoA_mat/Kmmckat_CoA_mat+…
kmrgd(44)/Kmmckat_AcCoA_mat));

VmckatC12= sf_mckatC12*V_mckat*((kmrgd(27)*CoA_mat)/(Kmmckat_C12KetoCoA_mat*Kmmckat_CoA_mat)-(kmrgd(28)*…
kmrgd(44))/(Kmmckat_C12KetoCoA_mat*Kmmckat_CoA_mat*Keqmckat))/(1+((kmrgd(19)/Kmmckat_C16KetoCoA_mat+…
kmrgd(16)/Kmmckat_C16CoA_mat+kmrgd(23)/Kmmckat_C14KetoCoA_mat+kmrgd(20)/Kmmckat_C14CoA_mat+...
kmrgd(27)/Kmmckat_C12KetoCoA_mat+kmrgd(24)/Kmmckat_C12CoA_mat+kmrgd(31)/Kmmckat_C10KetoCoA_mat+…
kmrgd(28)/Kmmckat_C10CoA_mat+kmrgd(35)/Kmmckat_C8KetoCoA_mat+kmrgd(32)/Kmmckat_C8CoA_mat+…
kmrgd(39)/Kmmckat_C6KetoCoA_mat+kmrgd(36)/Kmmckat_C6CoA_mat+kmrgd(43)/Kmmckat_C4KetoCoA_mat+…
kmrgd(40)/Kmmckat_C4CoA_mat)+kmrgd(44)/Kmmckat_AcCoA_mat)*(1+CoA_mat/Kmmckat_CoA_mat+…
kmrgd(44)/Kmmckat_AcCoA_mat));

VmckatC10= sf_mckatC10*V_mckat*((kmrgd(31)*CoA_mat)/(Kmmckat_C10KetoCoA_mat*Kmmckat_CoA_mat)-(kmrgd(32)*…
kmrgd(44))/(Kmmckat_C10KetoCoA_mat*Kmmckat_CoA_mat*Keqmckat))/(1+((kmrgd(19)/Kmmckat_C16KetoCoA_mat+…
kmrgd(16)/Kmmckat_C16CoA_mat+kmrgd(23)/Kmmckat_C14KetoCoA_mat+kmrgd(20)/Kmmckat_C14CoA_mat+...
kmrgd(27)/Kmmckat_C12KetoCoA_mat+kmrgd(24)/Kmmckat_C12CoA_mat+kmrgd(31)/Kmmckat_C10KetoCoA_mat+…
kmrgd(28)/Kmmckat_C10CoA_mat+kmrgd(35)/Kmmckat_C8KetoCoA_mat+kmrgd(32)/Kmmckat_C8CoA_mat+…
kmrgd(39)/Kmmckat_C6KetoCoA_mat+kmrgd(36)/Kmmckat_C6CoA_mat+kmrgd(43)/Kmmckat_C4KetoCoA_mat+…
kmrgd(40)/Kmmckat_C4CoA_mat)+kmrgd(44)/Kmmckat_AcCoA_mat)*(1+CoA_mat/Kmmckat_CoA_mat+…
kmrgd(44)/Kmmckat_AcCoA_mat));

VmckatC8= sf_mckatC8*V_mckat*((kmrgd(35)*CoA_mat)/(Kmmckat_C8KetoCoA_mat*Kmmckat_CoA_mat)-(kmrgd(36)*…
kmrgd(44))/(Kmmckat_C8KetoCoA_mat*Kmmckat_CoA_mat*Keqmckat))/(1+((kmrgd(19)/Kmmckat_C16KetoCoA_mat+…
kmrgd(16)/Kmmckat_C16CoA_mat+kmrgd(23)/Kmmckat_C14KetoCoA_mat+kmrgd(20)/Kmmckat_C14CoA_mat+...
kmrgd(27)/Kmmckat_C12KetoCoA_mat+kmrgd(24)/Kmmckat_C12CoA_mat+kmrgd(31)/Kmmckat_C10KetoCoA_mat+…
kmrgd(28)/Kmmckat_C10CoA_mat+kmrgd(35)/Kmmckat_C8KetoCoA_mat+kmrgd(32)/Kmmckat_C8CoA_mat+…
kmrgd(39)/Kmmckat_C6KetoCoA_mat+kmrgd(36)/Kmmckat_C6CoA_mat+kmrgd(43)/Kmmckat_C4KetoCoA_mat+…
kmrgd(40)/Kmmckat_C4CoA_mat)+kmrgd(44)/Kmmckat_AcCoA_mat)*(1+CoA_mat/Kmmckat_CoA_mat+…
kmrgd(44)/Kmmckat_AcCoA_mat));

VmckatC6= sf_mckatC6*V_mckat*((kmrgd(39)*CoA_mat)/(Kmmckat_C6KetoCoA_mat*Kmmckat_CoA_mat)-(kmrgd(40)*…
kmrgd(44))/(Kmmckat_C6KetoCoA_mat*Kmmckat_CoA_mat*Keqmckat))/(1+((kmrgd(19)/Kmmckat_C16KetoCoA_mat+…
```

kmrgd(16)/Kmmckat_C16CoA_mat+kmrgd(23)/Kmmckat_C14KetoCoA_mat+kmrgd(20)/Kmmckat_C14CoA_mat+...
kmrgd(27)/Kmmckat_C12KetoCoA_mat+kmrgd(24)/Kmmckat_C12CoA_mat+kmrgd(31)/Kmmckat_C10KetoCoA_mat+…
kmrgd(28)/Kmmckat_C10CoA_mat+kmrgd(35)/Kmmckat_C8KetoCoA_mat+kmrgd(32)/Kmmckat_C8CoA_mat+…
kmrgd(39)/Kmmckat_C6KetoCoA_mat+kmrgd(36)/Kmmckat_C6CoA_mat+kmrgd(43)/Kmmckat_C4KetoCoA_mat+…
kmrgd(40)/Kmmckat_C4CoA_mat)+kmrgd(44)/Kmmckat_AcCoA_mat)*(1+CoA_mat/Kmmckat_CoA_mat+…
kmrgd(44)/Kmmckat_AcCoA_mat));

VmckatC4= sf_mckatC4*V_mckat*((kmrgd(43)*CoA_mat)/(Kmmckat_C4KetoCoA_mat*Kmmckat_CoA_mat)-(kmrgd(44)*…
kmrgd(44))/(Kmmckat_C4KetoCoA_mat*Kmmckat_CoA_mat*Keqmckat))/(1+((kmrgd(19)/Kmmckat_C16KetoCoA_mat+…
kmrgd(16)/Kmmckat_C16CoA_mat+kmrgd(23)/Kmmckat_C14KetoCoA_mat+kmrgd(20)/Kmmckat_C14CoA_mat+...
kmrgd(27)/Kmmckat_C12KetoCoA_mat+kmrgd(24)/Kmmckat_C12CoA_mat+kmrgd(31)/Kmmckat_C10KetoCoA_mat+…
kmrgd(28)/Kmmckat_C10CoA_mat+kmrgd(35)/Kmmckat_C8KetoCoA_mat+kmrgd(32)/Kmmckat_C8CoA_mat+…
kmrgd(39)/Kmmckat_C6KetoCoA_mat+kmrgd(36)/Kmmckat_C6CoA_mat+kmrgd(43)/Kmmckat_C4KetoCoA_mat+…
kmrgd(40)/Kmmckat_C4CoA_mat)+kmrgd(44)/Kmmckat_AcCoA_mat)*(1+CoA_mat/Kmmckat_CoA_mat+…
kmrgd(44)/Kmmckat_AcCoA_mat));

VmtpC16=sfmtpC16*V_mtp*((kmrgd(17)*(NADt_mat-kmrgd(4))*CoA_mat)/(Kmmtp_C16EnoylCoA_mat*Kmmtp_NAD_mat*…
Kmmtp_CoA_mat)-kmrgd(20)*kmrgd(4)*kmrgd(44)/(Kmmtp_C16EnoylCoA_mat*Kmmtp_NAD_mat* Kmmtp_CoA_mat*…
Keqmtp))/((1+(kmrgd(17)/Kmmtp_C16EnoylCoA_mat+kmrgd(16)/Kmmtp_C16CoA_mat+kmrgd(21)/Kmmtp_C14EnoylCoA_mat+…
kmrgd(20)/Kmmtp_C14CoA_mat+kmrgd(25)/Kmmtp_C12EnoylCoA_mat+kmrgd(24)/Kmmtp_C12CoA_mat+…
kmrgd(29)/Kmmtp_C10EnoylCoA_mat+kmrgd(28)/Kmmtp_C10CoA_mat +kmrgd(33)/Kmmtp_C8EnoylCoA_mat+…
kmrgd(32)/Kmmtp_C8CoA_mat) +kmrgd(36)/Kmmtp_C6CoA_mat+AcacetylCoA_mat/Kimtp_AcetoacetylCoA_mat)*(1+(NADt_mat+…
-kmrgd(4))/Kmmtp_NAD_mat+kmrgd(4)/Kmmtp_NADH_mat)*(1+CoA_mat/Kmmtp_CoA_mat+kmrgd(44)/Kmmtp_AcCoA_mat));

VmtpC14=sfmtpC14*V_mtp*((kmrgd(21)*(NADt_mat-kmrgd(4))*CoA_mat)/(Kmmtp_C14EnoylCoA_mat*Kmmtp_NAD_mat*…
Kmmtp_CoA_mat)-kmrgd(24)*kmrgd(4)*kmrgd(44)/(Kmmtp_C14EnoylCoA_mat*Kmmtp_NAD_mat*Kmmtp_CoA_mat*…
Keqmtp))/((1+(kmrgd(17)/Kmmtp_C16EnoylCoA_mat+kmrgd(16)/Kmmtp_C16CoA_mat+kmrgd(21)/Kmmtp_C14EnoylCoA_mat+…
kmrgd(20)/Kmmtp_C14CoA_mat+kmrgd(25)/Kmmtp_C12EnoylCoA_mat+kmrgd(24)/Kmmtp_C12CoA_mat+…
kmrgd(29)/Kmmtp_C10EnoylCoA_mat+kmrgd(28)/Kmmtp_C10CoA_mat +kmrgd(33)/Kmmtp_C8EnoylCoA_mat+…
kmrgd(32)/Kmmtp_C8CoA_mat) +kmrgd(36)/Kmmtp_C6CoA_mat+AcacetylCoA_mat/Kimtp_AcetoacetylCoA_mat)*(1+(NADt_mat+…
-kmrgd(4))/Kmmtp_NAD_mat+kmrgd(4)/Kmmtp_NADH_mat)*(1+CoA_mat/Kmmtp_CoA_mat+kmrgd(44)/Kmmtp_AcCoA_mat));

VmtpC12=sfmtpC12*V_mtp*((kmrgd(25)*(NADt_mat-kmrgd(4))*CoA_mat)/(Kmmtp_C12EnoylCoA_mat*Kmmtp_NAD_mat*…
Kmmtp_CoA_mat)-kmrgd(28)*kmrgd(4)*kmrgd(44)/(Kmmtp_C12EnoylCoA_mat*Kmmtp_NAD_mat*Kmmtp_CoA_mat*….
Keqmtp))/((1+(kmrgd(17)/Kmmtp_C16EnoylCoA_mat+kmrgd(16)/Kmmtp_C16CoA_mat+kmrgd(21)/Kmmtp_C14EnoylCoA_mat+…
kmrgd(20)/Kmmtp_C14CoA_mat+kmrgd(25)/Kmmtp_C12EnoylCoA_mat+kmrgd(24)/Kmmtp_C12CoA_mat+…
kmrgd(29)/Kmmtp_C10EnoylCoA_mat+kmrgd(28)/Kmmtp_C10CoA_mat +kmrgd(33)/Kmmtp_C8EnoylCoA_mat+…
kmrgd(32)/Kmmtp_C8CoA_mat) +kmrgd(36)/Kmmtp_C6CoA_mat+AcacetylCoA_mat/Kimtp_AcetoacetylCoA_mat)*(1+(NADt_mat+…
-kmrgd(4))/Kmmtp_NAD_mat+kmrgd(4)/Kmmtp_NADH_mat)*(1+CoA_mat/Kmmtp_CoA_mat+kmrgd(44)/Kmmtp_AcCoA_mat));

VmtpC10=sfmtpC10*V_mtp*((kmrgd(29)*(NADt_mat-kmrgd(4))*CoA_mat)/(Kmmtp_C10EnoylCoA_mat*Kmmtp_NAD_mat*…
Kmmtp_CoA_mat)-kmrgd(32)*kmrgd(4)*kmrgd(44)/(Kmmtp_C10EnoylCoA_mat*Kmmtp_NAD_mat*Kmmtp_CoA_mat*…
Keqmtp))/((1+(kmrgd(17)/Kmmtp_C16EnoylCoA_mat+kmrgd(16)/Kmmtp_C16CoA_mat+kmrgd(21)/Kmmtp_C14EnoylCoA_mat+…
kmrgd(20)/Kmmtp_C14CoA_mat+kmrgd(25)/Kmmtp_C12EnoylCoA_mat+kmrgd(24)/Kmmtp_C12CoA_mat+…
kmrgd(29)/Kmmtp_C10EnoylCoA_mat+kmrgd(28)/Kmmtp_C10CoA_mat +kmrgd(33)/Kmmtp_C8EnoylCoA_mat+…
kmrgd(32)/Kmmtp_C8CoA_mat) +kmrgd(36)/Kmmtp_C6CoA_mat+AcacetylCoA_mat/Kimtp_AcetoacetylCoA_mat)*(1+(NADt_mat+…
-kmrgd(4))/Kmmtp_NAD_mat+kmrgd(4)/Kmmtp_NADH_mat)*(1+CoA_mat/Kmmtp_CoA_mat+kmrgd(44)/Kmmtp_AcCoA_mat));

VmtpC8=sfmtpC8*V_mtp*((kmrgd(33)*(NADt_mat-kmrgd(4))*CoA_mat)/(Kmmtp_C8EnoylCoA_mat*Kmmtp_NAD_mat*…
Kmmtp_CoA_mat)-kmrgd(36)*kmrgd(4)*kmrgd(44)/(Kmmtp_C8EnoylCoA_mat*Kmmtp_NAD_mat*Kmmtp_CoA_mat*…
Keqmtp))/((1+(kmrgd(17)/Kmmtp_C16EnoylCoA_mat+kmrgd(16)/Kmmtp_C16CoA_mat+kmrgd(21)/Kmmtp_C14EnoylCoA_mat+…
kmrgd(20)/Kmmtp_C14CoA_mat+kmrgd(25)/Kmmtp_C12EnoylCoA_mat+kmrgd(24)/Kmmtp_C12CoA_mat+…
kmrgd(29)/Kmmtp_C10EnoylCoA_mat+kmrgd(28)/Kmmtp_C10CoA_mat +kmrgd(33)/Kmmtp_C8EnoylCoA_mat+…
kmrgd(32)/Kmmtp_C8CoA_mat) +kmrgd(36)/Kmmtp_C6CoA_mat+AcacetylCoA_mat/Kimtp_AcetoacetylCoA_mat)*(1+(NADt_mat+…
-kmrgd(4))/Kmmtp_NAD_mat+kmrgd(4)/Kmmtp_NADH_mat)*(1+CoA_mat/Kmmtp_CoA_mat+kmrgd(44)/Kmmtp_AcCoA_mat));

%% Ordinary differential equations

dydt=[fCam*(Vuni-VnaCa);   %Cam (1)

```
VANT-VATPase-VSL;                     %ADPm (2)
-(-VHNe-VHSDH-VETFH+Vhu+VANT+Vhleak+VnaCa+2*Vuni+VIMAC)/Cimat;% Dpsim (3)
-VNO+VIDH+VKGDH+VMDH-VTHD+(VmtpC16+VmschadC16+VmtpC14+VmschadC14+VmtpC12+VmschadC12+...
VmtpC10+VmschadC10+VmtpC8+VmschadC8+VmschadC6+VmschadC4)/Vlmat;          %NADHm (4)
beta_matr*(JH);                       %Hm    (5)
-VATPase+VPiC-VSL;                    %Pim   (6)
VACO-VIDH-VIDH_NADP;                  %ISO   (7)
VIDH+VAAT-VKGDH+VIDH_NADP;            %aKG   (8)
VKGDH-VSL;                            %SCoA  (9)
VSL-VO2SDH;                           %Succ  (10)
VO2SDH-VFH;                           %FUM   (11)
VFH-VMDH;                             %MAL   (12)
VMDH-VCS-VAAT;                        %Oaa   (13)
(Vcpt1C16-VcactC16)/Vlcyt;                    %C16AcylCarn_cy   (14)
(VcactC16-Vcpt2C16)/Vlmat;                    %C16Carn_mat  (15)
(Vcpt2C16-VvlcadC16-VlcadC16)/Vlmat;          %C16CoA_mat   (16)
(VvlcadC16+VlcadC16-VcrotC16-VmtpC16)/Vlmat;          %C16EnoylCoA_mat  (17)
(VcrotC16-VmschadC16)/Vlmat;                  %C16OHCoA_mat  (18)
(VmschadC16-VmckatC16)/Vlmat;                 %C16KetoCoA_mat (19)
(VmtpC16+VmckatC16-VvlcadC14-VlcadC14)/Vlmat;         %C14CoA_mat    (20)
(VvlcadC14+VlcadC14-VcrotC14-VmtpC14)/Vlmat;          %C14EnoylCoA_mat (21)
(VcrotC14-VmschadC14)/Vlmat;                  %C14OHCoA_mat   (22)
(VmschadC14-VmckatC14)/Vlmat;                 %C14KetoCoA_mat  (23)
(VmtpC14+VmckatC14-VvlcadC12-VlcadC12-VmcadC12)/Vlmat;   %C12CoA_mat     (24)
(VvlcadC12+VlcadC12+VmcadC12-VcrotC12-VmtpC12)/Vlmat;    %C12EnoylCoA_mat (25)
(VcrotC12-VmschadC12)/Vlmat;                  %C12OHCoA_mat   (26)
(VmschadC12-VmckatC12)/Vlmat;                 %C12KetoCoA_mat  (27)
(VmtpC12+VmckatC12-VmcadC10-VlcadC10)/Vlmat;          %C10CoA_mat     (28)
(VmcadC10+VlcadC10-VcrotC10-VmtpC10)/Vlmat;           %C10EnoylCoA_mat (29)
(VcrotC10-VmschadC10)/Vlmat;                  %C10OHCoA_mat   (30)
(VmschadC10-VmckatC10)/Vlmat;                 %C10KetoCoA_mat  (31)
(VmtpC10+VmckatC10-VmcadC8-VlcadC8)/Vlmat;            %C8CoA_mat      (32)
```

```
(VmcadC8+VlcadC8-VcrotC8-VmtpC8)/Vlmat;                    %C8EnoylCoA_mat  (33)

(VcrotC8-VmschadC8)/Vlmat;                                 %C8OHCoA_mat     (34)

(VmschadC8-VmckatC8)/Vlmat;                                %C8KetoCoA_mat   (35)

(VmtpC8+VmckatC8-VmcadC6-VscadC6)/Vlmat;                   %C6CoA_mat       (36)

(VmcadC6+VscadC6-VcrotC6)/Vlmat;                           %C6EnoylCoA_mat  (37)

(VcrotC6-VmschadC6)/Vlmat;                                 %C6OHCoA_mat     (38)

(VmschadC6-VmckatC6)/Vlmat;                                %C6KetoCoA_mat (39)

(VmckatC6-VmcadC4-VscadC4)/Vlmat;                          %C4CoA_mat   (40)

(VmcadC4+VscadC4-VcrotC4)/Vlmat;                           %C4EnoylCoA_mat (41)

(VcrotC4-VmschadC4)/Vlmat;                                 %C4OHCoA_mat  (42)

(VmschadC4-VmckatC4)/Vlmat;                                %C4KetoCoA_mat  (43)

(VmtpC16+VmckatC16+VmtpC14+VmckatC14+VmtpC12+VmckatC12+VmtpC10+VmckatC10+VmtpC8+VmckatC8+VmckatC6+…
2*VmckatC4)/Vlmat-VCS;% AcCoA_mat     (44)

(VvlcadC16+VlcadC16+VvlcadC14+VlcadC14+VvlcadC12+VlcadC12+VmcadC12+VlcadC10+VmcadC10+VlcadC8+VmcadC8+…
VmcadC6+VscadC6+VmcadC4+VscadC4)/Vlmat-VO2ETF;                         % FADH2_mat (45)

VIDH_NADP+VTHD-VGRm-VTxRm;              %NADPH  (46)

shunt*(VNO+VO2SDH+VO2ETF)-VMnSOD-VtrROS;%SO2m (47)

Vlmat/Vlcyt*VtrROS-VCuZnSOD;            %SO2i  (48)

VMnSOD-VdifH2O2-VGPXm-VTxPXm;           %H2O2m (49)

VCuZnSOD+Vlmat/Vlcyt*VdifH2O2-VGPX-VTxPX-Vcat;  %H2O2i (50)

VGRm-VGPXm-VGRXm+VGST-VPSSGm;           %GSHm   (51)

VGR-VGPX-VGRX-Vlmat/Vlcyt*VGST-VPSSGi;  %GSHi   (52)

5.0D-1*(VGPXm-VGRm)+VGRXm;              %GSSGm  (53)

VTxRm-VTxPXm;                           %TrxSH2m  (54)

VTxR-VTxPX;                             %TrxSH2    (55)

VPSSGm-VGRXm;                           %PSSGm     (56)

VPSSGi-VGRX;];                          %PSSGi    (57)

return
```
